# Global burden and trends of non-communicable diseases among children and adolescents from 1990 to 2021: an age-period-cohort and frontier analysis with projections to 2035

**DOI:** 10.3389/fpubh.2026.1698005

**Published:** 2026-07-03

**Authors:** Ying Chen, Yudan Long, Binjie Zheng, Jinhang Lei, Xinyang Zhang, Zhouzhou Lin, Ziyue Fu, Kai Liu

**Affiliations:** 1 Haina Medical University, Haikou, China; 2 Medical Laboratory Center, Hainan Medical University, Hainan General Hospital, Hainan Affiliated Hospital of Hainan Medical University, Haikou, China; 3 Geriatric Center, Hainan General Hospital, Hainan Affiliated Hospital of Hainan Medical University, Hainan Medical University, Haikou, China

**Keywords:** adolescents, age-period-cohort analysis, children, frontier analysis, global burden of disease, health inequalities, non-communicable diseases

## Abstract

**Background:**

Comprehensive analyses of non-communicable diseases (NCDs) in children and adolescents that disentangle age, period, and cohort effects, assess health system efficiency, and project future trends remain limited. This study aims to address these gaps using data from the Global Burden of Disease Study 2021.

**Methods:**

Utilizing data from GBD 2021, we analyzed age-standardized rates (ASRs) of prevalence, incidence, mortality, and disability-adjusted life years (DALYs) for NCDs. Analyses included joinpoint regression to assess trends, age-period-cohort (APC) modeling to distinguish age, period, and birth cohort effects, frontier analysis to evaluate health system efficiency relative to socio-demographic index (SDI), and Bayesian APC modeling to project future burden to 2035. Inequalities were measured using the Slope Index of Inequality and Concentration Index.

**Results:**

From 1990 to 2021, while global age-standardized mortality rate (ASMR) and DALY rate (ASDR) decreased substantially (AAPC:−2.20% and−1.30%, respectively), the age-standardized incidence rate (ASIR) increased (AAPC: +0.05%). The age-standardized prevalence rate (ASPR) remained largely stable (AAPC:−0.02%). Notably, the burden of mental disorders and diabetes and kidney diseases rose significantly. Adolescents (15–19 years) experienced increasing ASPR, contrasting with declines in younger children. Marked disparities were observed: lower SDI regions carried the highest burden, yet higher SDI regions showed rising ASIR. APC analysis confirmed strong age effects and modest cohort improvements. Frontier analysis identified significant efficiency gaps, even among high-SDI countries. Projections suggest a rising incidence in the 5–14 year age groups by 2035, alongside continued declines in mortality and DALYs.

**Conclusion:**

The shifting NCD burden toward non-fatal morbidity, especially mental and metabolic conditions in adolescents, requires developmentally tailored interventions. Addressing health system inefficiencies and socioeconomic disparities is crucial to reduce the future NCD burden and achieve Sustainable Development Goals.

## 1 Introduction

In recent years, the global burden of non-communicable diseases (NCDs) among children and adolescents has garnered increasing attention and has become a major public health challenge (1, 2). The Global Burden of Disease (GBD) 2021 study indicates that NCDs pose a significant and evolving health threat to individuals aged 0–19 years (3, 4). These diseases, including metabolic diseases, mental health disorders, neurological diseases, and cardiovascular diseases, not only severely impair immediate health, development, and quality of life but may also persist into adulthood, leading to substantial long-term health and socioeconomic burdens (5, 6). According to the Lancet Commission on Adolescent Health and Wellbeing report “Our Future: A Lancet Commission on Adolescent Health and Wellbeing”, although adolescents have seen improvements in traditional health issues such as infectious diseases compared to 1990, NCDs—primarily mental health issues, obesity, and cardiovascular disease risks—have become increasingly prominent (7).

Although successive GBD studies have provided comprehensive data, critical analytical gaps regarding the NCD burden in this young population remain. Existing reports often focus on overall trends and lack in-depth exploration of the underlying drivers (8, 9). Specifically, studies that systematically disentangle the independent effects of age, period, and birth cohort (APC) are scarce. Furthermore, robust assessments comparing the efficiency of health systems in managing childhood NCDs across different socioeconomic contexts, using methods such as frontier analysis, are very limited. Additionally, the disease spectrum is undergoing subtle changes—the rising burden of lifestyle-related NCDs (such as mental and metabolic disorders) may offset the gains from preventing and controlling other NCDs, necessitating precise characterization. Elucidating these dynamics is crucial for anticipating future challenges and optimizing resource allocation.

Therefore, advanced analytical methods beyond descriptive epidemiology are needed. The Age-Period-Cohort (APC) model can decode the complex effects of development, environment, and intergenerational interactions on disease risk (10, 11). Frontier analysis can evaluate health system performance, identify countries achieving the best outcomes with given resources, and highlight inefficiencies (12, 13). Combined with long-term forecasting, these approaches can comprehensively outline the evolving trajectory of childhood NCDs, directly supporting the strategic formulation of health equity and NCD prevention within the Sustainable Development Goals (SDGs).

This study aims to fill the aforementioned gaps using the GBD 2021 database. We hypothesize that significant and modifiable disparities in the NCD burden exist across different socioeconomic strata and age groups, driven by variations in exposure and health system efficiency. Without targeted interventions, the burden of specific NCD subtypes is projected to continue rising. The specific objectives are: to quantify the global, regional, and national burden and trends of NCDs among children and adolescents from 1990 to 2021; to decompose the observed trends into age, period, and cohort effects using APC models; to employ frontier analysis to assess the performance and efficiency of health systems in managing childhood NCDs across countries; to predict the incidence, prevalence, mortality, and DALYs of NCDs in this population up to 2035 using the Bayesian APC model; and to evaluate socioeconomic inequalities and identify key determinants shaping the current and future landscape of childhood NCDs. By integrating these multidimensional analyses, this study aims to provide evidence-based guidance for precise public health interventions, optimize the allocation of medical resources, and contribute to achieving global health equity goals for children and adolescents.

## 2 Methods

### 2.1 Data source

The 2021 Global Burden of Disease (GBD 2021) study recently provided an updated and comprehensive overview of global health loss, covering 371 diseases, injuries, and impairments across 204 countries and territories, stratified by age and sex (14). Detailed analytical frameworks, computational methods, and data sources have been described in previous publications (15, 16). For this analysis, we obtained data from the publicly accessible GBD 2021 database (available at http://ghdx.healthdata.org/gbd-results-tool) for the period 1980–2021, including overall non-communicable diseases and their major subtypes: cardiovascular diseases, chronic respiratory diseases, diabetes and kidney diseases, digestive diseases, mental disorders, musculoskeletal disorders, neoplasms, neurological disorders, sense organ diseases, skin and subcutaneous diseases, substance use disorders, and other non-communicable diseases. Based on socioeconomic development levels, countries were categorized into low, low-middle, middle, high-middle, or high Socio-demographic Index (SDI) groups according to SDI quintiles. The Socio-demographic Index (SDI), developed by the Global Burden of Disease study team, is a composite indicator designed to quantify socioeconomic development levels and establish standardized stratification for disease burden research. By revealing systematic correlations between health issues and socioeconomic factors, it establishes a more explanatory analytical framework. Compared to traditional measurement systems, its innovation lies in incorporating fertility parameters to characterize demographic transition stages and adopting a multidimensional approach to integrate core economic, educational, and demographic elements. GBD 2021 used de-identified aggregated data for analysis. The Institutional Review Board of the University of Washington reviewed and approved the waiver of informed consent. According to the definition of the World Health Organization, children and adolescents refer to people between the ages of 0 and 19 years old. According to the GBD hierarchical classification system, Level 1 represents broad disease categories (e.g., non-communicable diseases), while Level 2 includes specific disease groups within these categories, such as cardiovascular diseases, mental disorders, diabetes and kidney diseases, and neoplasms.

### 2.2 Statistical analysis and data quality control

All estimates, including age-standardized rates (ASRs) of prevalence, incidence, mortality, and disability-adjusted life years (DALYs) for Level 1 and Level 2 non-communicable diseases among children and adolescents, were derived from the Global Burden of Disease (GBD) 2021 study. The GBD study employs a standardized, iterative analytical process for data extraction, adjustment, and modeling to ensure comparability and minimize bias (17, 18). This process includes systematic data identification from a wide range of sources, rigorous data quality and completeness checks, cross-walking to correct for differences in case definitions and measurement instruments, and the use of advanced statistical models (e.g., DisMod-MR 2.1, spatiotemporal Gaussian process regression) to address data sparsity and ensure consistency across estimates (19, 20). All data are presented as absolute values with their 95% uncertainty intervals (UIs), and ASRs are expressed per 100,000 population, stratified by age, sex, region, country, and year.

Joinpoint regression models were used to evaluate the temporal trends in ASRs from 1990 to 2021. The average annual percentage change (AAPC) and its 95% confidence interval (CI) were calculated by fitting regression lines to the natural logarithm of the rates with the calendar year as the regressor. A trend was classified as increasing if the AAPC and its 95% CI were above zero, decreasing if below zero, and stable otherwise. Analyses were stratified by four age groups: 0–4 years, 5–9 years, 10–14 years, and 15–19 years. Additionally, Spearman's correlation analysis was used to examine the relationship between age-standardized rates and the Socio-demographic Index (SDI), with a *P*-value less than 0.05 considered statistically significant.

All statistical analyses and data visualizations were performed using R software (version 4.4.2). The JD_GBD package (V2.37, Jingding Medical Technology Co., Ltd.) was utilized for data extraction and initial processing, while standard R packages (e.g., joinpoint, ggplot2) were employed for specific analyses and figure creation.

### 2.3 Joinpoint analysis

We employed Joinpoint regression analysis to examine the temporal trends of ASPR (Age-Standardized Prevalence Rate), ASIR (Age-Standardized Incidence Rate), ASMR (Age-Standardized Mortality Rate), and ASDR (Age-Standardized Disability-Adjusted Life Year Rate) from 1990 to 2019. This method enabled us to identify significant changes in trends over time, referred to as “joinpoints,” and to estimate the annual percentage change (APC) for each segment defined by these joinpoints. For each of these four metrics, we fitted a linear regression model using the calendar year as the independent variable and the logarithm of the rate as the dependent variable. The model accommodated zero values and variances proportional to the mean rate through a Poisson distribution. Joinpoints were identified through a series of permutation tests, and the final number of joinpoints was selected based on the Bayesian information criterion. The analysis provided APC estimates along with their corresponding 95% confidence intervals (CIs) for each segment, offering insights into the direction and magnitude of trends during the study period. Detailed methods are provided in the Supplementary Methods.

### 2.4 Health inequality analysis

This study utilized the Slope Index of Inequality (SII) and the Concentration Index (CI) to measure absolute and relative inequalities in disease burden, respectively, following the analytical methods recommended by the World Health Organization (WHO) for health inequality monitoring (21). These indices were used to quantify the distributional disparities in the burden of non-communicable diseases among children and adolescents across countries and regions, providing a comprehensive assessment of health inequalities. Specifically, we calculated SII and CI for each of the four primary outcome measures: age-standardized prevalence rate (ASPR), incidence rate (ASIR), mortality rate (ASMR), and disability-adjusted life years rate (ASDR). The SII was derived from a robust regression model, regressing each age-standardized rate against the Socio-demographic Index (SDI), where the SDI was represented by the midpoint of the cumulative population distribution ranked from the least to the most advantaged. To analyze temporal changes in health inequalities, we compared data from 204 countries and territories from 1990 to 2021. Given the potential for outliers and heterogeneous data inherent in cross-national analyses, we employed a robust regression model using an M-estimator with Tukey's biweight function, instead of ordinary least squares regression. This approach reduces the undue influence of outliers and provides more reliable estimates of the socioeconomic gradient in disease burden. Similarly, the CI was calculated by plotting the cumulative proportion of each age-standardized rate against the cumulative population distribution, ranked by SDI, and numerically integrating the area under the resulting concentration curve. The entire analytical framework aligns with established practices in the Global Burden of Disease (GBD) studies (22, 23).

### 2.5 Age-period-cohort analysis

In this study, we utilized the Age-Period-Cohort Analysis Tool provided by the National Cancer Institute (http://analysistools.cancer.gov/apc) to evaluate the independent effects of age, period, and birth cohort on non-communicable diseases (NCDs) in children and adolescents. Specifically, data from 1992 to 2021 were used for the APC analysis to ensure the construction of complete and symmetric 5-year period intervals (1992–1996 to 2017–2021), which is required for stable estimation in age–period–cohort modeling. Age groups were recoded into four 5-year intervals: 0–4 years, 5–9 years, 10–14 years, and 15–19 years, enabling a more precise analysis of disease trends across different age groups. Additionally, corresponding birth years were recoded into 5-year intervals (e.g., 1897–1901, 1902–1906, etc.) to more accurately estimate the impact of birth cohorts on disease burden. Using the intrinsic estimator (IE), we calculated ratios comparing specific ages, periods, or birth cohorts to the average levels across all ages, periods, and cohorts, thereby revealing the independent contributions of these factors to disease burden. This analytical approach provided a comprehensive and in-depth understanding of the trends in non-communicable diseases among children and adolescents across different time periods, age groups, and birth cohorts, offering robust scientific evidence for the formulation of public health policies.

### 2.6 Bayesian age-period-cohort (BAPC) model

Building on the Age-Period-Cohort (APC) model analysis, this study further employed the Bayesian Age-Period-Cohort (BAPC) model to project the future incidence trends of NCD in children and adolescents. The BAPC model addresses the non-identifiability issue inherent in the classical APC framework—due to the exact linear dependence among age, period, and cohort effects—by incorporating Bayesian smoothing priors and utilizing the Integrated Nested Laplace Approximation (INLA) algorithm for estimation (24, 25). The model is specified as follows:


$$\begin{array}{l} Log\left(Y_{\text{ijk}}\right)=\;=\text{μ}+{\text{α}}_{\text{i}}+{\text{β}}_{\text{j}}+\gamma_{\text{k}}+{\text{ϵ}}_{\text{ijk}} \end{array}$$

where (Y_ijk_) represents the observed SCI incidence in the (i) th age group, (j) th period, and (k) th birth cohort; μ is the overall mean; α_i_, β_j_, γ_k_ represent the age, period, and cohort effects, respectively; and ϵ_ijk_ is the error term.

To enforce smoothness over the temporal dimensions, we assigned second-order random walk (RW2) priors to the age, period, and cohort effects. Vague, conjugate priors—specifically, Gamma (1, 0.00005) priors—were placed on the precision parameters of the RW2 structures. This prior choice is standard in Bayesian disease mapping and GBD-related projections, as it imposes reasonable smoothness while allowing the data to predominantly drive the estimation. The model was implemented using the BAPC and INLA packages in R. To assess the predictive validity and robustness of our model before making future projections, we performed a temporal validation exercise. We fitted the model on data from 1990 to 2015 and projected the incidence for the period 2016–2021. The projected trends and their 95% credible intervals were then compared against the observed data for this holdout period, demonstrating good calibration. Subsequently, the final model was fitted on the complete dataset from 1990 to 2021 to project the burden of NCD among children and adolescents from 2022 to 2035, with all projections presented with their corresponding 95% credible intervals to quantify uncertainty.

### 2.7 Frontier analysis

To evaluate the relationship between the burden of non-communicable diseases (NCDs) in children and adolescents and the level of socio-demographic development, we employed non-parametric efficiency frontier analysis as a quantitative method. This approach identifies the potentially achievable minimum age-standardized incidence rate (ASIR) based on the developmental status, measured by the Socio-demographic Index (SDI). This frontier is not a causal boundary but an efficiency frontier, used to characterize the theoretically attainable lowest number of incident cases at a given SDI level. The distance to this frontier is termed the “efficiency gap”; a larger efficiency gap indicates that, given a country or region's position on the development spectrum, there remains unrealized potential for reducing the incidence of NCDs among children and adolescents.

We constructed a Data Envelopment Analysis (DEA) approach, utilizing the Free Disposal Hull (FDH) model to delineate a non-linear frontier. Based on data from 1990 to 2021, we established the frontier relationship between SDI and the age-standardized incidence of NCDs in children and adolescents. To account for uncertainty, we drew 1,000 bootstrap samples with replacement from all countries and territories across all years. For each bootstrap sample, we calculated the mean age-standardized incidence rate of NCDs in children and adolescents corresponding to each SDI value. Subsequently, we applied Local Linear Regression (LOESS, with local polynomial degree = 1 and span = 0.2) to generate a smoothed frontier.

“Super-efficient” units were defined as countries or regions whose ASR fell below the mean frontier value minus three times the standard deviation derived from 95% of the bootstrap samples at the same SDI level, within the FDH framework (26). Excluding these outliers prevents the frontier from being unduly lowered by individual, non-replicable extreme observations, thereby ensuring the boundary represents an efficiency level achievable by the “majority” of countries under existing developmental conditions, rather than a theoretical limit.

To understand the position of each country or territory's age-standardized incidence rate of NCDs in children and adolescents relative to the frontier in 2021, we calculated the “efficiency gap” (i.e., the absolute distance to the frontier) for each using 2021 SDI and incidence rate data. For countries or regions whose incidence rates were already below the frontier, the efficiency gap was recorded as zero.

## 3 Results

### 3.1 Global trends

**Figure 1:**
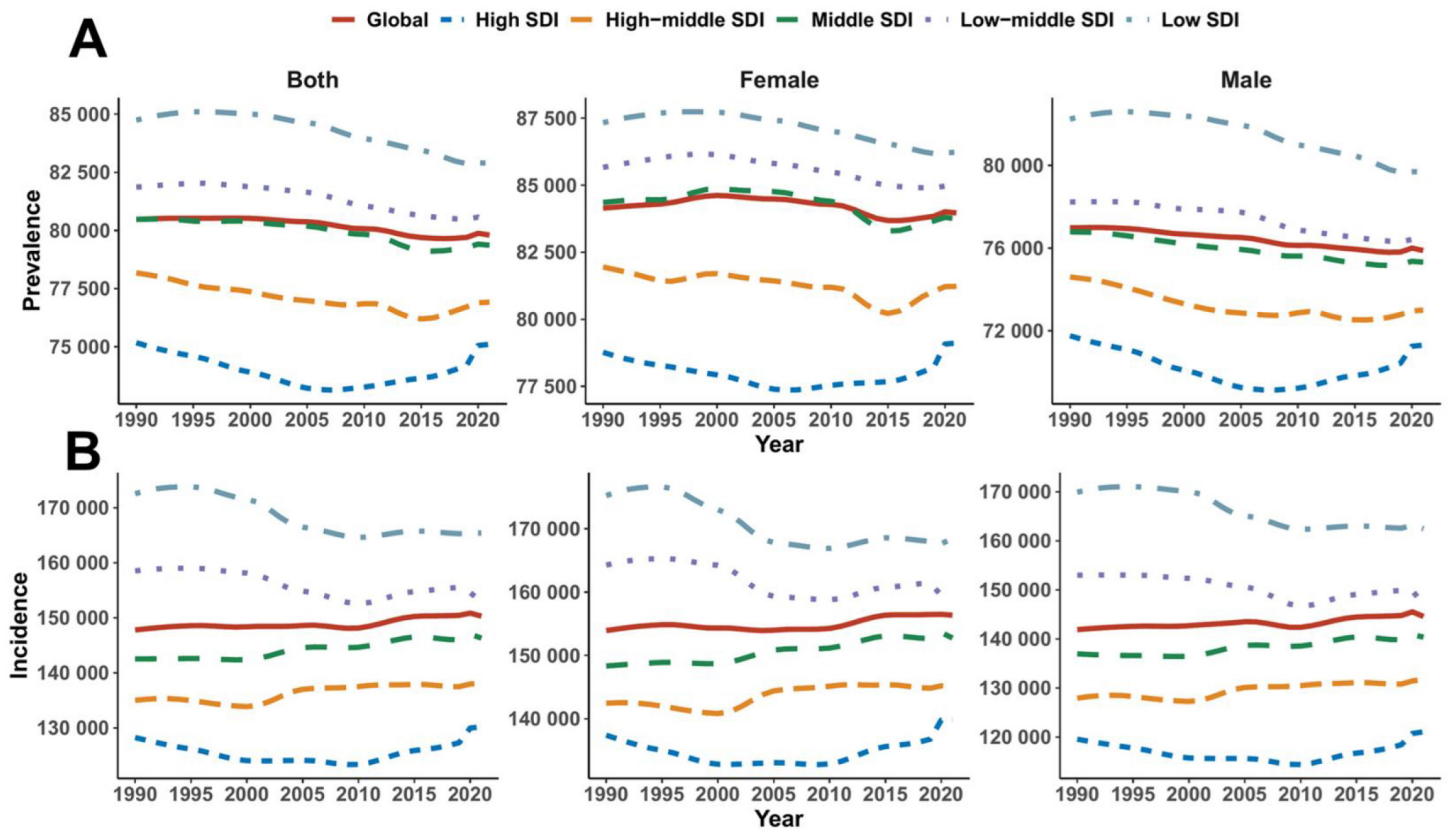
Trends in age-standardized rates of non-communicable diseases (NCDs) among children and adolescents globally from 1990 to 2021. **(A)** Age-standardized prevalence rate. **(B)** Age-standardized incidence rate.

From 1990 to 2021, the global age-standardized prevalence rate (ASPR) of non-communicable diseases (NCDs) among children and adolescents showed a slight but statistically significant decrease, with an average annual percentage change (AAPC) of−0.02% (95% CI:−0.03 to−0.02, *P* < 0.001) (Table 1, Figure 1A). The prevalence rate was consistently higher in females than in males (2021: Females 83,968.21 vs. Males 75,884.21 per 100,000), a difference primarily driven by disease categories with a higher burden in females, such as mental disorders and digestive diseases. Trends varied significantly across age groups. Adolescents (15–19 years) experienced an increase in prevalence (AAPC = 0.02%, 95% CI: 0.02–0.03, *P* < 0.001), while children under 5 years showed the most pronounced decline (AAPC = −0.05%, 95% CI:−0.06 to−0.03). At the disease category level, the prevalence of cardiovascular diseases (AAPC = 0.45%) and diabetes and kidney diseases (AAPC = 0.40%) increased significantly (both *P* < 0.001); in contrast, digestive diseases (AAPC = −1.69%) and chronic respiratory diseases (AAPC = −1.07%) showed the most substantial declines (Table 1).

**Table 1:** The prevalence of level-1 and level-2 NCDs and their average annual percentage changes among children and adolescents globally from 1990 to 2021.

Location	1990–Number	1990–ASPR	2021–Number	2021–ASPR	AAPC (95%CI)	*P*
Global Level 1 NCD	1,817,492,089 (1,738,909,498, 1,892,091,221)	80,475.81 (76,993.03, 83,784.22)	2,115,137,790 (2,035,276,958, 2,191,054,557)	79,801.75 (76,751.23, 82,696.63)	−0.02 (-0.03,−0.02)	< 0.001
SDI						
High SDI	191,622,373 (183,567,089, 199,352,159)	75,172.82 (71,928.10, 78,295.50)	178,148,254 (171,025,575, 184,999,925)	75,099.59 (71,984.57, 78,104.28)	0.02 (0.00, 0.03)	0.024
High–middle SDI	291,667,562 (276,715,513, 306,213,687)	78,175.05 (74,047.83, 82,176.80)	235,469,530 (223,734,344, 246,846,159)	76,913.59 (72,975.69, 80,701.84)	−0.05 (-0.06,−0.04)	< 0.001
Middle SDI	617,357,849 (590,612,947, 644,299,139)	80,473.43 (76,940.77, 84,028.52)	599,576,503 (575,233,011, 623,108,231)	79,364.70 (76,068.91, 82,529.08)	−0.05 (-0.06,−0.04)	< 0.001
Low–middle SDI	480,900,166 (459,688,478, 501,122,755)	81,859.77 (78,323.16, 85,235.69)	617,469,583 (593,863,080, 639,462,618)	80,291.82 (77,179.75, 83,188.71)	−0.06 (-0.07,−0.05)	< 0.001
Low SDI	234,361,263 (225,999,791, 241,918,029)	84,741.58 (81,820.68, 87,384.93)	482,876,846 (466,996,458, 497,354,633)	82,900.47 (80,192.79, 85,369.15)	−0.07 (-0.09,−0.06)	< 0.001
Sex						
Male	890,402,961 (842,951,801, 936,169,284)	76,979.07 (72,879.19, 80,934.99)	1,036,634,045 (987,816,005, 1,084,561,227)	75,884.21 (72,275.20, 79,418.99)	−0.04 (-0.05,−0.04)	< 0.001
Female	927,089,128 (895,062,859, 956,776,216)	84,146.02 (81,230.84, 86,851.80)	1,078,503,745 (1,046,370,552, 1,107,752,395)	83,968.21 (81,430.46, 86,277.75)	−0.01 (-0.01, 0.00)	0.038
Years						
< 5 years	425,913,384 (400,179,910, 447,981,451)	68,702.59 (64,551.61, 72,262.31)	445,223,375 (422,582,611, 465,590,887)	67,645.48 (64,205.53, 70,740.04)	−0.05 (-0.06,−0.03)	< 0.001
5–9 years	469,994,478 (445,741,962, 494,237,082)	80,543.23 (76,387.06, 84,697.70)	543,870,987 (519,722,919, 567,243,428)	79,159.91 (75,645.18, 82,561.75)	−0.05 (-0.06,−0.04)	< 0.001
10–14 years	454,072,948 (437,906,615, 470,198,062)	84,765.29 (81,747.40, 87,775.49)	560,786,651 (541,753,660, 579,415,967)	84,121.87 (81,266.79, 86,916.40)	−0.03 (-0.04,−0.02)	< 0.001
15–19 years	467,511,279 (455,081,012, 479,674,626)	90,005.81 (87,612.72, 92,347.51)	565,256,777 (551,217,768, 578,804,274)	90,588.94 (88,339.03, 92,760.09)	0.02 (0.02, 0.03)	< 0.001
Level 2 NCDs						
Neoplasms	8,057,269 (5,639,395, 11,753,317)	355.85 (249.02, 519.05)	8,945,345 (6,403,355, 12,641,093)	334.51 (240.30, 471.18)	−0.24 (-0.28,−0.19)	< 0.001
Cardiovascular diseases	15,345,813 (11,957,507, 19,489,002)	678.64 (528.85, 861.67)	20,928,984 (15,938,130, 27,086,060)	774.33 (590.50, 1,000.82)	0.45 (0.40, 0.49)	< 0.001
Chronic respiratory diseases	140,408,064 (102,802,753, 193,333,402)	6,229.70 (4,560.49, 8,582.18)	115,413,707 (82,646,815, 160,785,035)	4,396.84 (3,148.16, 6,124.62)	−1.07 (-1.16,−0.98)	< 0.001
Digestive diseases	218,780,889 (196,787,749, 243,447,657)	9,651.59 (8,680.85, 10,739.94)	154,343,359 (136,915,012, 172,903,812)	5,701.35 (5,057.96, 6,386.18)	−1.69 (-1.77,−1.61)	< 0.001
Neurological disorders	450,049,535 (355,533,960, 556,048,963)	19,917.66 (15,736.35, 24,613.14)	552,286,120 (436,024,296, 682,923,672)	20,112.17 (15,870.97, 24,881.85)	0.18 (0.12, 0.25)	< 0.001
Mental disorders	206,639,135 (174,325,390, 241,958,970)	9,153.46 (7,720.54, 10,721.11)	252,256,612 (215,166,532, 293,739,165)	9,310.21 (7,929.17, 10,854.07)	0.18 (0.12, 0.25)	< 0.001
Musculoskeletal disorders	50,550,886 (38,744,112, 64,425,006)	2,220.87 (1,702.21, 2,831.59)	62,467,115 (48,725,089, 78,504,479)	2,262.25 (1,763.70, 2,844.65)	0.05 (0.03, 0.06)	< 0.001
Other non–communicable diseases	1,306,483,319 (1,147,438,071, 1,471,077,741)	57,831.83 (50,781.48, 65,131.53)	1,530,495,900 (1,376,036,721, 1,693,449,738)	58,013.95 (52,175.70, 64,146.86)	0.01 (0.00, 0.02)	0.019
Skin and subcutaneous diseases	544,058,132 (500,694,451, 594,901,736)	24,087.13 (22,165.88, 26,340.45)	683,101,377 (626,821,814, 749,406,084)	25,700.36 (23,579.52, 28,196.56)	0.19 (0.17, 0.21)	< 0.001
Sense organ diseases	94,778,289 (78,466,688, 115,426,107)	4,191.15 (3,469.94, 5,104.58)	118,903,938 (98,736,000, 144,571,007)	4,414.65 (3,668.07, 5,364.08)	0.17 (0.15, 0.18)	< 0.001
Substance use disorders	10,123,069 (7,609,154, 13,239,087)	438.56 (329.44, 573.79)	10,234,872 (7,627,581, 13,415,958)	368.58 (274.63, 483.20)	−0.61 (-0.65,−0.57)	< 0.001
Diabetes and kidney diseases	17,327,008 (14,411,881, 20,755,110)	758.47 (630.98, 908.45)	23,628,736 (19,638,012, 28,324,720)	857.16 (712.46, 1,027.46)	0.40 (0.37, 0.42)	< 0.001

During the same period, the global age-standardized incidence rate (ASIR) exhibited an increasing trend (AAPC = 0.05%, 95% CI: 0.03–0.07, *P* < 0.001), reaching 150,291.52 per 100,000 in 2021 (Table 2, Figure 1B). Trends varied across SDI regions: high SDI regions experienced an increase in incidence (AAPC = 0.09%), while low SDI regions experienced a decrease (AAPC = −0.13%). Mental disorders (AAPC = 0.90%) and diabetes and kidney diseases (AAPC = 1.07%) showed the largest increases in incidence (both *P* < 0.001). Conversely, neoplasms (AAPC = −0.39%) and substance use disorders (AAPC = −0.61%) decreased in incidence (Table 2).

**Table 2:** The incidence of level-1 and level-2 NCDs and their average annual percentage changes among children and adolescents globally from 1990 to 2021.

Location	1990–Number	1990–ASIR	2021–Number	2021–ASIR	AAPC (95%CI)	*P*
Global Level 1 NCD	3,328,982,042 (2,750,144,068, 4,065,970,586)	147,790.97 (121,961.34, 180,799.92)	3,977,853,077 (3,394,303,734, 4,670,723,137)	150,291.52 (128,290.68, 176,265.90)	0.05 (0.03, 0.07)	< 0.001
SDI						
High SDI	324,932,542 (271,565,265, 391,689,010)	128,235.04 (106,766.20, 155,070.48)	307,849,130 (260,101,430, 364,259,187)	130,179.60 (109,724.35, 154,267.57)	0.09 (0.05, 0.14)	< 0.001
High–middle SDI	496,907,806 (404,950,913, 616,283,938)	135,017.78 (109,636.65, 167,736.02)	425,418,416 (347,195,144, 525,483,027)	138,057.79 (112,803.03, 170,044.23)	0.08 (0.07, 0.10)	< 0.001
Middle SDI	1,085,133,904 (888,004,975, 1,340,988,879)	142,527.05 (116,390.13, 176,398.32)	1,105,736,129 (931,106,338, 1,324,760,254)	146,309.59 (123,302.44, 174,877.72)	0.08 (0.06, 0.11)	< 0.001
Low–middle SDI	937,731,448 (770,980,600, 1,149,127,932)	158,526.14 (130,480.43, 194,506.08)	1,170,114,686 (1,005,843,877, 1,359,390,481)	152,886.65 (131,411.16, 177,482.13)	−0.14 (-0.17,−0.11)	< 0.001
Low SDI	481,341,388 (406,799,523, 574,785,812)	172,556.97 (146,044.88, 206,311.65)	965,763,995 (839,709,714, 1,109,613,798)	165,412.66 (143,863.88, 190,015.43)	−0.13 (-0.15,−0.11)	< 0.001
Sex						
Male	1,637,813,626 (1,336,341,117, 2,022,252,828)	141,913.14 (115,671.11, 175,523.99)	1,970,266,226 (1,668,859,644, 2,327,635,166)	144,572.53 (122,529.81, 170,558.35)	0.07 (0.05, 0.10)	< 0.001
Female	1,691,168,417 (1,410,344,003, 2,046,030,132)	153,917.66 (128,208.76, 186,507.78)	2,007,586,851 (1,725,153,704, 2,339,926,121)	156,354.14 (134,384.99, 182,068.58)	0.05 (0.04, 0.05)	< 0.001
Years						
< 5 years	673,146,787 (579,146,260, 764,747,284)	108,582.93 (93,420.04, 123,358.69)	744,077,749 (662,922,448, 830,646,049)	113,052.23 (100,721.81, 126,205.07)	0.16 (0.14, 0.17)	< 0.001
5–9 years	1,196,335,036 (950,585,842, 1,422,837,748)	205,016.63 (162,902.45, 243,832.54)	1,383,767,810 (1,141,330,467, 1,601,517,946)	201,406.10 (166,119.57, 233,099.43)	−0.07 (-0.08,−0.05)	< 0.001
10–14 years	796,093,747 (619,203,012, 1,145,463,932)	148,612.95 (115,591.39, 213,832.57)	973,073,459 (794,465,695, 1,275,929,847)	145,967.74 (119,175.34, 191,398.29)	−0.08 (-0.15,−0.02)	0.015
15–19 years	663,406,473 (601,208,955, 732,921,622)	127,719.78 (115,745.44, 141,102.91)	876,934,058 (795,585,124, 962,629,295)	140,538.84 (127,501.73, 154,272.49)	0.30 (0.27, 0.34)	< 0.001
Level 2 NCDs						
Neoplasms	4,907,118 (3,250,462, 7,564,026)	216.75 (143.55, 333.66)	5,150,647 (3,487,429, 7,819,012)	193.13 (131.14, 291.72)	−0.39 (-0.42,−0.35)	< 0.001
Cardiovascular diseases	2,134,448 (1,489,491, 2,993,819)	94.50 (65.89, 132.63)	2,738,540 (1,824,083, 3,988,414)	102.25 (68.33, 148.59)	0.25 (0.22, 0.28)	< 0.001
Chronic respiratory diseases	30,439,927 (18,837,386, 47,879,534)	1,346.13 (832.14, 2,118.69)	25,514,849 (15,396,725, 40,353,487)	997.01 (603.92, 1,574.31)	−0.92 (-1.01,−0.83)	< 0.001
Digestive diseases	40,220,248 (33,484,888, 48,122,346)	1,763.91 (1,468.89, 2,110.02)	43,332,921 (35,746,117, 52,436,558)	1,598.79 (1,320.50, 1,932.23)	−2.73 (-2.85,−2.61)	< 0.001
Neurological disorders	177,442,117 (125,548,080, 238,632,952)	7,885.43 (5,580.46, 10,601.31)	216,329,232 (152,393,868, 291,534,098)	7,933.70 (5,589.94, 10,693.35)	0.02 (0.00, 0.03)	0.013
Mental disorders	58,658,984 (45,635,667, 72,581,819)	2,593.84 (2,018.30, 3,209.04)	85,593,014 (65,633,447, 106,537,813)	3,138.85 (2,408.77, 3,905.92)	0.90 (0.73, 1.06)	< 0.001
Musculoskeletal disorders	22,266,810 (15,877,870, 30,110,407)	983.34 (701.07, 1,329.93)	25,829,681 (18,534,098, 34,838,480)	939.88 (673.95, 1,268.51)	−0.16 (-0.16,−0.14)	< 0.001
Other non–communicable diseases	1,850,246,786 (1,303,553,167, 2,544,189,614)	82,314.76 (57,898.36, 113,427.59)	2,108,508,927 (1,583,154,196, 2,754,488,223)	79,825.07 (60,019.93, 104,018.98)	−0.13 (-0.15,−0.12)	< 0.001
Skin and subcutaneous diseases	1,135,942,981 (1,018,353,999, 1,275,814,516)	50,299.39 (45,085.80, 56,497.56)	1,457,316,256 (1,310,090,320, 1,637,242,750)	55,287.04 (49,719.77, 62,100.30)	0.29 (0.25, 0.32)	< 0.001
Sense organ diseases	209,493 (98,531, 471,448)	9.18 (4.32, 20.65)	214,543 (101,374, 479,940)	8.85 (4.18, 19.80)	−0.15 (-0.20,−0.08)	< 0.001
Substance use disorders	5,096,939 (3,692,224, 7,006,280)	221.62 (160.33, 304.82)	5,081,950 (3,715,101, 6,970,709)	183.09 (133.79, 251.19)	−0.62 (-0.67,−0.58)	< 0.001
Diabetes and kidney diseases	1,416,192 (1,115,809, 1,759,449)	62.12 (48.93, 77.21)	2,242,518 (1,783,474, 2,764,683)	83.86 (66.87, 103.18)	1.07 (1.00, 1.14)	< 0.001

The global age-standardized mortality rate (ASMR) decreased substantially (AAPC = −2.20%, 95% CI:−2.26 to−2.13, *P* < 0.001), falling to 37.31 per 100,000 in 2021 (Supplementary Table 1, Supplementary Figure S1A). Mortality rates decreased for all Level 2 NCD categories, with the most significant declines observed for cardiovascular diseases (AAPC = −2.68%) and chronic respiratory diseases (AAPC = −3.28%). Although the high-middle SDI region experienced the fastest decline (AAPC = −4.04%), the low SDI region still had the highest mortality rate in 2021 (ASMR = 60.94) (Supplementary Table 1, Supplementary Figure S1A). The global age-standardized disability-adjusted life year rate (ASDR) also decreased significantly (AAPC = −1.30%, 95% CI:−1.33 to−1.27, *P* < 0.001), reaching 6,444.15 per 100,000 in 2021 (Supplementary Table 2, Supplementary Figure S1B). A key finding was the significant increase in the ASDR for mental disorders (AAPC = 0.54%, 95% CI: 0.44–0.65), which contrasted with the general declining trend in mortality. Meanwhile, the ASDR burden for cardiovascular diseases (AAPC = −2.43%) and digestive diseases (AAPC = −2.45%) decreased substantially (both *P* < 0.001) (Supplementary Table 2). The high SDI region showed a slower decline in ASDR (AAPC = −0.71%), while the low SDI region continued to bear the heaviest disease burden in 2021 (ASDR = 8,501.66) (Supplementary Table 2, Supplementary Figure S1B).

### 3.2 Country-level variations

Analysis across 204 countries and territories revealed substantial disparities in the burden of non-communicable diseases (NCDs) among children and adolescents. These disparities exhibited distinct patterns across different metrics (prevalence, incidence, mortality, and DALYs), which can be categorized into several thematic findings.

**Figure 2:**
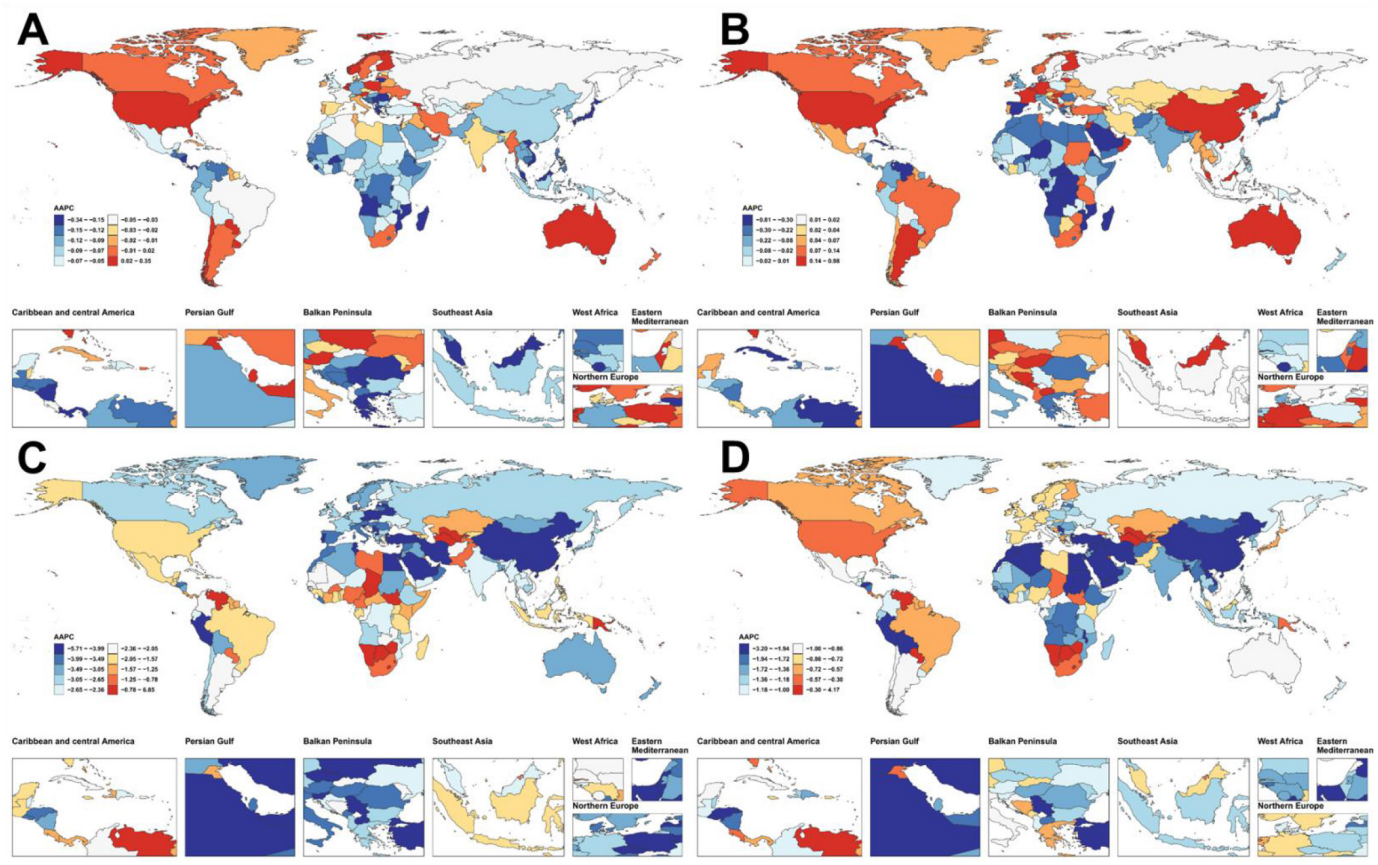
Average Annual Percentage Change (AAPC) of global age-standardized rates of NCDs among children and adolescents from 1990 to 2021. **(A)** AAPC of age-standardized prevalence rate (ASPR). **(B)** AAPC of age-standardized incidence rate (ASIR). **(C)** AAPC of age-standardized mortality rate (ASMR). **(D)** AAPC of age-standardized DALY rate (ASDR).

Some nations demonstrated remarkable progress between 1990 and 2021, despite varying starting points. In reducing mortality, Iran (AAPC:−5.65%) and Saudi Arabia (AAPC:−5.48%) showed the most rapid declines, likely attributable to strengthened healthcare systems and effective public health interventions (Supplementary Table 5, Figure 2C). Similarly, for reducing the overall disease burden (DALYs), Egypt (AAPC:−3.17%) and Iran (AAPC:−3.00%) were among the leading countries (Supplementary Table 6, Figure 2D). Furthermore, Madagascar showed notable performance in reducing both prevalence (AAPC:−0.33%) and incidence (AAPC:−0.80%), while Niger achieved the fastest decline in incidence (AAPC:−0.78%) (Supplementary Tables 3, 4, Figures 2A, B). These cases provide examples of successful control strategies implemented across different developmental contexts.

In contrast to these successes, several high-income countries exhibited concerning upward trends. Australia (AAPC: +0.35%) and the United States (AAPC: +0.10%) had the largest increases in age-standardized prevalence rate, while Finland (AAPC: +0.58%) and Belgium (AAPC: +0.40%) experienced significant rises in age-standardized incidence rate (Supplementary Tables 3, 4, Figures 2A, B). These trends may reflect the combined effects of multiple factors, such as the obesity epidemic, expanded diagnostic practices, or mental health crises, which will be explored further in the Discussion.

**Figure 3:**
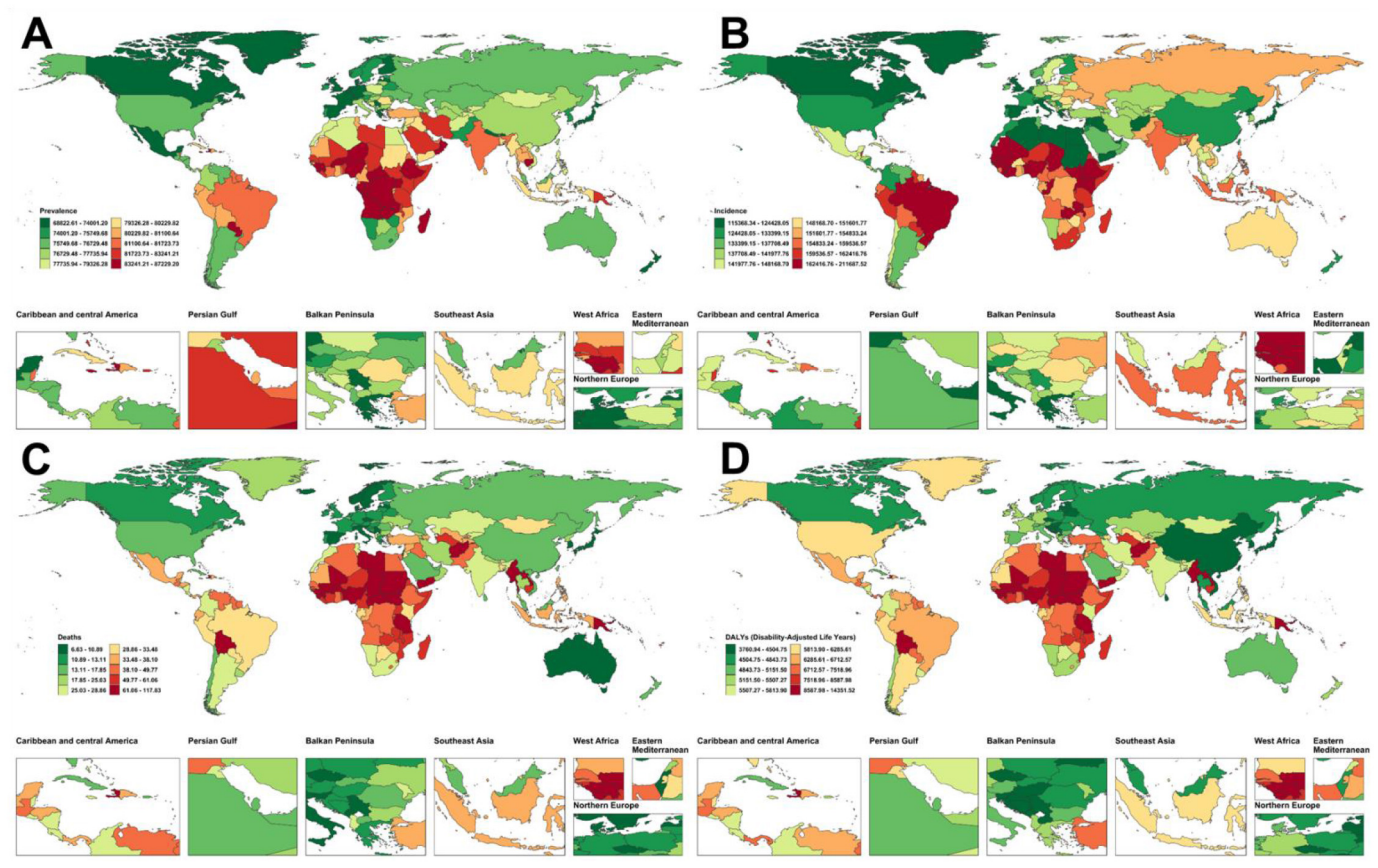
Global distribution of age-standardized prevalence rate (ASPR) and incidence rate (ASIR) of NCDs among children and adolescents in 2021. **(A)** Age-standardized prevalence rate (ASPR). **(B)** Age-standardized incidence rate (ASIR). **(C)** Age-standardized mortality rate (ASMR). **(D)** Age-standardized DALY rate (ASDR).

Despite global improvements, a heavy disease burden remained highly concentrated in specific regions in 2021. Nigeria and Madagascar had the highest age-standardized prevalence rates, while Ethiopia and Niger carried the heaviest burden in terms of incidence (Supplementary Tables 3, 4, Figures 3A, B). Regarding mortality and DALYs, South Sudan, Yemen, Afghanistan, and Haiti, despite substantial declines since 1990, still had among the highest burdens globally in 2021 (Supplementary Tables 5, 6, Figures 3C, D).

The analysis also included some sparsely populated countries and territories, whose extreme values warrant cautious interpretation. For instance, Niue and Tokelau showed exceptionally high increases in both mortality (AAPCs of 6.78% and 12.23%, respectively) and DALYs (AAPCs of 4.13% and 6.12%, respectively) (Supplementary Tables 5, 6, Figures 2C, D). Due to their extremely small population sizes, these estimates may be unstable and should be interpreted with caution.

### 3.3 Correlation between ASRs and SDI

**Figure 4:**
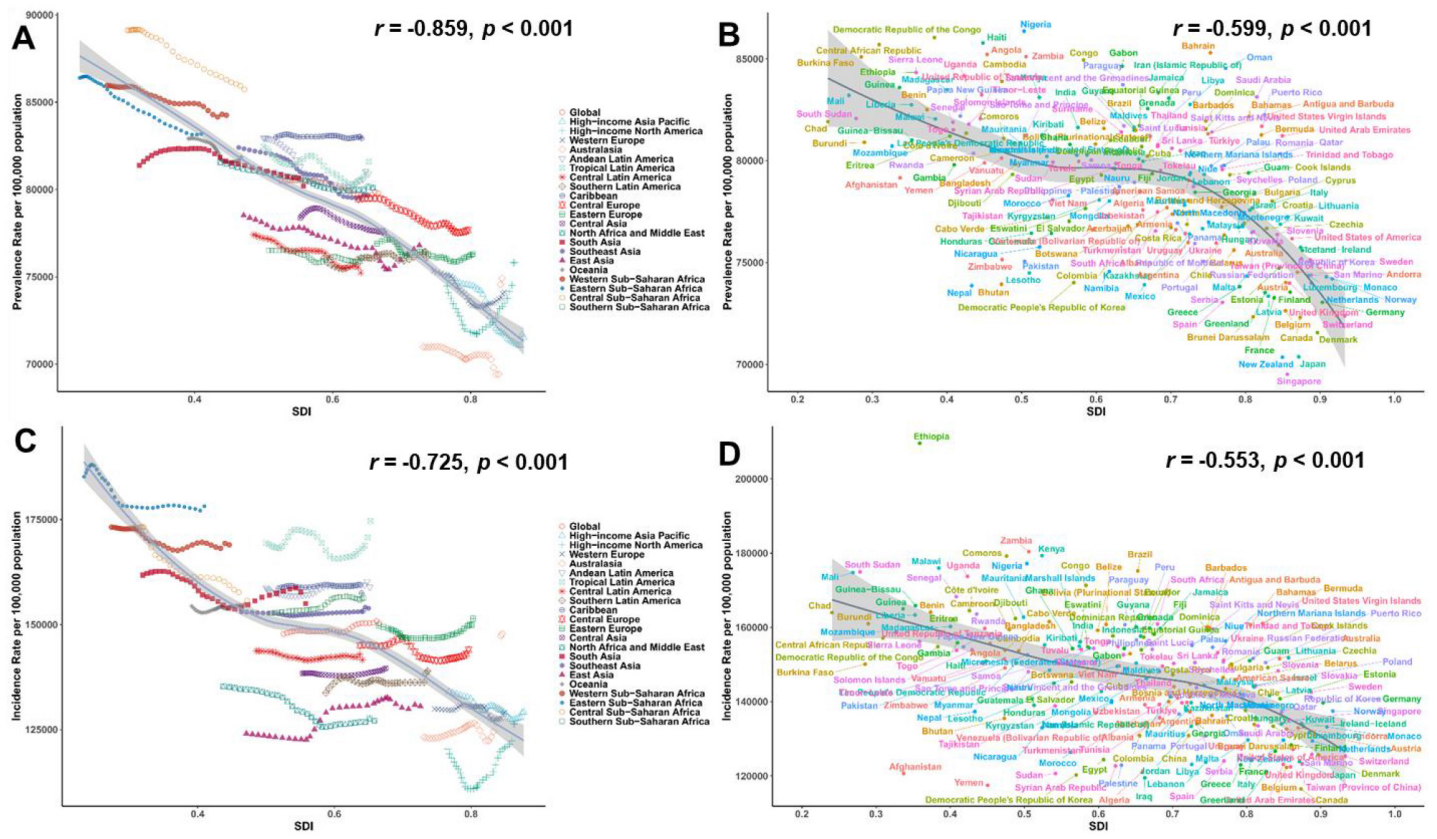
Spearman correlation analysis of age-standardized rates of NCDs among children and adolescents with the Socio-demographic Index (SDI) for 21 global regions and 204 countries from 1990 to 2021. **(A)** Age-standardized prevalence rate (ASPR) and SDI for 21 global regions. **(B)** Age-standardized incidence rate (ASIR) and SDI for 21 global regions. **(C)** ASPR and SDI for 204 countries. **(D)** ASIR and SDI for 204 countries.

A significant negative correlation was observed globally between the age-standardized rates (ASRs) of non-communicable diseases (NCDs) in children and adolescents and the Socio-demographic Index (SDI). The analysis revealed strong negative correlations between SDI and both the age-standardized prevalence rate (ASPR) and incidence rate (ASIR) (ASPR: *r* = −0.859, *p* < 0.001; ASIR: *r* = −0.725, *p* < 0.001) (Figures 4A, C). This association was even stronger for mortality and disability-adjusted life year rates (ASMR: *r* = −0.880; ASDR: *r* = −0.826; both *p* < 0.001) (Supplementary Figures S2A, S2C). Overall, high-SDI countries (e.g., Japan, South Korea) exhibited substantially lower burdens across all disease metrics compared to low-SDI countries (e.g., Central African Republic, Afghanistan), with the heaviest burden concentrated in Sub-Saharan Africa (Supplementary Tables 3–6).

Despite this clear overall correlation, data from several countries significantly deviated from the regression line, revealing unique risk or protective factors that operate beyond development levels. High-Burden Outliers among High-SDI Countries: Some high-SDI countries, notably the United States and Brazil, demonstrated significantly higher prevalence, incidence, and DALY burdens than expected for their SDI levels (Figures 4B, D, Supplementary Figures S2B, S2D). For instance, the ASDR in the United States (5,967.73 per 100,000) was considerably higher than in countries of similar developmental standing, potentially linked to domestic challenges such as the obesity epidemic, mental health crises, or inequalities in healthcare access. Low-Burden Outliers among Low-SDI Countries: Conversely, several low-SDI countries exhibited a much lower disease burden than predicted, suggesting the potential presence of effective localized public health strategies. Bhutan, with its ASPR of 73,939.04 per 100,000 ranking among the lower global levels, serves as a successful example (Figure 4B). These countries provide valuable lessons for effectively controlling NCDs within resource-constrained settings. These findings collectively confirm that higher SDI levels are generally associated with a lower NCD burden. However, the significant deviations of specific countries from the overall trend underscore the critical importance of considering unique national contexts, in addition to development levels, when formulating intervention strategies.

### 3.4 Joinpoint regression analysis

**Figure 5:**
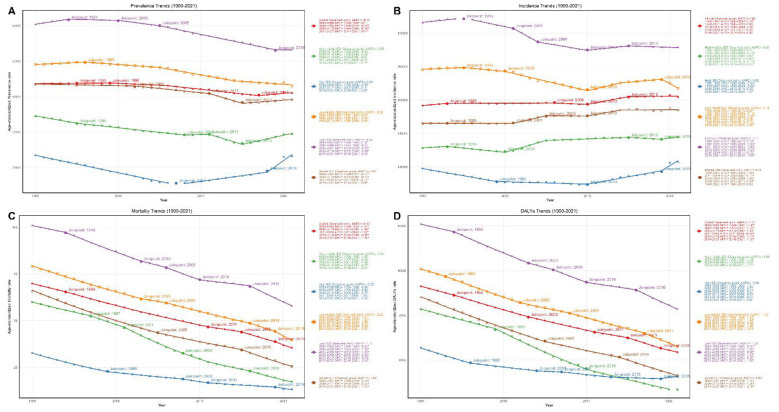
Joinpoint regression analysis of age-standardized rates of NCDs among children and adolescents in five global SDI regions from 1990 to 2021. **(A)** Age-standardized prevalence rate (ASPR). **(B)** Age-standardized incidence rate (ASIR). **(C)** Age-standardized mortality rate (ASMR); **(D)** Age-standardized DALY rate (ASDR).

Joinpoint regression analysis revealed significant changes in the trends of the global burden of non-communicable diseases (NCDs) among children and adolescents from 1990 to 2021, alongside a growing divergence across different Socio-demographic Index (SDI) regions. Although the global age-standardized prevalence rate (ASPR) showed a slight overall decrease (AAPC = −0.02%), a crucial trend reversal occurred between 2015 and 2018, shifting from decline to increase (Figure 5A). This reversal was most pronounced in the high-SDI region, which experienced the fastest increase during 2019–2021 (APC = +0.55%). Concurrently, the global age-standardized incidence rate (ASIR) exhibited a modest overall increase (AAPC = 0.08%), but its trajectory demonstrated a fundamental divergence across SDI regions (Figure 5B). The high and high-middle SDI regions experienced persistent increases in incidence, whereas the low-middle and low SDI regions showed declining trends. This polarization peaked in the 2019–2021 period, where the high-SDI region saw a sharp rise in incidence (APC = +1.17%), contrasting with a rapid decline in the low-middle SDI region (APC = −0.87%)—a difference exceeding 2 percentage points in their annual change rates.

In stark contrast to the complex trends for prevalence and incidence, the global age-standardized mortality rate (ASMR) demonstrated a continuous and substantial decline (AAPC = −4.77%), with an accelerated rate of decrease in recent years, averaging−4.40% annually during 2019–2021 (Figure 5C). All SDI regions entered a phase of accelerated decline after 2015, with the low-middle SDI region showing the fastest decline during 2019–2021 (APC = −5.55%). However, cumulative progress remained associated with SDI levels; the overall reduction in the high-SDI region (AAPC = −2.92%) was still more than 1.6 times greater than that in the low-SDI region (AAPC = −1.77%), indicating the persistence of absolute inequalities in disease burden. Similarly, the global age-standardized disability-adjusted life year (DALY) rate (ASDR) showed a declining trend (global AAPC = −1.22%), but with notable differences in regional efficiency (Figure 5D). The high-middle SDI region achieved the most significant reduction in burden (AAPC = −1.86%), while the high-SDI region showed the slowest improvement (AAPC = −0.69%). Starting around 2014, several middle and low SDI regions entered a phase of accelerated decline, with their recent annual rates of reduction (e.g., APC = −2.13% for the low-middle SDI region during 2017–2021) surpassing those of the high-SDI region, suggesting an improvement in the efficiency of their disease prevention and control efforts.

### 3.5 Frontier analysis

**Figure 6:**
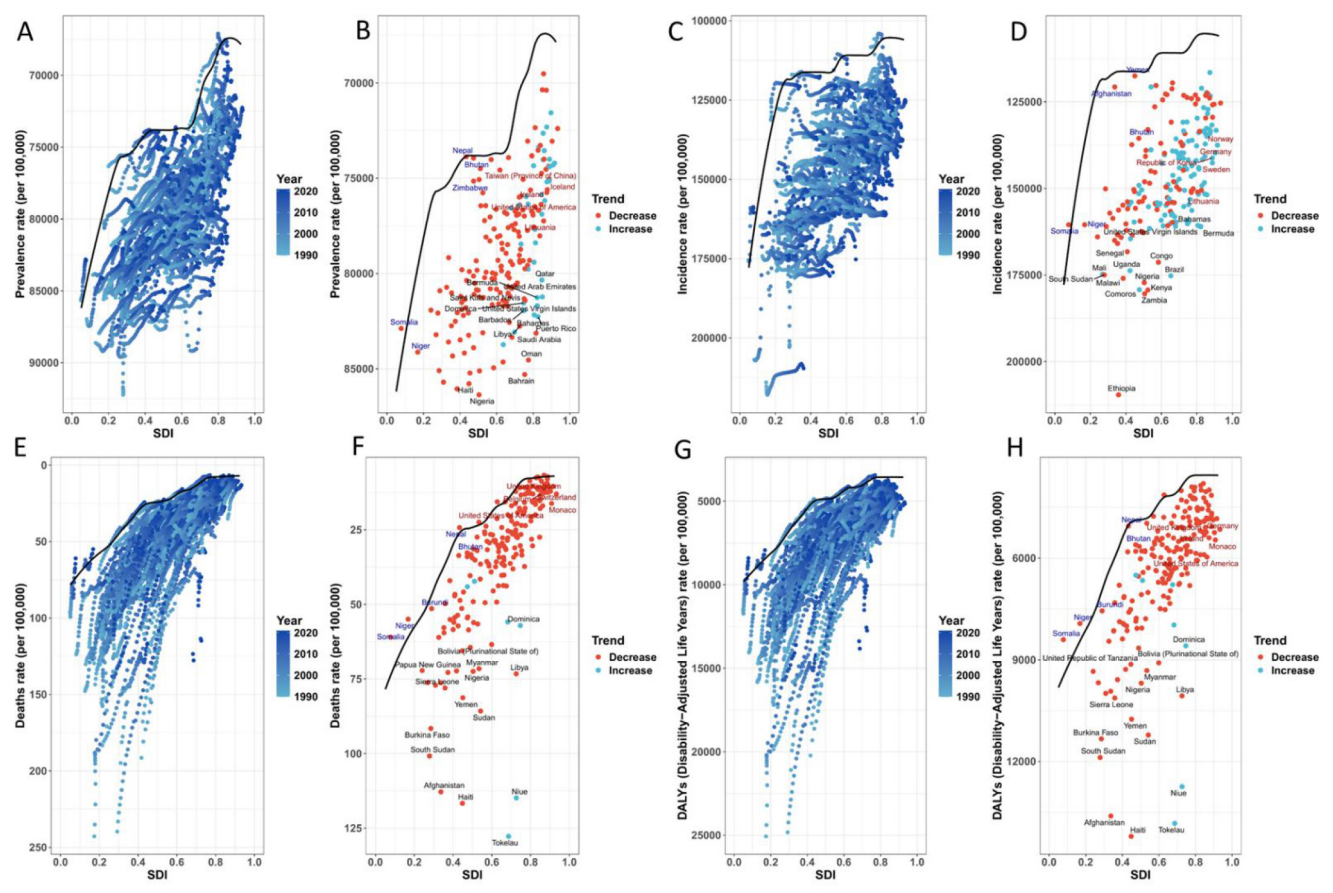
Frontier analysis of the relationship between age-standardized rates of NCDs and the Socio-demographic Index (SDI) among children and adolescents in 204 countries and regions. In panels **(A)**, **(C)**, **(E)**, and **(G)**, the color gradient from light green (1990) to dark green (2021) indicates changes over time. In panels **(B)**, **(D)**, **(F)**, and **(H)**, each point represents a specific country or region in 2021, with the frontier line shown in black. Blue indicates lower SDI and minimal deviation from the frontier line, while red indicates higher SDI and maximum deviation from the frontier line. The direction of change in the age-standardized rate (ASR) from 1990 to 2021 is indicated by the color of the points, with orange representing a decrease and green representing an increase. The frontier line represents the lowest age-standardized rate (ASIR or ASDR) observed in 2021 for each given SDI level; it indicates the theoretically achievable minimum disease burden under current development conditions. Panels **(A)** and **(B)** show age-standardized prevalence rates (ASPR), Panels **(C)** and **(D)** show age-standardized incidence rates (ASIR), Panels **(E)** and **(F)** show age-standardized mortality rates (ASMR), and Panels **(G)** and **(H)** show age-standardized disability-adjusted life-years (DALY) rates (ASDR).

Frontier analysis based on the 2021 GBD data revealed substantial disparities between the observed age-standardized rates of non-communicable diseases (NCDs) in various countries and the theoretical optimal health levels achievable given their Socio-demographic Index (SDI) (Figures 6A–H). Regarding prevalence and incidence, the performance of countries across different SDI levels exhibited a complex pattern. Among high-SDI countries (SDI > 0.8), Saudi Arabia, Bahrain, and Oman showed substantial gaps between their actual prevalence rates and the theoretical optimum (efficiency difference > 15,000), indicating significant room for efficiency improvements in their health systems (Figures 6A, B). Notably, high-income nations like the United States (efficiency difference = 8,656) also demonstrated considerable potential for enhancement. Among low- and middle-income countries, Ethiopia (efficiency difference = 93,409.7) and Brazil (64,385.7) exhibited the most pronounced gaps in incidence, whereas the observed values for countries like Yemen (1,279.9) and Niger (1,653.1) were close to the theoretical frontier corresponding to their SDI levels (Figures 6C, D). This finding suggests that under extreme resource constraints, the “optimal level” defined by the frontier model may reflect a health state attainable within limited conditions rather than an ideal one. Concerning age-standardized mortality and disability-adjusted life year (DALY) rates, Tokelau faced the most severe efficiency gaps (mortality efficiency difference = 112.98; DALY efficiency difference = 9,730.8), with its actual values being 8.7 times and 3.4 times the theoretical expectations, respectively, highlighting the severe challenges confronting its health system (Figures 6E, G). Similar phenomena were observed in low-income countries like Haiti and Afghanistan, indicating that economic development level is not the sole determinant of health performance. The analysis also identified exemplary cases. Low-SDI countries such as Somalia and Niger (efficiency difference = 0) achieved optimal outcomes commensurate with their resource constraints (Figures 6F, H). Among high-SDI countries, Switzerland (5.8) and the United Kingdom (5.6) were very close to the efficiency frontier, setting a benchmark for peer nations (Figures 6E, G).

### 3.6 Health inequality analysis

**Figure 7:**
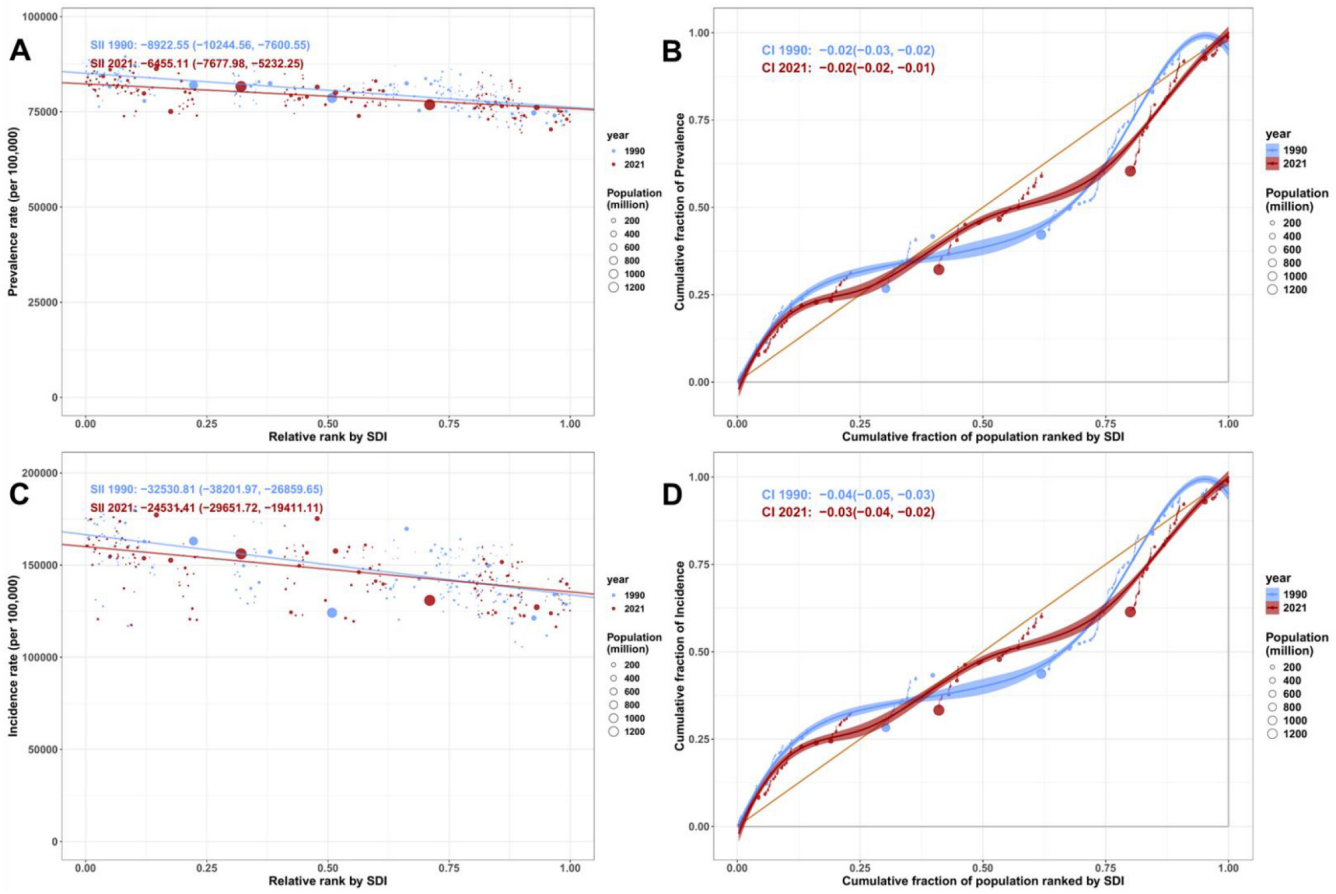
Health inequality regression and concentration curves of age-standardized prevalence and incidence rates of NCDs among children and adolescents globally in 1990 and 2021. Panels **(A)** and **(C)** show the slope index of inequality, depicting the relationship between the Socio-demographic Index (SDI) and age-standardized rates, with points representing countries and regions weighted by population size. Panels **(B)** and **(D)** show the concentration index, quantifying relative inequality by integrating the area under the Lorenz curve, aligning the distribution of age-standardized prevalence rates (ASPR) and age-standardized incidence rates (ASIR) with the population distribution stratified by SDI. Blue represents data from 1990, and red represents data from 2021.

Between 1990 and 2021, socioeconomic inequalities in the global burden of non-communicable diseases (NCDs) among children and adolescents showed improvement, yet the burden remained disproportionately concentrated among disadvantaged populations. For the age-standardized prevalence rate (ASPR), the Slope Index of Inequality (SII) improved from−8,922.55 in 1990 to−6,455.11 in 2021. However, the persistent negative value indicates that the prevalence burden continued to be disproportionately shouldered by populations with low SDI. The Concentration Index (CI) remained stable during this period (CII = −0.02) (Figures 7A, B).

Inequality in incidence was more pronounced. The SII for incidence improved substantially from−32,530.81 to−24,531.41, and the CI decreased from−0.04 to−0.03. Despite this improvement, the incidence burden among low-income populations remained excessively high (Figures 7C, D). Analysis of mortality revealed a critical finding: absolute inequality (SII) improved significantly by 44.5%, declining from−83.62 to−46.43. In contrast, relative inequality (CI) worsened from−0.25 to−0.28. This highlights that although mortality rates declined across all groups, the burden of deaths became increasingly concentrated, relative to the population distribution, among lower-income groups (Supplementary Figure S4A, Supplementary Figure S4B). For the age-standardized disability-adjusted life year (DALY) rate, both absolute inequality (SII) and relative inequality (CI) showed marked improvement. The SII improved from−7,035.80 to−3,370.99, and the CI improved from−0.16 to−0.11. Nevertheless, the disease burden continued to be heaviest among vulnerable populations (Supplementary Figure S4C, Supplementary Figure S4D).

### 3.7 Age-period-cohort analysis

**Figure 8:**
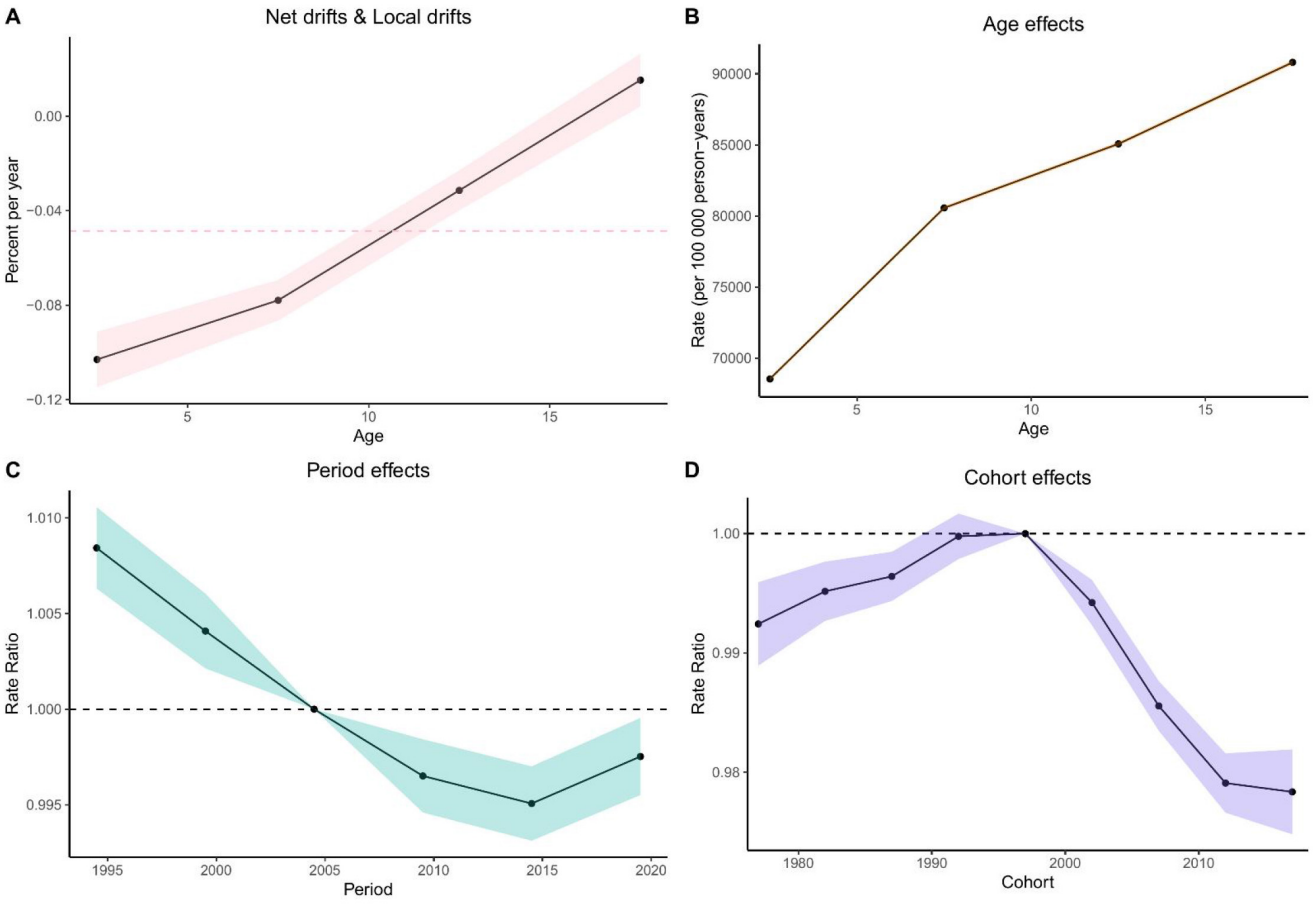
Age-period-cohort effect analysis of age-standardized prevalence rates of NCDs among children and adolescents globally. **(A)** Drift of ASPR; **(B)** Age effect of ASPR; **(C)** Period effect of ASPR; **(D)** Cohort effect of ASPR.

The Age-Period-Cohort analysis revealed distinct patterns in the burden of non-communicable diseases (NCDs) among children and adolescents across different ages, time periods, and birth cohorts. For the Age-Standardized Prevalence Rate (ASPR), the age effect showed a significant increase from 68,546.28 per 100,000 in the 2.5-year age group to 90,798.78 per 100,000 in the 17.5-year age group (*p* < 0.05). The period effect indicated a consistent decline in risk after 2004 (RR = 0.998 for 2019.5). Cohort analysis demonstrated a significantly lower risk for the 2017 birth cohort compared to the 1997 baseline (RR = 0.978, 95% CI: 0.975–0.982), translating to a mere 2.2% risk reduction over two decades, indicating a relatively limited pace of improvement (Figure 8, Supplementary Table 8). The Age-Standardized Incidence Rate (ASIR) exhibited a unique age pattern, peaking in the 7.5-year age group (204,300 per 100,000) before declining to 136,844 per 100,000 in the 17.5-year age group. Risks associated with recent birth cohorts (2017 RR = 0.99) and periods (2019.5 RR = 1.03) remained relatively stable. However, an upward trend in incidence was observed among adolescents (12.5–17.5 years), with local drifts ranging from 0.07% to 0.22% (Supplementary Figure S5). Analysis of the Age-Standardized Mortality Rate (ASMR) revealed marked age variations: the rate was highest in the 2.5-year age group (173.56 per 100,000), dropped sharply to 17.74 per 100,000 by the 7.5-year age group, and then rebounded to 24.14 per 100,000 in the 17.5-year age group. A significant cohort effect was observed, with the 2017 birth cohort experiencing a 39% reduction in risk compared to 1997 (RR = 0.61, 95% CI: 0.59–0.63). The period RR decreased from 1.26 in 1994.5 to 0.82 in 2019.5, with annual mortality reductions across age groups ranging from 1.04% to 2.25% (Supplementary Figure S6).

The ASDR displayed a U-shaped age curve: 17,203.75 in the 2.5-year age group, decreasing to 3,967.01 in the 7.5-year age group, and rising again to 7,204.63 in the 17.5-year age group. The DALY risk for the 2017 birth cohort was 38% lower than the baseline (RR = 0.62). Net drift indicated that the global DALY rate declined by an average of 0.94% per year, with the most substantial reduction observed in the 2.5-year age group (-1.95%), while improvement in the 17.5-year age group was limited (-0.11%) (Supplementary Figure S7). Collectively, these results indicate that although the disease burden in infancy and early childhood has improved significantly, the prevention and control of NCDs during adolescence remain challenging. Furthermore, viewed through the lens of cohort trends, the overall rate of risk improvement may be insufficient to rapidly achieve related global health targets.

### 3.8 Bayesian age-period-cohort analysis

**Figure 9:**
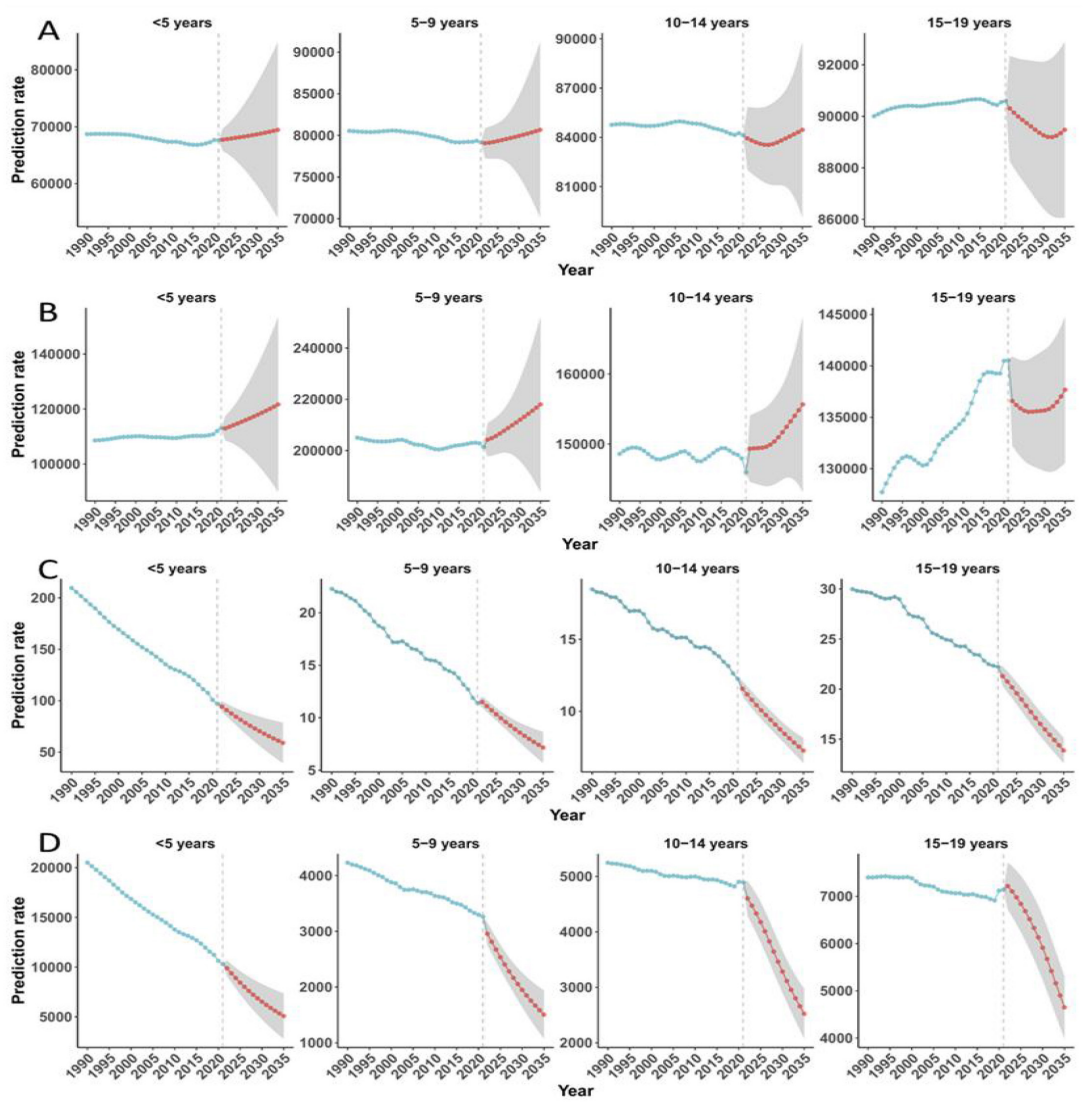
Bayesian Age-Period-Cohort (BAPC) predictions of age-standardized rates of NCDs among children and adolescents globally for different age groups from 1990 to 2035. **(A)** Age-standardized prevalence rate (ASPR). **(B)** Age-standardized incidence rate (ASIR). **(C)** Age-standardized mortality rates (ASMR), **(D)** Age-standardized disability-adjusted life-years (DALY) rates (ASDR).

Projections of the global burden of NCDs among individuals under 19 years from 2021 to 2035, based on the Bayesian Age-Period-Cohort (BAPC) model, reveal significant differences in future trends across age groups and metrics. For prevalence and incidence, the forecasts indicate a critical divergence in patterns. Compared to 2021, by 2035, the age-standardized prevalence rate (ASPR) and incidence rate (ASIR) are projected to increase in the under-5, 5–9, and 10–14 age groups. The most notable increase in incidence is anticipated in the 5–9 years age group, rising from 201,406.11 to 217,997.81 per 100,000. In contrast, the age-standardized prevalence and incidence rates for the 15–19 years adolescent group are projected to decline, with incidence decreasing from 140,538.82 to 137,676.08 per 100,000 (Figures 9A, B, Supplementary Table 9). This opposing trend—increasing in younger groups but decreasing in adolescents—signals an important shift in the age distribution of the disease burden.

In contrast, the future trends for mortality and disability-adjusted life years (DALYs) show consistent and positive improvements. The age-standardized mortality rate (ASMR) and age-standardized DALY rate (ASDR) are projected to decline significantly across all age groups. For instance, in the under-5 years age group, the ASMR is projected to decrease substantially from 97.14 (95% UI: 82.34–113.93) to 58.98 (95% UI: 46.84–73.44) per 100,000, while its ASDR is projected to drop from 10,314.57 (95% UI: 8,823.13–12,004.12) to 5,080.96 (95% UI: 4,083.46–6,299.48) per 100,000. Similar significant declining trends are observed in other age groups (Figures 9C, D, Supplementary Table 9). These projection results highlight the key population groups that require focused attention in future disease prevention and control efforts.

## 4 Discussion

Based on the Global Burden of Disease data from 1990 to 2021, this study identifies three core features of the NCD burden among children and adolescents: First, the disease pattern exhibits an “incidence-mortality decoupling” and a “development level paradox.” While the global age-standardized incidence rate (ASIR) increased by 0.05%, the mortality rate decreased substantially by 2.20%. Lower SDI regions bear the heaviest burden yet show a declining incidence trend, whereas higher SDI regions face rising prevalence and incidence. Second, a structural shift in the disease spectrum is occurring: the burden of metabolic diseases such as cardiovascular diseases and diabetes continues to rise, with mental disorders becoming the fastest-growing category. In contrast, traditional digestive and respiratory diseases have declined significantly. This shift is particularly pronounced among adolescents, whose prevalence increased against the overall trend (AAPC = +0.02%). Third, health inequalities have evolved in complex ways: although absolute gaps have narrowed, relative inequality in mortality has intensified. Frontier analysis further reveals significant deviations in health system efficiency among some high-SDI countries, while certain low-SDI countries have achieved near-optimal outcomes through efficient resource allocation. These findings collectively indicate that future prevention and control strategies must shift from a “one-size-fits-all” approach to “precision stratification,” focusing on three major challenges: addressing adolescent health lag, the rising burden of mental disorders, and the spread of metabolic diseases.

### 4.1 Comparison with existing evidence

The core findings of this study—the “decoupling” of rising incidence and falling mortality, and the “development level paradox” where lower-SDI regions show improving trends despite higher burden, while higher-SDI regions face worsening trends—provide a new dimension for understanding the global epidemiological transition of NCDs. This finding partially aligns with the negative correlation between NCD burden and SDI reported by Yu et al. (27) but reveals a critical dynamic evolution of this relationship. Specifically, while previous studies have largely focused on the macro-level trend of declining NCD mortality with societal development (28, 29), our analysis shows that, alongside improving mortality, the incidence rate of new cases continues to rise globally, particularly in high and middle-high SDI regions. This “decoupling” suggests that while medical advancements effectively prolong survival and reduce case fatality, they have not sufficiently curbed the emergence of new cases. This indicates that future disease burden pressure will stem primarily from a large prevalent population base rather than lethality. Consequently, global NCD prevention and control strategies for children and adolescents must pivot from a primary focus on reducing mortality to a dual task of preventing new cases and managing existing ones.

Regarding the evolution of the disease spectrum, our findings align with globally observed shifts in risk factors but provide more precise localization through detailed age and regional stratification. Mental disorders and diabetes and kidney diseases have become the categories with the fastest-growing incidence rates, consistent with macro-trends such as the global childhood obesity epidemic, dietary changes, and increasing psychosocial stress (30–32). However, this study further pinpoints adolescence as the “epicenter” of this crisis. Both joinpoint analysis and the age-period-cohort model confirm that adolescents aged 15–19 are the key demographic driving the increase in prevalence and the rise in incidence for specific diseases. This finding shifts the previous broad discussion of “childhood” risk to a precise focus on “adolescence,” a critical life stage fraught with unique physiological, psychological, and social transitions. Concurrently, the significant decline in traditional digestive and chronic respiratory disease burdens reflects public health achievements in environmental sanitation, nutritional improvements, and basic medical interventions, particularly in low-SDI regions, where these successes likely contributed to the observed decline in incidence. This shifting disease spectrum necessitates a strategic reorientation of NCD prevention and control priorities for children and adolescents.

### 4.2 Mechanisms and interpretation

The observed trends result from multiple interacting mechanisms. (a) Mechanistically, the substantial decline in mortality is primarily attributable to advancements in medical technology and the widespread implementation of public health interventions. The rise in incidence, however, stems from different factors across development contexts: in high-SDI countries, it is largely driven by the proliferation of lifestyle risk factors such as obesity, sedentary behavior, and unhealthy diets (33–35); in low-SDI countries, it may be associated with improved diagnostic capabilities leading to higher case detection rates, coupled with accumulating risk factors during urbanization (36). The sharp increase in the burden of mental disorders reflects a combination of evolving diagnostic criteria, increased societal awareness, and a genuine rise in psychosocial pressures, such as academic stress and the influence of social media (37). (b) In comparison with the literature, our frontier analysis results resonate with the view that “health system efficiency is not solely determined by economic level” (38). By examining contrasting cases like the United States and Bhutan, we provide empirical support for this theory. (c) The policy implication is the need to move beyond simplistic equating of development with health. High-SDI countries must focus on creating healthy public policy environments, such as taxing sugar-sweetened beverages. In contrast, low-SDI countries should invest in enhancing the efficiency and accessibility of primary healthcare systems. It is also important to note that part of the observed increase in mental disorders and autism spectrum disorders in high-SDI regions may reflect improvements in screening practices, broader diagnostic criteria, increased awareness among clinicians and families, and reduced social stigma toward mental health conditions over the past three decades. Therefore, the observed rise likely represents a combination of true epidemiological changes and improved case detection.

### 4.3 Policy and practical implications

To achieve the WHO Global NCD Action Plan (https://iris.who.int/server/api/core/bitstreams/15f51d0a-bfbd-43ad-a637-73778feb57e3/content) and Sustainable Development Goal (SDG) 3.4 (https://unstats.un.org/sdgs/metadata/files/Metadata-03-04-01.pdf), our findings advocate for a set of precise, stratified intervention strategies. For High-SDI Countries: The policy focus must shift from treatment to fundamental prevention. This includes implementing robust regulations to restrict the marketing of unhealthy foods and beverages to children, and fully integrating mental health screening and support services into the school health system. For Low-SDI Countries: The priority lies in consolidating gains and guarding against new risks. Lessons should be drawn from “super-efficient” nations (e.g., Bhutan, Niger) by training and deploying community health workers to expand the coverage of essential NCD services. Concurrently, vigilance is needed against the emerging risk of metabolic diseases associated with the nutrition transition. Focus on Key Population—Adolescents: Adolescent health must be established as an independent priority. Initiatives should include promoting Health-Promoting Schools, providing adolescent-friendly health services, and leveraging digital platforms for targeted health education. Addressing Inequalities: Pro-poor policies are essential to tackle the persistent and, in some cases, worsening relative inequalities identified in our health inequality analysis. Measures such as conditional cash transfers and targeted screening align closely with the WHO-recommended “best buys” for NCDs (39). Supporting this approach, a study published in JAMA Network Open in April 2025 indicated that combined diet and exercise interventions, or behavioral interventions alone, can effectively reduce abdominal obesity in children. The researchers called for coordinated action from governments, the WHO, and other stakeholders to promote comprehensive intervention strategies (40).

### 4.4 Strengths and limitations

The primary strength of this study lies in its utilization of the GBD 2021 database, which offers global comparability, long-term continuity, and comprehensiveness. The application of multiple statistical methods—including joinpoint regression, age-period-cohort modeling, and frontier analysis—enabled a multidimensional revelation of the complex landscape of the NCD burden. While this study leverages the comprehensive GBD 2021 database and employs multiple analytical methods to provide a multidimensional view of the global burden of NCDs among children and adolescents, several limitations warrant consideration. First, although the APC analysis using the intrinsic estimator (IE) method effectively addresses the identification problem, alternative approaches—such as constrained generalized linear models or Bayesian APC frameworks—may yield slightly different decompositions of age, period, and cohort effects. Therefore, the estimated APC effects should be interpreted with caution, as they may depend on the modeling assumptions inherent to the chosen method. Second, in the frontier analysis, setting the lowest observed burden at each SDI level as the “achievable target” may introduce bias if benchmark countries have unique demographic structures or data quality issues. Furthermore, the exclusion of “super-efficient” units using a statistical threshold, while necessary to prevent extreme values from artificially lowering the frontier, may inadvertently classify genuinely high-performing countries as outliers. This could raise the estimated efficiency frontier and consequently enlarge the apparent efficiency gap for other countries. Importantly, while the frontier analysis identifies where efficiency gaps exist, it does not elucidate the underlying causes, which may result from variations in health policies, healthcare system organization, resource allocation efficiency, or unmeasured contextual factors such as cultural norms, environmental exposures, or behavioral risks. Third, the Bayesian APC (BAPC) projections to 2035 are based on extrapolating historical trends and cannot account for potential “black swan” events, major technological or policy shifts, or health crises driven by environmental or climate factors. Additionally, the data used in this study extend only to 2021 and therefore capture only the early phase of the COVID-19 pandemic. The pandemic has substantially disrupted healthcare access, social environments, and mental health worldwide. These structural disruptions may alter future NCD trajectories in ways that historical trend-based models cannot fully capture. Consequently, the uncertainty around long-term forecasts, particularly for 2035, may be larger than suggested by the reported credible intervals, and these projections should be interpreted as trend-based scenarios rather than precise predictions. Finally, as with all GBD-based studies, estimates are subject to potential biases arising from incomplete coverage of vital registration systems, health surveys, or variations in case definitions and measurement quality, especially in low-SDI regions. These limitations should be considered when interpreting the study findings and planning future research. In addition, in some low-SDI regions where primary disease registries and surveillance systems are limited, GBD estimates rely more heavily on statistical modeling and covariate-based predictions. Although these models improve comparability across countries, they may partially reflect modeling assumptions rather than directly observed epidemiological data. Therefore, results for some low-SDI regions should be interpreted with caution.

### 4.5 Future research

Building on the findings and limitations of this study, future research should focus on: Utilizing individual-level longitudinal cohort data to deeply investigate the long-term mechanisms through which childhood risk factors (e.g., early life nutrition, childhood adversity) influence adolescent NCD onset. Conducting in-depth mixed-methods case studies in “super-efficient” and high-burden countries to identify replicable and scalable effective health policies and programs. Enhancing surveillance and research on the prevalence and health impacts of emerging risk factors (e.g., e-cigarette use, digital media dependence, climate change-related exposures) among children and adolescents. Promoting the establishment of a more inclusive and sensitive dedicated adolescent health monitoring system, particularly for issues with significant reporting bias like self-harm and substance use.

## 5 Conclusion

This study systematically reveals profound transitions in the global burden of non-communicable diseases among children and adolescents from 1990 to 2021: despite a substantial decline in age-standardized mortality, incidence rates have risen against the trend. Health improvements for the 15–19 age group have generally lagged, with the mental health burden being particularly heavy. Concurrently, the traditional negative correlation between disease burden and SDI is evolving into a new paradox characterized by improving trends in low-SDI regions vs. worsening trends in high-SDI regions. These findings imply that achieving SDG 3.4—reducing premature mortality from NCDs—requires differentiated strategies precisely tailored to national development levels. High-income countries urgently need policy interventions to curb the obesity and mental health crises, while low- and middle-income countries should strengthen primary care efficiency and guard against the spread of metabolic diseases. We call for global action to prioritize adolescent health at its core, vigorously promote school-based mental health services and metabolic risk screening, and couple these efforts with enhanced data monitoring and international collaboration. This concerted approach is essential to building a healthier future for all children and adolescents.

## Data Availability

The original contributions presented in the study are included in the article/Supplementary material, further inquiries can be directed to the corresponding author.
