# Supplementary material for: Global burden and trends of non-communicable diseases among children and adolescents from 1990 to 2021: an age-period-cohort and frontier analysis with projections to 2035
Source: Front Public Health. 2026 Jul 3;14:1698005. doi: 10.3389/fpubh.2026.1698005 (PMC13376241; doi:10.3389/fpubh.2026.1698005)
Supplement: Supplementary file 1 [file Data_Sheet_1.pdf]

Supplementary Methods

Joinpoint regression analysis

Time trend analysis is an important component of epidemiological research. Traditional regression models primarily fit and evaluate the overall trend of disease distribution within the study period from a global perspective, failing to capture local variation characteristics. In 1998, Kim et al. first proposed the Joinpoint regression model. The core idea of this model is to establish segmented regression based on the temporal characteristics of disease distribution. By dividing the study time into different intervals through several Joinpoints, the trend in each interval is fitted and optimized, allowing for a more detailed assessment of the specific disease change characteristics within different intervals of the overall time range.[1] The Joinpoint regression model, developed by the Division of Cancer Control and Population Sciences at the National Cancer Institute of the United States, has been widely applied in the field of trend studies on disease incidence and mortality rates.

(I) Model Introduction

The Joinpoint regression model includes two types: the linear model (y = xb) and the logarithmic linear model (ln y = xb). If the dependent variable follows a normal distribution (or approximately normal distribution) and the sample size is large (usually greater than 100), the linear model is preferred. For example, when the dependent variable is continuous variables like height, weight, etc. If the dependent variable follows an exponential distribution or a Poisson distribution, the logarithmic linear model is more suitable. For instance, when the dependent variable represents epidemiological data based on populations such as incidence rates, number of cases, etc. When analyzing trends in the incidence, prevalence, mortality rates, and DALYs rates of thalassemia based on population data, the logarithmic linear model is generally chosen.

(II) Modeling Method

The grid search method (GSM) is the default modeling approach used by Joinpoint. GSM divides the study data into a grid, with each grid intersection corresponding to a planned scenario[1]. Then, within the specified intervals, it computes performance

metrics for the corresponding equations at each point using a fixed step size to determine the optimal function. In essence, the Joinpoint model uses the GSM to establish all possible segment function Joinpoints (i.e., Joinpoints) and calculates the sum of squares errors (SSE) and mean squared errors (MSE) for each possible scenario. It selects the grid point with the smallest MSE as the Joinpoint for the segment function and fits the equation parameters such as β_0, β_1, δ_1, ..., δ_k based on the selected Joinpoints and interval functions.[2]

(III) Model Optimization

Monte Carlo permutation test is the default model optimization method in Joinpoint software. Before modeling, it is necessary to set the range of the number of Joinpoints k as k ∈ (MIN，MAX) , where MIN represents the minimum number of Joinpoints, which is usually set to 0; MAX represents the maximum number of Joinpoints. Each permutation test checks the null hypothesis H_0: the number of Joinpoints is $k = k_a$, and the alternative hypothesis H_1: the number of Joinpoints is $k = k_b$. The permutation test starts from k_a=MIN and k_b=MAX. If H_0 is rejected, k is set to $k_a + 1$ for further testing; if H_0 is not rejected, k is set to $k_b - 1$ for another test, until $k_a = k_b$, which means $k = k_a = k_b$ is the preferred number of Joinpoints selected by the permutation test, and the corresponding model is the optimal model.[3]

(IV) Index Calculation

Annual percent change (APC) and average annual percent change (AAPC) along with their 95% confidence intervals (CI) are the primary outcome indicators of the Joinpoint model. As the name suggests, APC represents the average annual percentage change of the dependent variable. For example, in a logarithmic linear model ln (y) = $\beta_0 + \beta_1 x$, where y represents the incidence rate and x represents the year of incidence, the formula for calculating APC in the fitted model can be derived as:

$$APC = \left[\frac{y_{x+1} - y_x}{y_x}\right] \times 100 = (e^{\beta_1} - 1) \times 100$$

The lower and upper limits of the 100(1-α) % confidence interval are respectively:

$$APC_{L(\alpha)} = 100(e^{\beta_1 - s \times t_d^{-1}(1-\alpha/2)} - 1)$$
$$APC_{U(\alpha)} = 100(e^{\beta_1 + s \times t_d^{-1}(1-\alpha/2)} - 1)$$

In the above formula, β_1 represents the regression coefficient, s represents the standard error of β_1, d represents the degrees of freedom, and $t_d(q)$ is the value corresponding to the qth percentile of the t-distribution with d degrees of freedom (such as 95%).

The APC is used to evaluate the internal trend of each independent interval of a segmented function or the overall trend with no connecting points. When it comes to assessing the overall average change trend encompassing multiple intervals, the AAPC is required. The parameter calculation method of AAPC involves weighted calculation of the regression coefficients of each interval based on the width w of the segment intervals. Its formula is as follows:

$$AAPC = \left(e^{\sum w_i \beta_i / \sum w_i} - 1\right) \times 100$$

The lower and upper limits of the 100(1-α) % confidence interval are respectively:

$$AAPC_{L(\alpha)} = \left\{\exp\left[\ln((AAPC/100) + 1) - Z_{1-\alpha/2}\sqrt{\sum \tilde{w}_i^2 \tilde{\sigma}_i^2}\right] - 1\right\}$$

$$AAPC_{U(\alpha)} = \left\{\exp\left[\ln((AAPC/100) + 1) + Z_{1-\alpha/2}\sqrt{\sum \tilde{w}_i^2 \tilde{\sigma}_i^2}\right] - 1\right\}$$

In the above formula, w_i represents the width of each segment function interval (i.e., the number of years included in the interval), β_i denotes the regression coefficient corresponding to each interval, $\sigma^2 i$ is the variance of β_i, and Z_α represents the corresponding value of the α percentile in the normal distribution.

(V) Software Download

To download Joinpoint software, we visited the website of the National C

ancer Institute (https://surveillance.cancer.gov/Joinpoint/download), registered, and submitted our application information. Software citation: Joinpoint Regression Program, Version 4.9.1.0 - April 2022; Statistical Methodology and Applications Branch, Surveillance Research Program, National Cancer Institute.

References

1 Kim, H. J., Fay, M. P., Feuer, E. J. & Midthune, D. N. Permutation tests for Joinpoint regression with applications to cancer rates. Stat Med 19, 335-351, doi:10.1002/(sici)1097-0258(20000215)19:3<335::aid-sim336>3.0.co;2-z (2000).

2 Kim, S., Lee, S., Choi, J. I. & Cho, H. Binary genetic algorithm for optimal Joinpoint detection: Application to cancer trend analysis. Stat Med 40, 799-822, doi:10.1002/sim.8803 (2021).

3 Yang, J. J., Trucco, E. M. & Buu, A. A hybrid method of the sequential Monte Carlo and the Edgeworth expansion for computation of very small p-values in permutation tests. Stat Methods Med Res 28, 2937-2951, doi:10.1177/0962280218791918 (2019).
