# Supplementary material for: Global burden and trends of non-communicable diseases among children and adolescents from 1990 to 2021: an age-period-cohort and frontier analysis with projections to 2035
Source: Front Public Health. 2026 Jul 3;14:1698005. doi: 10.3389/fpubh.2026.1698005 (PMC13376241; doi:10.3389/fpubh.2026.1698005)
Supplement: Supplementary file 3 [file Table_2.docx]

**ATERIAL**

**Supplement to “Global burden and trends of non-communicable diseases in children and adolescents from 1990 to 2021: a systematic analysis of the GBD 2021 study”**

MATERIAL 1

Supplementary Table 1 2

Supplementary Table 2 3

Supplementary Table 3 5

Supplementary Table 4 18

Supplementary Table 5 31

Supplementary Table 6 39

Supplementary Table 7 53

Supplementary Table 8 60

Supplementary Table 9 61

Figure S1 62

Figure S2 62

Figure S3 64

Figure S4 65

Figure S5 66

Figure S6 66

Figure S7 67

Supplementary Table 1：The mortality of level-1 and level-2 NCDs and their average annual percentage changes among children and adolescents globally from 1990 to 2021.

| **Location** | **1990-Number** | **1990-ASMR** | **2021-Number** | **2021-ASMR** | **AAPC(95%CI)** | **P** |
| --- | --- | --- | --- | --- | --- | --- |
| Global Level 1 NCD | 1684195(1279344,1979599) | 73.95(56.19,86.91) | 938008(803296,1093414) | 37.31(31.89,43.60) | -2.20 (-2.26 - -2.13) | <0.001 |
| **SDI** |  |  |  |  |  |  |
| High SDI | 81756(78366,85131) | 34.53(33.08,35.98) | 29961(27711,32016) | 13.76(12.68,14.75) | -2.97 (-3.12 - -2.83) | <0.001 |
| High-middle SDI | 223590(187586,250337) | 63.48(53.08,71.17) | 50837(44753,57538) | 18.08(15.84,20.55) | -4.04 (-4.19 - -3.89) | <0.001 |
| Middle SDI | 518652(413710,613309) | 69.25(55.11,82.00) | 185977(161639,214021) | 26.56(22.97,30.72) | -3.08 (-3.16 - -3.00) | <0.001 |
| Low-middle SDI | 518812(377778,610212) | 83.44(61.11,97.98) | 304922(258374,358290) | 41.60(35.16,49.00) | -2.31 (-2.41 - -2.20) | <0.001 |
| Low SDI | 340025(213789,450403) | 107.13(68.47,140.94) | 365406(299871,453432) | 60.94(50.05,75.55) | -1.81 (-1.88 - -1.75) | <0.001 |
| **Sex** |  |  |  |  |  |  |
| Male | 909551(683464,1098166) | 77.64(58.38,93.71) | 500509(423618,595433) | 38.59(32.59,46.03) | -2.29 (-2.33 - -2.25) | <0.001 |
| Female | 774643(569245,945207) | 70.04(51.46,85.47) | 437498(372687,510742) | 35.95(30.57,42.07) | -2.15 (-2.22 - -2.08) | <0.001 |
| **Years** |  |  |  |  |  |  |
| <5 years | 1299511(934052,1561153) | 209.62(150.67,251.82) | 639322(533978,766080) | 97.14(81.13,116.40) | -2.50 (-2.55 - -2.44) | <0.001 |
| 5-9 years | 129978(110997,145720) | 22.27(19.02,24.97) | 78196(68288,87716) | 11.38(9.94,12.77) | -2.19 (-2.34 - -2.04) | <0.001 |
| 10-14 years | 98962(89359,106805) | 18.47(16.68,19.94) | 81772(73934,89180) | 12.27(11.09,13.38) | -1.35 (-1.48 - -1.22) | <0.001 |
| 15-19 years | 155744(144935,165922) | 29.98(27.90,31.94) | 138717(127096,150438) | 22.23(20.37,24.11) | -0.98 (-1.06 - -0.90) | <0.001 |
| **Level 2 - NCDs** |  |  |  |  |  |  |
| Neoplasms | 172625(146300,199989) | 7.62(6.46,8.83) | 114505(96490,131657) | 4.36(3.66,5.03) | -1.82 (-1.91 - -1.72) | <0.001 |
| Cardiovascular diseases | 163751(144947,191190) | 7.19(6.36,8.40) | 81420(70663,93145) | 3.12(2.70,3.59) | -2.68 (-2.74 - -2.61) | <0.001 |
| Chronic respiratory diseases | 57353(44901,65845) | 2.52(1.97,2.89) | 23026(18184,30326) | 0.90(0.71,1.19) | -3.28 (-3.34 - -3.21) | <0.001 |
| Digestive diseases | 122321(102160,142303) | 5.38(4.49,6.26) | 61527(49874,76251) | 2.37(1.90,2.95) | -0.30 (-0.32 - -0.28) | <0.001 |
| Neurological disorders | 43046(31750,50060) | 1.90(1.40,2.20) | 36959(29955,43373) | 1.40(1.13,1.65) | -1.00 (-1.07 - -0.93) | <0.001 |
| Mental disorders | 17(13,20) | 0.00(0.00,0.00) | 18(14,22) | 0.00(0.00,0.00) | -0.34 (-0.58 - -0.09) | 0.007 |
| Musculoskeletal disorders | 3611(2937,4157) | 0.16(0.13,0.18) | 2952(2150,3483) | 0.11(0.08,0.13) | -1.19 (-1.31 - -1.08) | <0.001 |
| Other non-communicable diseases | 1060629(684671,1337212) | 46.52(30.04,58.64) | 573572(484930,696952) | 23.37(19.75,28.41) | -2.22 (-2.28 - -2.17) | <0.001 |
| Skin and subcutaneous diseases | 11058(8472,13871) | 0.49(0.37,0.61) | 6167(4521,8224) | 0.25(0.18,0.33) | -2.23 (-2.40 - -2.07) | <0.001 |
| Sense organ diseases | 0 | 0 | 0 | 0 | 0 |  |
| Substance use disorders | 5098(4629,5630) | 0.22(0.20,0.24) | 3383(3125,3626) | 0.12(0.11,0.13) | -1.93 (-2.18 - -1.68) | <0.001 |
| Diabetes and kidney diseases | 44685(34970,51041) | 1.97(1.54,2.25) | 34480(28570,39683) | 1.31(1.08,1.51) | -1.32 (-1.38 - -1.26) | <0.001 |

Supplementary Table 2：The Disability-Adjusted Life Years of level-1 and level-2 NCDs and their average annual percentage changes among children and adolescents globally from 1990 to 2021.

| **Location** | **1990-Number** | **1990-ASDR** | **2021-Number** | **2021-ASDR** | **AAPC(95%CI)** | ***P*** |
| --- | --- | --- | --- | --- | --- | --- |
| Global Level 1 NCD | 218333221(168040363,264897143) | 9606.35(7395.46,11656.72) | 167506956(136653184,206353456) | 6444.15(5272.27,7920.76) | -1.30 (-1.33, -1.27) | <0.001 |
| **SDI** |  |  |  |  |  |  |
| High SDI | 16610069(13818431,19924472) | 6629.19(5562.16,7897.11) | 12457204(9626364,15811276) | 5198.07(4047.39,6565.56) | -0.71 (-0.77, -0.64) | <0.001 |
| High-middle SDI | 30917843(25101540,36557691) | 8524.47(6931.77,10044.28) | 14092047(11134363,17692882) | 4667.50(3711.50,5831.07) | -1.91 (-1.99, -1.82) | <0.001 |
| Middle SDI | 68715797(54349986,83589303) | 9067.15(7168.73,11022.03) | 39748790(31801262,49616728) | 5362.87(4308.57,6663.29) | -1.71 (-1.74, -1.68) | <0.001 |
| Low-middle SDI | 63514267(47915516,76406296) | 10430.09(7898.33,12565.27) | 50781368(41334396,62408183) | 6723.67(5483.37,8246.02) | -1.42 (-1.46, -1.38) | <0.001 |
| Low SDI | 38393080(26055004,50140777) | 12575.10(8694.44,16323.12) | 50281418(41031356,62300741) | 8501.66(6935.01,10535.32) | -1.27 (-1.31, -1.23) | <0.001 |
|  |  |  |  |  |  |  |
| **Sex** |  |  |  |  |  |  |
| Male | 113691187(86726210,138407045) | 9740.24(7434.25,11858.75) | 84534572(68661484,104026574) | 6329.92(5150.94,7778.63) | -1.39 (-1.41, -1.36) | <0.001 |
| Female | 104642034(78692260,130510928) | 9459.77(7114.26,11798.20) | 82972384(66844035,103507270) | 6563.81(5303.98,8165.90) | -1.18 (-1.21, -1.15) | <0.001 |
| **Years** |  |  |  |  |  |  |
| <5 years | 127100938(94032448,151664991) | 20502.20(15168.04,24464.54) | 67887542(58497803,79966848) | 10314.56(8887.92,12149.85) | -2.23 (-2.29, -2.18) | <0.001 |
| 5-9 years | 24687659(20681718,29834455) | 4230.74(3544.24,5112.75) | 22424559(17934533,27919097) | 3263.87(2610.35,4063.60) | -0.86 (-0.92, -0.80) | <0.001 |
| 10-14 years | 28105036(22385353,35642159) | 5246.58(4178.85,6653.60) | 32633090(25250060,42134955) | 4895.19(3787.68,6320.53) | -0.19 (-0.24, -0.13) | <0.001 |
| 15-19 years | 38439588(30940845,47755538) | 7400.43(5956.77,9193.95) | 44561764(34970788,56332557) | 7141.54(5604.47,9027.94) | -0.06 (-0.11, -0.00) | 0.050 |
| **Level 2 NCDs** |  |  |  |  |  |  |
| Neoplasms | 14265925(12055579,16592390) | 629.70(532.16,732.35) | 9343281(7833676,10786864) | 357.27(298.29,413.97) | -1.85 (-1.94, -1.75) | <0.001 |
| Cardiovascular diseases | 14791658(13127754,17290356) | 649.89(576.69,759.71) | 7990383(6890764,9281018) | 306.21(263.35,356.27) | -2.43 (-2.49, -2.37) | <0.001 |
| Chronic respiratory diseases | 10634801(7844136,14540572) | 469.53(345.92,642.82) | 6715721(4587460,9879396) | 257.90(176.46,378.68) | -1.82 (-1.89, -1.76) | <0.001 |
| Digestive diseases | 11508256(9704786,13290345) | 506.25(426.94,584.59) | 6258205(5122777,7756313) | 240.21(195.61,299.10) | -2.45 (-2.54, -2.35) | <0.001 |
| Neurological disorders | 13078770(6400503,23208442) | 577.40(282.59,1024.79) | 14647098(6621272,27201648) | 542.22(248.57,1000.89) | -0.22 (-0.25, -0.19) | <0.001 |
| Mental disorders | 21256844(15301675,28292189) | 939.55(676.13,1250.73) | 28467435(20281398,38124347) | 1045.90(745.32,1400.67) | 0.54 (0.44, 0.65) | <0.001 |
| Musculoskeletal disorders | 5718525(3733562,8196328) | 251.25(164.03,360.14) | 6900303(4512878,9839202) | 250.10(163.48,356.75) | -0.03 (-0.04, -0.01) | <0.001 |
| Other non-communicable diseases | 103923870(69391567,129971035) | 4560.37(3046.48,5702.85) | 61703161(51998261,74909324) | 2486.56(2098.22,3015.46) | -1.96 (-2.01, -1.91) | <0.001 |
| Skin and subcutaneous diseases | 14511459(9447168,21176235) | 642.20(417.89,937.19) | 16876902(10753411,24792009) | 637.12(406.12,935.55) | -0.04 (-0.05, -0.02) | <0.001 |
| Sense organ diseases | 3311146(2204560,4784444) | 146.55(97.58,211.80) | 4079999(2709776,5917667) | 151.52(100.59,219.86) | 0.11 (0.09, 0.14) | <0.001 |
| Substance use disorders | 1248747(937196,1625425) | 54.00(40.51,70.33) | 1153457(848659,1524750) | 41.48(30.51,54.85) | -0.87 (-0.96, -0.79) | <0.001 |
| Diabetes and kidney diseases | 4083222(3223978,4679601) | 179.64(141.80,205.90) | 3371010(2807705,3933136) | 127.64(105.96,149.14) | -1.10 (-1.16, -1.05) | <0.001 |

Supplementary Table 3: Numbers and ASPR of non-communicable diseases in children and adolescents in 204 countries in 1990 and 2021, along with the Average Annual Percentage Change (AAPC) in ASPR from 1990 to 2021.

| **Location** | **1990-Number** | **1990-ASPR** | **2021-Number** | **2021-ASPR** | **AAPC(95%CI)** | ***P*** |
| --- | --- | --- | --- | --- | --- | --- |
| Cyprus | 204326(195175,213280) | 78115.84(74567.69,81588.47) | 223396(213571,232923) | 78023.15(74555.60,81384.70) | 0.00(-0.01,0.02) | 0.53 |
| Latvia | 552261(517317,588529) | 73154.33(68484.00,77998.43) | 286698(264976,308179) | 73360.17(67667.14,78955.89) | 0.00(-0.02,0.02) | 0.78 |
| Morocco | 9913911(9427689,10391486) | 79771.40(75904.83,83574.40) | 10181200(9657056,10719688) | 78286.82(74152.32,82531.58) | -0.05(-0.06,-0.04) | <0.001 |
| Poland | 9690095(9139415,10242371) | 77313.33(72854.03,81777.97) | 6077928(5760280,6387248) | 78390.71(74196.57,82479.79) | 0.03(0.01,0.06) | 0.01 |
| Mauritania | 944809(907295,981699) | 84459.32(81236.79,87630.80) | 1877932(1790783,1964209) | 80878.78(77178.56,84539.70) | -0.14(-0.14,-0.13) | <0.001 |
| Palestine | 924473(876904,969848) | 78942.91(75059.93,82669.49) | 1903506(1798993,2004821) | 78727.11(74368.18,82949.13) | -0.05(-0.09,0.00) | 0.03 |
| Iran (Islamic Republic of) | 25975741(24857097,27050365) | 82889.27(79430.44,86198.31) | 21577842(20742626,22371030) | 83076.09(79821.27,86140.70) | 0.00(-0.02,0.02) | 0.90 |
| Malta | 86853(82125,91537) | 74276.96(70127.99,78388.56) | 62817(59492,66025) | 74342.94(70354.99,78191.44) | 0.01(,0.00,0.02) | 0.10 |
| Finland | 920159(880047,959405) | 71475.94(68341.74,74544.41) | 871217(827088,914600) | 73551.13(69628.86,77408.67) | 0.10(0.09,0.10) | <0.001 |
| Lithuania | 908326(854197,951731) | 81700.42(76742.13,85660.34) | 416581(388302,445769) | 77241.29(71903.95,82729.37) | -0.23(-0.27,-0.20) | <0.001 |
| Mongolia | 899670(849198,948820) | 79849.35(75440.77,84154.77) | 1010545(944852,1072147) | 78023.51(73141.18,82594.90) | -0.07(-0.08,-0.07) | <0.001 |
| San Marino | 4662(4437,4885) | 74369.17(70402.71,78313.73) | 4856(4619,5089) | 74394.67(70440.56,78322.01) | 0.01(0.00,0.02) | 0.03 |
| Sudan | 8858173(8490003,9208556) | 81403.14(78072.86,84562.56) | 17120105(16219875,18028637) | 79931.77(75711.03,84190.70) | -0.06(-0.07,-0.04) | <0.001 |
| Peru | 8848036(8431796,9232734) | 83144.47(79237.09,86757.14) | 10169714(9651195,10650027) | 81028.22(76853.81,84890.15) | -0.07(-0.08,-0.06) | <0.001 |
| Panama | 881868(837240,924199) | 80871.71(76743.59,84786.88) | 1169668(1101341,1239607) | 76942.10(72376.54,81628.79) | -0.16(-0.17,-0.15) | <0.001 |
| South Africa | 13442722(12798173,14072294) | 76341.66(72682.59,79917.81) | 15353284(14597438,16090468) | 76556.61(72704.27,80308.69) | 0.01(0.00,0.02) | 0.01 |
| Uganda | 8705044(8409911,8984558) | 85496.78(82683.02,88177.75) | 20911234(20128472,21685133) | 84175.62(81069.07,87249.83) | -0.05(-0.06,-0.04) | <0.001 |
| United States Virgin Islands | 34043(32454,35550) | 81344.69(77495.16,84991.04) | 15109(14442,15764) | 81692.83(77908.64,85404.47) | 0.01(0.01,0.02) | <0.001 |
| Oman | 846556(814133,876276) | 85665.96(82546.03,88540.26) | 1252615(1198419,1304804) | 84541.62(81031.14,87921.49) | -0.05(-0.07,-0.04) | <0.001 |
| Bermuda | 12901(12304,13479) | 80889.31(77088.07,84561.80) | 9482(9066,9882) | 81266.03(77519.18,84847.63) | 0.01(0.01,0.02) | <0.001 |
| Brunei Darussalam | 83422(77953,88565) | 73236.75(68542.91,77659.67) | 94935(88971,100877) | 72343.98(67622.36,77055.14) | -0.03(-0.04,-0.03) | <0.001 |
| Uzbekistan | 8339542(7850876,8800854) | 79074.33(74546.65,83353.41) | 9682087(9079187,10283946) | 77591.94(72859.78,82311.97) | -0.06(-0.07,-0.05) | <0.001 |
| Norway | 827897(791687,863022) | 72797.16(69451.07,76062.67) | 944525(901922,985511) | 74204.48(70710.25,77581.04) | 0.07(0.06,0.08) | <0.001 |
| Uruguay | 824328(770117,876777) | 75930.13(70847.20,80842.95) | 704908(660774,747662) | 76428.27(71402.73,81248.98) | 0.03(0.01,0.04) | <0.001 |
| Iraq | 8170164(7713100,8601729) | 80213.16(75862.49,84343.22) | 14215345(13473966,14929969) | 79975.57(75720.48,84070.57) | -0.01(-0.02,0.00) | 0.03 |
| Luxembourg | 66578(63156,69835) | 74739.20(70810.40,78484.42) | 101942(96835,106973) | 74367.86(70535.29,78154.81) | -0.01(-0.02,0.00) | <0.001 |
| New Zealand | 798831(758995,840336) | 71178.34(67454.46,75093.57) | 935054(889599,979341) | 70357.43(66859.93,73775.81) | 0.01(-0.04,0.06) | 0.79 |
| Nepal | 7783666(7239189,8309208) | 76330.77(71192.97,81316.45) | 9304602(8739416,9847057) | 73870.74(69272.24,78248.58) | -0.11(-0.13,-0.10) | <0.001 |
| Samoa | 73373(69301,77301) | 80214.39(75765.15,84510.48) | 80301(75632,84901) | 80031.18(75455.78,84552.69) | -0.01(-0.01,-0.01) | <0.001 |
| Venezuela (Bolivarian Republic of) | 7249084(6899447,7597853) | 80023.46(76176.10,83863.46) | 6770556(6392514,7156205) | 76627.50(72245.21,81105.44) | -0.13(-0.14,-0.12) | <0.001 |
| Indonesia | 72311547(69173662,75400191) | 82465.77(78867.03,86004.49) | 72887379(69271642,76380386) | 80087.08(75978.89,84042.74) | -0.09(-0.11,-0.08) | <0.001 |
| Belarus | 2391857(2219107,2562005) | 75914.37(70374.62,81355.62) | 1542821(1435444,1653531) | 75617.60(70254.25,81074.39) | -0.01(-0.02,0.00) | <0.001 |
| Saudi Arabia | 6976036(6796145,7162862) | 85539.26(83345.75,87805.59) | 8507108(8202630,8808252) | 83132.88(80102.52,86144.87) | -0.10(-0.12,-0.07) | <0.001 |
| Ghana | 6907872(6662220,7146133) | 84482.35(81591.89,87310.46) | 13128878(12558077,13703819) | 80800.00(77332.28,84296.55) | -0.14(-0.15,-0.13) | <0.001 |
| Lebanon | 1057746(999480,1114137) | 79428.29(75096.29,83628.60) | 1332264(1259795,1402112) | 79326.28(74925.69,83557.75) | -0.01(-0.02,0.00) | 0.07 |
| Yemen | 6750992(6410540,7073413) | 81832.46(77938.43,85549.12) | 13948959(13186562,14699207) | 79894.53(75554.37,84165.63) | -0.07(-0.08,-0.07) | <0.001 |
| Singapore | 672399(634692,710271) | 71012.05(66714.08,75323.34) | 722776(677163,765728) | 69517.79(65186.74,73599.13) | -0.05(-0.08,-0.03) | <0.001 |
| Malaysia | 6720371(6405315,7058736) | 80861.39(77105.03,84896.52) | 7955189(7482371,8425143) | 76667.93(71934.61,81338.45) | -0.18(-0.19,-0.17) | <0.001 |
| Andorra | 10483(9963,10977) | 74801.90(70771.66,78612.95) | 11291(10750,11797) | 74529.96(70593.94,78213.94) | -0.01(-0.02,0.00) | <0.001 |
| Ireland | 1039970(990574,1090692) | 76147.95(72351.82,80066.19) | 1023495(970768,1075101) | 75749.68(71681.50,79742.40) | -0.01(-0.02,0.00) | 0.16 |
| Iceland | 65739(62633,68980) | 76817.80(73084.09,80738.33) | 68395(64978,71656) | 75597.32(71716.59,79312.06) | -0.06(-0.07,-0.05) | <0.001 |
| Romania | 6419062(6103671,6700482) | 84797.00(80526.66,88625.86) | 3254935(3058242,3443339) | 79727.81(74775.09,84446.58) | -0.21(-0.24,-0.18) | <0.001 |
| Mozambique | 6411296(6169110,6639361) | 85589.73(82449.38,88554.25) | 14222453(13537882,14946791) | 80723.07(76926.49,84732.79) | -0.19(-0.22,-0.16) | <0.001 |
| Lesotho | 639180(603718,675570) | 77149.99(73005.70,81419.30) | 632548(597057,673856) | 74491.80(70198.92,79511.78) | -0.11(-0.13,-0.10) | <0.001 |
| Madagascar | 6161724(6024085,6276208) | 92231.36(90233.70,93898.32) | 12460064(11940105,12994205) | 83485.56(80019.92,87041.99) | -0.33(-0.36,-0.31) | <0.001 |
| Portugal | 2341989(2236575,2445382) | 75341.04(71651.32,78988.43) | 1486639(1421006,1549821) | 75057.40(71496.40,78502.74) | -0.01(-0.02,0.01) | 0.31 |
| Saint Kitts and Nevis | 15032(14327,15699) | 81499.76(77634.42,85141.70) | 11661(11149,12154) | 81412.15(77606.29,85062.97) | -0.01(-0.02,0.01) | 0.32 |
| Democratic People's Republic of Korea | 5957045(5655551,6265100) | 76676.56(72825.66,80640.25) | 4952701(4665007,5236721) | 74019.25(69480.38,78462.99) | -0.11(-0.12,-0.10) | <0.001 |
| Sao Tome and Principe | 59009(56736,61163) | 84967.66(81775.43,87996.65) | 83263(80134,86400) | 81226.86(78144.52,84309.35) | -0.15(-0.16,-0.13) | <0.001 |
| Syrian Arab Republic | 5841501(5526538,6144240) | 80162.11(75960.62,84212.90) | 4488174(4264704,4695250) | 79630.90(75160.94,83751.69) | -0.03(-0.05,-0.01) | <0.001 |
| Namibia | 583185(553600,613494) | 77137.84(73290.79,81092.27) | 802702(758168,851501) | 74569.77(70391.66,79156.47) | -0.11(-0.13,-0.09) | <0.001 |
| Botswana | 574369(545691,603628) | 78047.62(74221.94,81961.91) | 708061(671893,744589) | 76792.45(72806.72,80805.79) | -0.05(-0.06,-0.04) | <0.001 |
| North Macedonia | 565836(530305,597418) | 80468.34(75331.59,85050.34) | 349364(326818,370881) | 76985.83(71837.35,81888.74) | -0.12(-0.13,-0.11) | <0.001 |
| C么te d'Ivoire | 5645416(5414470,5873645) | 82958.76(79723.21,86179.74) | 11593470(11090641,12090347) | 81356.98(77924.01,84765.15) | -0.06(-0.07,-0.05) | <0.001 |
| Cook Islands | 6877(6469,7271) | 79576.26(74796.00,84177.68) | 4184(3956,4413) | 79310.43(74748.05,83833.52) | -0.01(-0.02,-0.01) | <0.001 |
| Micronesia (Federated States of) | 46282(43621,48811) | 80680.88(76102.75,85040.17) | 33729(31880,35492) | 80284.52(75683.75,84613.33) | -0.01(-0.02,-0.01) | <0.001 |
| Brazil | 55727737(53478132,57859053) | 82469.28(79116.17,85649.74) | 52367322(50136530,54422954) | 81497.11(77956.62,84760.45) | -0.04(-0.06,-0.01) | <0.001 |
| United States of America | 55638029(52872163,58240731) | 74730.30(70962.63,78285.85) | 63294171(60675486,65838542) | 76179.26(72847.56,79434.03) | 0.10(0.07-0.13) | <0.001 |
| Kyrgyzstan | 1640058(1534962,1739865) | 78394.14(73469.11,83095.68) | 2189792(2054405,2322527) | 78078.38(73345.71,82711.83) | -0.01(-0.02,-0.01) | <0.001 |
| Saint Lucia | 54842(52325,57228) | 81966.49(78181.98,85552.45) | 34774(33410,36037) | 81201.22(77870.51,84270.61) | -0.03(-0.03,-0.03) | <0.001 |
| Taiwan (Province of China) | 5459966(5162274,5756746) | 73642.88(69386.36,77838.25) | 3089246(2915269,3274760) | 75189.37(70814.59,79838.54) | 0.09(0.08-0.11) | <0.001 |
| Kuwait | 512851(485894,539969) | 75851.81(71971.94,79753.42) | 850203(800182,899836) | 76885.95(72291.55,81442.15) | 0.05(0.03,0.06) | <0.001 |
| Kazakhstan | 5105967(4769490,5424994) | 77093.52(72041.60,81890.69) | 5093923(4750101,5421436) | 76401.34(71347.70,81214.99) | -0.03(-0.06,-0.01) | <0.001 |
| Angola | 5066217(4914324,5208819) | 88662.80(86079.70,91104.64) | 15794980(15224358,16383362) | 85221.41(82246.99,88285.35) | -0.15(-0.15,-0.14) | <0.001 |
| Palau | 4982(4704,5249) | 79905.37(75311.07,84305.89) | 3593(3395,3789) | 79749.39(75125.34,84276.81) | -0.01(-0.01,0.00) | 0.10 |
| Burkina Faso | 4965152(4828298,5098517) | 88622.53(86278.76,90930.16) | 10791713(10411129,11180483) | 85095.44(82177.10,88071.41) | -0.17(-0.20,-0.13) | <0.001 |
| Guinea-Bissau | 490438(469728,510180) | 84558.05(81122.29,87810.27) | 897631(851314,942250) | 81017.12(76945.61,84936.46) | -0.13(-0.15,-0.12) | <0.001 |
| Bangladesh | 48767034(46202045,51314329) | 81612.23(77466.01,85762.18) | 49137213(46505389,51699997) | 79827.61(75412.81,84096.39) | -0.07(-0.08,-0.07) | <0.001 |
| Cambodia | 4855697(4665353,5031326) | 86186.47(82944.73,89195.07) | 5573135(5328781,5811729) | 83894.55(80190.07,87508.70) | -0.12(-0.15,-0.10) | <0.001 |
| Cameroon | 4826459(4619678,5036430) | 82675.18(79280.69,86135.37) | 13528534(12914154,14144931) | 80505.43(76914.46,84117.60) | -0.08(-0.09,-0.08) | <0.001 |
| Saint Vincent and the Grenadines | 47257(45363,48764) | 87127.43(83539.51,89982.02) | 27981(26673,29252) | 81707.47(77720.58,85554.05) | -0.22(-0.25,-0.18) | <0.001 |
| Pakistan | 47015629(44179642,49712057) | 77856.45(73308.97,82204.08) | 81889282(77569021,86218976) | 75069.02(71121.85,79021.27) | -0.11(-0.13,-0.09) | <0.001 |
| Argentina | 9942148(9320946,10523313) | 76124.88(71350.10,80588.93) | 10637738(9959643,11276082) | 75990.16(70929.17,80718.82) | 0.01(-0.02,0.03) | 0.46 |
| Malawi | 4634892(4435773,4816811) | 84268.76(80741.25,87496.78) | 8720515(8293736,9125628) | 82053.66(77998.55,85903.97) | -0.09(-0.10,-0.08) | <0.001 |
| Greenland | 13080(12299,13829) | 74230.36(69938.78,78351.43) | 11360(10686,12031) | 73530.06(69110.03,77934.04) | -0.01(-0.04,0.02) | 0.38 |
| Zimbabwe | 4563961(4356184,4771354) | 76452.82(73047.77,79861.63) | 5980959(5664766,6303323) | 75148.67(71209.46,79168.27) | -0.05(-0.07,-0.04) | <0.001 |
| Gambia | 449805(429201,471269) | 81543.79(77977.44,85254.02) | 1007474(956805,1057410) | 79803.63(75820.76,83729.09) | -0.07(-0.08,-0.07) | <0.001 |
| Afghanistan | 4437641(4203545,4664796) | 80302.00(76086.26,84401.49) | 13893712(13092142,14677581) | 79167.11(74748.47,83487.73) | -0.04(-0.05,-0.03) | <0.001 |
| Gabon | 441901(429853,453824) | 87437.73(85110.39,89756.69) | 701571(679728,724578) | 84644.70(81991.97,87443.24) | -0.11(-0.12,-0.11) | <0.001 |
| Slovenia | 440945(419223,463213) | 77968.97(73931.60,82098.23) | 311393(290403,331627) | 76374.33(71163.30,81384.40) | -0.05(-0.07,-0.04) | <0.001 |
| Northern Mariana Islands | 12816(12122,13495) | 79223.12(74894.26,83459.58) | 12153(11482,12818) | 79772.90(75166.05,84275.92) | -0.01(-0.04,0.02) | 0.46 |
| Italy | 11125680(10738353,11505337) | 78660.39(75655.58,81626.70) | 8365944(8065821,8659296) | 77574.48(74619.27,80505.92) | -0.01(-0.05,0.02) | 0.43 |
| Bahamas | 88821(84848,92423) | 81694.10(77948.30,85082.30) | 97258(93155,100990) | 82175.12(78409.86,85608.16) | 0.02(,0.00,0.04) | 0.06 |
| Sri Lanka | 5959820(5671904,6241385) | 81782.80(77751.30,85712.72) | 5643866(5382852,5901106) | 80615.58(76717.92,84452.85) | 0.02(-0.01,0.04) | 0.20 |
| Niger | 4152376(4025481,4273686) | 86964.20(84447.40,89404.89) | 12936429(12427855,13436598) | 84126.76(80968.98,87267.92) | -0.11(-0.12,-0.10) | <0.001 |
| Ecuador | 4115184(3914187,4299412) | 83019.16(78969.73,86732.41) | 5375679(5174947,5565357) | 80694.82(77689.89,83536.68) | -0.11(-0.13,-0.09) | <0.001 |
| Mali | 4111370(3937503,4281222) | 84794.74(81400.33,88117.95) | 11674776(11151633,12198986) | 83212.78(79634.34,86806.51) | -0.07(-0.07,-0.06) | <0.001 |
| Nauru | 4103(3866,4335) | 79846.69(75385.19,84213.40) | 4083(3848,4315) | 79612.48(75031.70,84144.99) | -0.01(-0.01,-0.01) | <0.001 |
| Zambia | 4048555(3924735,4169025) | 86808.21(84222.31,89324.28) | 8877610(8536237,9203987) | 85111.33(81878.11,88200.35) | -0.07(-0.07,-0.06) | <0.001 |
| Somalia | 4011880(3849021,4162503) | 85659.51(82273.62,88793.38) | 10458195(9998254,10939398) | 82882.79(79348.70,86576.71) | -0.11(-0.12,-0.10) | <0.001 |
| United Arab Emirates | 560556(531818,588121) | 80827.86(76949.70,84571.10) | 1379517(1315672,1440727) | 81226.70(77492.96,84804.02) | 0.02(-0.01,0.05) | 0.17 |
| Guatemala | 3851882(3643857,4047841) | 79609.52(75485.66,83526.98) | 5125186(4818037,5416426) | 76437.41(71703.50,80906.18) | -0.13(-0.13,-0.12) | <0.001 |
| Australia | 3751125(3636024,3869232) | 70957.30(68730.84,73244.63) | 4803389(4571457,5007580) | 75857.75(72124.12,79159.30) | 0.35(0.23,0.46) | <0.001 |
| Eswatini | 373830(354587,392781) | 79160.75(75215.04,83081.53) | 416601(394251,438174) | 77663.87(73462.26,81711.50) | -0.06(-0.06,-0.05) | <0.001 |
| Senegal | 3737444(3605653,3864511) | 85111.30(82247.13,87871.32) | 6632198(6355323,6906206) | 81893.64(78496.95,85255.62) | -0.14(-0.15,-0.12) | <0.001 |
| Monaco | 3647(3461,3828) | 73702.93(69786.72,77524.39) | 5132(4878,5381) | 74302.16(70465.73,78053.42) | 0.04(0.03-0.04) | <0.001 |
| Rwanda | 3550695(3418920,3664548) | 86601.12(83461.72,89334.12) | 5176271(4931563,5426165) | 80367.74(76561.65,84256.07) | -0.24(-0.27,-0.22) | <0.001 |
| China | 352747635(334969514,371213773) | 78636.41(74515.55,82897.78) | 257974132(245135568,271049615) | 76881.20(72987.94,80803.25) | -0.07(-0.08,-0.05) | <0.001 |
| Canada | 5591096(5299672,5884632) | 72047.22(68195.13,75925.88) | 6068364(5720137,6404084) | 72308.85(67999.28,76463.66) | 0.02(0.01,0.02) | <0.001 |
| Mauritius | 349767(332308,366998) | 80614.26(76501.18,84653.74) | 236547(224146,249125) | 77819.15(73403.52,82258.01) | -0.11(-0.12,-0.09) | <0.001 |
| Guam | 42597(40209,44975) | 79157.19(74752.12,83556.31) | 38682(36547,40738) | 79408.55(74946.40,83701.34) | 0.02(0.01,0.02) | <0.001 |
| Sweden | 1596870(1519702,1675365) | 75032.39(71279.46,78861.03) | 1842799(1751596,1935680) | 75141.78(71315.42,79043.96) | 0.02(0.01,0.03) | <0.001 |
| Chile | 4011319(3775576,4250942) | 75871.54(71363.44,80451.44) | 3805109(3601510,4000213) | 76354.83(72089.68,80432.58) | 0.02(0.01,0.04) | <0.001 |
| Tuvalu | 3389(3201,3565) | 81094.81(76790.90,85163.87) | 3945(3720,4157) | 79970.50(75352.05,84321.78) | -0.04(-0.05,-0.04) | <0.001 |
| Vanuatu | 66179(62391,69753) | 80617.36(76191.58,84835.80) | 117545(110755,124156) | 80130.20(75574.67,84578.42) | -0.02(-0.02,-0.01) | <0.001 |
| Grenada | 34877(33346,36349) | 83047.71(79444.25,86512.34) | 25173(24093,26204) | 82560.99(78853.92,86088.95) | -0.02(-0.02,-0.01) | <0.001 |
| Tunisia | 3248911(3087403,3406559) | 81371.78(77326.63,85318.09) | 2910819(2763106,3054255) | 80707.01(76551.22,84726.06) | -0.02(-0.02,-0.01) | <0.001 |
| Kiribati | 29511(27970,31085) | 81667.07(77559.38,85899.97) | 43429(41145,45646) | 81260.60(77002.87,85393.99) | -0.02(-0.02,-0.01) | <0.001 |
| Guyana | 310683(296284,324679) | 81727.86(77933.70,85428.92) | 229470(218922,239291) | 81504.36(77715.06,85030.24) | -0.01(-0.01,-0.01) | <0.001 |
| Cuba | 2979180(2837109,3112900) | 80369.80(76312.76,84179.85) | 1951359(1853139,2046288) | 80229.82(76047.86,84249.86) | -0.01(-0.03,0.01) | 0.20 |
| Trinidad and Tobago | 418386(398457,438111) | 80411.87(76579.01,84201.73) | 294104(280753,307529) | 79575.09(75797.98,83379.34) | 0.02(-0.02,0.06) | 0.33 |
| Chad | 2899996(2778965,3019316) | 83890.64(80560.46,87179.64) | 8812795(8416125,9201627) | 81921.83(78392.21,85375.51) | -0.08(-0.08,-0.07) | <0.001 |
| Haiti | 2885044(2785411,2977149) | 86890.57(83958.03,89622.38) | 4804521(4632184,4968767) | 85783.79(82714.60,88715.27) | -0.05(-0.05,-0.05) | <0.001 |
| Dominican Republic | 2879675(2749635,3006626) | 82350.37(78621.24,85999.57) | 3162674(3017150,3305663) | 81004.22(77225.72,84716.65) | -0.05(-0.05,-0.04) | <0.001 |
| Fiji | 286199(268921,302522) | 79705.21(74903.97,84234.16) | 279002(262272,294892) | 79363.09(74580.96,83895.10) | -0.04(-0.05,-0.03) | <0.001 |
| Estonia | 345718(321326,369182) | 74983.57(69641.24,80129.35) | 210722(195847,225392) | 74742.97(69370.36,80040.99) | -0.02(-0.03,0.00) | 0.01 |
| Viet Nam | 27515839(26127186,28844060) | 81943.29(77856.47,85860.17) | 24831726(23401817,26224519) | 78413.60(73911.09,82786.61) | -0.15(-0.16,-0.14) | <0.001 |
| South Sudan | 2751482(2637164,2863202) | 84295.22(80838.55,87678.03) | 4492533(4284646,4692954) | 82074.61(78279.04,85733.04) | -0.09(-0.10,-0.09) | <0.001 |
| Guinea | 2748620(2644367,2850844) | 85130.09(82063.57,88158.87) | 6185908(5935848,6434266) | 83594.81(80313.38,86855.79) | -0.07(-0.08,-0.06) | <0.001 |
| Bolivia (Plurinational State of) | 2732819(2595354,2858781) | 82294.75(78214.61,86039.83) | 3686747(3487229,3862247) | 80823.05(76413.90,84697.25) | -0.05(-0.06,-0.04) | <0.001 |
| Dominica | 26754(25489,27920) | 81320.43(77418.17,84919.03) | 16270(15567,16959) | 81550.46(77687.34,85289.14) | 0.01(0.01,0.01) | <0.001 |
| Bhutan | 265367(249347,281407) | 77952.29(73249.35,82676.28) | 191566(180228,203468) | 73939.04(69390.46,78704.39) | -0.17(-0.18,-0.16) | <0.001 |
| Burundi | 2640690(2528907,2745378) | 84855.36(81385.04,88122.69) | 5837811(5549426,6140983) | 80904.86(76996.41,85012.62) | -0.16(-0.17,-0.15) | <0.001 |
| Philippines | 26285887(25172445,27405935) | 82292.75(78836.49,85767.17) | 35568904(33991996,37116205) | 78940.58(75373.62,82438.51) | -0.14(-0.16,-0.12) | <0.001 |
| Belize | 83864(80021,87452) | 82202.87(78501.59,85663.44) | 139951(133801,145641) | 81589.66(77813.28,85080.45) | -0.02(-0.03,-0.01) | <0.001 |
| Ethiopia | 25712703(24737144,26611393) | 87949.12(84730.81,90913.71) | 48180053(46165153,50025851) | 84329.65(80806.04,87562.79) | -0.14(-0.15,-0.13) | <0.001 |
| Japan | 25492144(24248875,26693472) | 74010.19(70129.08,77799.16) | 15373385(14631205,16130534) | 70377.91(66800.61,74069.22) | -0.15(-0.17,-0.12) | <0.001 |
| Seychelles | 25267(23957,26539) | 80504.35(76260.56,84619.00) | 24139(22786,25468) | 78730.47(74254.19,83118.51) | -0.08(-0.08,-0.07) | <0.001 |
| Republic of Moldova | 1209648(1125979,1292212) | 76582.66(71306.20,81786.14) | 536251(498832,573813) | 75940.25(70434.20,81432.96) | -0.02(-0.03,-0.01) | <0.001 |
| Benin | 2402328(2311462,2496231) | 85174.61(82124.97,88316.17) | 6134218(5861011,6395618) | 82507.86(78947.24,85923.74) | -0.10(-0.11,-0.09) | <0.001 |
| Tonga | 42792(40382,45143) | 80514.59(76013.79,84917.91) | 39372(37089,41587) | 79858.28(75288.97,84303.75) | -0.02(-0.03,-0.02) | <0.001 |
| Antigua and Barbuda | 19640(18741,20510) | 81812.12(78013.77,85481.38) | 19293(18445,20101) | 81326.68(77561.54,84899.96) | -0.02(-0.03,-0.02) | <0.001 |
| Libya | 1923156(1841101,2004028) | 83418.67(79874.73,86914.21) | 1761607(1688256,1832248) | 82765.26(79062.92,86330.36) | -0.02(-0.03,-0.02) | <0.001 |
| Hungary | 2332978(2199309,2461617) | 78912.99(74169.90,83524.37) | 1455996(1361857,1547816) | 76736.33(71631.98,81709.53) | -0.10(-0.11,-0.08) | <0.001 |
| Serbia | 2326965(2176176,2452191) | 79027.92(73732.17,83433.18) | 1434812(1348834,1519716) | 73830.91(69176.66,78439.27) | -0.25(-0.34,-0.16) | <0.001 |
| Greece | 2305163(2249281,2360530) | 78920.82(76818.65,81058.11) | 1454284(1398065,1509600) | 73813.44(70861.53,76736.16) | -0.21(-0.23,-0.19) | <0.001 |
| Egypt | 22600915(21509029,23683442) | 81676.29(77817.56,85524.69) | 36747120(34769053,38738051) | 79303.34(75103.04,83523.26) | -0.09(-0.10,-0.09) | <0.001 |
| Jordan | 1668487(1595354,1743023) | 79937.73(76441.25,83496.68) | 3976533(3774151,4179574) | 79316.82(75063.80,83566.10) | -0.02(-0.03,-0.02) | <0.001 |
| El Salvador | 2213914(2102919,2326453) | 80492.86(76466.37,84575.95) | 1846874(1735860,1949065) | 77032.51(72342.25,81342.83) | -0.14(-0.15,-0.12) | <0.001 |
| Turkey | 21974792(20954843,22927466) | 82086.51(78238.51,85682.76) | 20164400(19157210,21147386) | 80489.58(76316.55,84547.54) | -0.05(-0.07,-0.04) | <0.001 |
| Comoros | 218608(209215,227646) | 83425.28(79940.85,86775.97) | 255479(242935,267094) | 81100.64(77070.79,84831.79) | -0.09(-0.10,-0.09) | <0.001 |
| Spain | 8714512(8368927,9047500) | 73763.96(70564.03,76879.28) | 6680766(6322902,7014911) | 73051.18(68904.25,76930.13) | -0.02(-0.04,0.00) | 0.02 |
| Honduras | 2127930(2013151,2240210) | 79312.58(75159.40,83389.39) | 3355605(3153572,3549906) | 76441.32(71726.92,80965.26) | -0.12(-0.13,-0.11) | <0.001 |
| India | 335263356(320259351,349069613) | 82020.26(78413.62,85341.26) | 413867312(399288924,427483862) | 81633.35(78657.62,84413.28) | -0.02(-0.04,0.00) | 0.01 |
| Equatorial Guinea | 203276(195198,210931) | 85780.55(82477.59,88923.88) | 633388(606786,661075) | 81723.73(78226.99,85366.98) | -0.16(-0.16,-0.15) | <0.001 |
| Denmark | 923308(881794,965770) | 71390.70(67983.54,74897.69) | 950257(909708,991363) | 71564.64(68402.91,74786.37) | -0.02(-0.05,0.00) | 0.11 |
| Sierra Leone | 1928576(1872071,1980043) | 88767.47(86343.38,90971.51) | 3819613(3705286,3932476) | 84331.25(81829.62,86801.02) | -0.16(-0.17,-0.15) | <0.001 |
| Thailand | 19245748(18543281,20009650) | 83731.30(80615.75,87129.54) | 11251892(10813748,11714504) | 80784.52(77552.01,84238.07) | -0.11(-0.12,-0.10) | <0.001 |
| Czechia | 2401366(2247112,2552824) | 77137.43(71938.91,82265.10) | 1712079(1598234,1827926) | 76682.74(71534.10,81931.78) | -0.02(-0.05,0.00) | 0.07 |
| Democratic Republic of the Congo | 19223098(18684627,19732124) | 89513.67(87078.88,91820.52) | 41103380(39743457,42539534) | 86052.45(83244.78,89016.30) | -0.12(-0.13,-0.11) | <0.001 |
| Bulgaria | 1903783(1788949,2009466) | 79294.27(74313.49,83867.12) | 1018054(954692,1079646) | 77735.94(72767.62,82554.81) | -0.08(-0.10,-0.06) | <0.001 |
| Lao People's Democratic Republic | 1876384(1791354,1960996) | 83512.98(79844.71,87185.96) | 2418269(2299614,2536735) | 80850.96(76875.38,84825.41) | -0.09(-0.11,-0.08) | <0.001 |
| American Samoa | 18729(17614,19792) | 79340.35(74738.53,83757.04) | 15454(14566,16309) | 79042.51(74231.02,83602.29) | -0.03(-0.05,-0.01) | <0.001 |
| Djibouti | 186028(178454,193253) | 83152.23(79772.32,86376.62) | 418551(397862,439657) | 79332.12(75430.22,83311.03) | -0.15(-0.16,-0.13) | <0.001 |
| Belgium | 1818066(1731612,1901294) | 71847.82(68300.81,75261.85) | 1890310(1790454,1984522) | 72638.77(68680.49,76382.90) | 0.04(0.03,0.06) | <0.001 |
| Togo | 1811482(1744503,1877048) | 85090.60(82049.94,88072.95) | 3391269(3256741,3528326) | 81522.39(78337.09,84763.10) | -0.14(-0.17,-0.11) | <0.001 |
| Papua New Guinea | 1775655(1697553,1853482) | 84056.20(80435.51,87690.47) | 3988612(3786772,4185840) | 82198.19(78142.65,86203.81) | -0.06(-0.07,-0.06) | <0.001 |
| Nicaragua | 1769396(1674813,1864048) | 79332.15(75196.24,83481.53) | 1985355(1864449,2109377) | 75761.47(71067.89,80581.29) | -0.15(-0.15,-0.14) | <0.001 |
| Montenegro | 173810(163044,183362) | 80277.06(75202.32,84792.44) | 116986(109349,124114) | 76809.75(71646.96,81622.92) | -0.14(-0.16,-0.12) | <0.001 |
| Suriname | 140041(133572,146347) | 81596.94(77789.05,85296.26) | 155964(149060,162675) | 81413.27(77694.91,85011.79) | -0.02(-0.06,0.01) | 0.14 |
| Barbados | 69953(66779,72991) | 81336.82(77498.23,85001.48) | 55503(53153,57728) | 81955.64(78246.76,85446.21) | 0.03(0.02,0.03) | <0.001 |
| Georgia | 1403180(1315686,1490322) | 77781.45(72879.60,82661.02) | 729124(683711,770972) | 78454.98(73590.91,82929.42) | 0.03(0.02,0.03) | <0.001 |
| Eritrea | 1655179(1582144,1720779) | 84937.85(81272.34,88241.13) | 2601262(2466163,2730168) | 81207.50(77023.93,85192.92) | -0.14(-0.16,-0.13) | <0.001 |
| Austria | 1407498(1333377,1480576) | 73451.57(69403.72,77436.52) | 1321201(1251538,1388872) | 74026.03(69985.11,77955.68) | 0.03(0.02,0.04) | <0.001 |
| Jamaica | 922241(885466,956104) | 84111.65(80720.08,87229.48) | 690925(662730,717454) | 83344.38(79727.06,86719.73) | -0.03(-0.03,-0.02) | <0.001 |
| Niue | 820(772,867) | 80025.63(75302.28,84540.72) | 426(402,449) | 79409.69(74809.53,83941.99) | -0.03(-0.03,-0.02) | <0.001 |
| Israel | 1557169(1478601,1633095) | 77070.51(73124.37,80892.61) | 2618344(2478006,2754543) | 77678.22(73527.64,81707.31) | 0.03(0.00,0.05) | 0.02 |
| Myanmar | 15561400(14851984,16242588) | 81282.19(77557.67,84855.64) | 16770486(16012240,17533859) | 80217.66(76507.09,83950.30) | 0.00(-0.05,0.05) | 0.94 |
| Cabo Verde | 154064(146130,161883) | 80166.57(76175.21,84110.86) | 151212(143111,159370) | 77223.13(72918.39,81547.53) | -0.12(-0.12,-0.11) | <0.001 |
| Tajikistan | 2229394(2095701,2362966) | 78785.59(74203.59,83392.42) | 3444273(3235383,3652556) | 78088.24(73451.92,82716.93) | -0.03(-0.03,-0.02) | <0.001 |
| Turkmenistan | 1468018(1380138,1553600) | 78655.00(74029.64,83170.84) | 1517521(1422256,1610307) | 77487.43(72634.86,82215.18) | -0.05(-0.05,-0.04) | <0.001 |
| Marshall Islands | 21517(20270,22713) | 80825.56(76275.55,85201.07) | 18668(17619,19668) | 80272.30(75652.67,84655.64) | -0.03(-0.03,-0.02) | <0.001 |
| Solomon Islands | 158926(150644,166660) | 82445.60(78263.36,86372.76) | 269354(255561,282601) | 81800.42(77660.52,85789.44) | -0.03(-0.03,-0.02) | <0.001 |
| Russian Federation | 35045206(32823431,37121950) | 77300.88(72356.63,81917.36) | 26044636(24538413,27503090) | 76487.53(71980.96,80822.84) | -0.03(-0.04,-0.02) | <0.001 |
| Slovakia | 1372795(1283110,1460337) | 77276.77(72110.12,82318.17) | 863278(805185,917680) | 76520.81(71294.50,81413.67) | -0.03(-0.05,-0.01) | <0.001 |
| Azerbaijan | 2441788(2293405,2588410) | 78285.54(73556.65,82967.05) | 2382661(2235501,2526439) | 77698.83(72847.26,82407.55) | -0.03(-0.04,-0.02) | <0.001 |
| Germany | 13159774(12665592,13644438) | 75166.23(72292.85,77991.39) | 11803918(11350224,12287654) | 73056.94(70194.58,76119.44) | -0.09(-0.12,-0.07) | <0.001 |
| Bahrain | 170812(164554,176655) | 86137.13(83107.43,88977.21) | 347448(335193,359625) | 85301.51(82169.58,88398.57) | -0.03(-0.04,-0.02) | <0.001 |
| Central African Republic | 1305668(1263663,1345935) | 88030.87(85266.00,90685.98) | 2471584(2380722,2561752) | 85702.78(82583.43,88794.49) | -0.09(-0.09,-0.08) | <0.001 |
| Albania | 1118961(1042234,1190302) | 77489.61(72181.73,82428.75) | 481338(451206,510601) | 76622.38(71633.84,81464.48) | -0.03(-0.04,-0.02) | <0.001 |
| Tokelau | 611(576,645) | 80482.64(75927.68,84897.15) | 414(393,435) | 79527.61(75303.34,83476.21) | -0.03(-0.05,-0.02) | <0.001 |
| Republic of Korea | 12577509(11894444,13248873) | 77346.46(72957.81,81680.75) | 6451197(6098690,6817762) | 75063.97(70718.18,79589.44) | -0.09(-0.11,-0.07) | <0.001 |
| United Republic of Tanzania | 12525916(12207652,12854512) | 84739.92(82634.25,86907.64) | 25593036(24626382,26594493) | 83241.21(80128.05,86466.75) | -0.06(-0.09,-0.03) | <0.001 |
| France | 12301247(11916695,12685462) | 74471.81(72059.98,76900.27) | 11897894(11305299,12491520) | 73272.28(69464.18,77106.41) | -0.03(-0.05,-0.01) | <0.001 |
| Bosnia and Herzegovina | 1221443(1148669,1287467) | 81487.54(76515.61,86047.58) | 526671(493413,560008) | 78046.20(72972.71,83152.15) | -0.14(-0.15,-0.13) | <0.001 |
| Timor-Leste | 331817(315508,346887) | 83029.17(79095.56,86671.82) | 563449(533530,590791) | 82069.62(77657.01,86096.90) | -0.04(-0.05,-0.02) | <0.001 |
| Colombia | 11959250(11353078,12551022) | 79560.89(75531.86,83498.33) | 11375470(10897237,11852678) | 76869.60(73577.05,80154.05) | -0.11(-0.15,-0.07) | <0.001 |
| Qatar | 119205(112571,125717) | 79408.31(75195.00,83585.19) | 473064(448000,495412) | 80342.36(76292.54,83999.66) | 0.04(0.03,0.05) | <0.001 |
| Switzerland | 1191264(1137574,1244696) | 74302.61(70831.05,77774.51) | 1294783(1236948,1350450) | 72390.95(69114.41,75555.62) | -0.08(-0.09,-0.07) | <0.001 |
| Kenya | 11647731(11263041,11995451) | 85059.55(82333.21,87528.57) | 20558296(19887320,21169784) | 83109.79(80341.28,85629.69) | -0.07(-0.07,-0.07) | <0.001 |
| Congo | 1151333(1112061,1189981) | 87136.23(84198.61,90025.78) | 2117548(2038473,2195171) | 84949.23(81764.72,88082.69) | -0.08(-0.09,-0.08) | <0.001 |
| Ukraine | 11490993(10725387,12218144) | 75839.94(70682.46,80733.32) | 6531673(6124503,6936336) | 75963.17(70944.05,80900.08) | 0.00(-0.01,0.01) | 0.80 |
| Liberia | 1147988(1103852,1190367) | 85291.42(82156.80,88302.25) | 2305962(2210371,2403882) | 82745.59(79330.13,86244.47) | -0.11(-0.12,-0.10) | <0.001 |
| United Kingdom | 11407487(10940267,11866298) | 76046.39(72827.99,79215.66) | 11828546(11396072,12262772) | 73996.49(71209.24,76798.59) | -0.06(-0.07,-0.05) | <0.001 |
| Nigeria | 41882634(40833659,42828520) | 87193.22(85094.29,89113.26) | 109948481(107220131,112498578) | 86365.54(84252.15,88342.02) | -0.04(-0.06,-0.02) | <0.001 |
| Paraguay | 1687419(1604490,1765824) | 82328.53(78400.35,86057.19) | 2257344(2168320,2338968) | 83733.06(80323.51,86875.27) | 0.05(0.02,0.07) | <0.001 |
| Puerto Rico | 1104576(1054725,1152277) | 82149.95(78325.99,85798.24) | 553188(530089,576260) | 82264.80(78468.40,86034.25) | 0.00(-0.01,0.01) | 0.95 |
| Costa Rica | 1094100(1030359,1154773) | 77629.09(73178.14,81871.03) | 1068490(1007288,1127692) | 76729.48(72139.63,81146.07) | -0.04(-0.04,-0.03) | <0.001 |
| Croatia | 1088676(1024377,1144828) | 80917.07(75992.00,85226.54) | 629989(589044,668909) | 76930.66(71757.09,81860.67) | -0.13(-0.18,-0.08) | <0.001 |
| Algeria | 10697309(10093138,11285679) | 79445.86(75030.44,83748.29) | 12795924(11998095,13556845) | 78483.26(73740.59,83005.89) | -0.04(-0.04,-0.03) | <0.001 |
| Maldives | 106581(101797,111167) | 84029.78(80417.94,87527.76) | 106956(101864,112058) | 81640.51(77683.53,85575.40) | -0.10(-0.10,-0.09) | <0.001 |
| Netherlands | 2851893(2711281,3007107) | 72507.88(68713.15,76733.55) | 2801992(2685194,2921185) | 74001.20(70808.42,77295.84) | 0.05(0.02,0.08) | <0.001 |
| Mexico | 32712259(30999364,34453158) | 75350.26(71379.91,79385.48) | 32388991(30755759,33992060) | 73912.62(70035.33,77711.53) | -0.05(-0.08,-0.02) | <0.001 |
| Armenia | 1026414(960174,1089741) | 77692.88(72732.69,82438.96) | 593663(555027,631214) | 77387.65(72310.79,82299.13) | -0.01(-0.01,0.00) | 0.16 |

Supplementary Table 4: Numbers and ASIR of non-communicable diseases in children and adolescents in 204 countries in 1990 and 2021, along with the Average Annual Percentage Change (AAPC) in ASIR from 1990 to 2021.

| **Location** | **1990-Number** | **1990-ASIR** | **2021-Number** | **2021-ASIR** | **AAPC(95%CI)** | ***P*** |
| --- | --- | --- | --- | --- | --- | --- |
| China | 541329945(432939120,685447283) | 124191.48(98538.73,158179.56) | 446196299(360446580,556098725) | 130883.62(106087.75,162581.93) | 0.17(0.12,0.21) | <0.001 |
| Democratic People's Republic of Korea | 9226621(7246604,11866448) | 120401.26(93983.14,155941.77) | 7862071(6114189,10247635) | 120294.52(93170.27,156402.90) | -0.02(-0.04,0.00) | 0.12 |
| Taiwan (Province of China) | 8938093(7007470,11575618) | 121815.07(95700.04,156298.45) | 5008239(3953227,6591184) | 123280.41(97189.37,162403.00) | 0.04(0.02,0.07) | <0.001 |
| Cambodia | 8727223(7066663,10884929) | 153986.14(124780.23,192796.83) | 10218507(8286953,12798202) | 153908.87(124819.47,192515.70) | 0.06(-0.02,0.13) | 0.14 |
| Indonesia | 138440024(112737024,171693157) | 157237.63(128232.74,194479.80) | 142735974(116333712,177098896) | 157658.51(128301.70,195429.27) | 0.02(-0.00,0.04) | 0.06 |
| Lao People's Democratic Republic | 3387273(2713503,4250665) | 150020.20(120254.22,188802.98) | 4474893(3613222,5632362) | 150429.87(121259.34,189739.15) | 0.00(-0.01,0.02) | 0.59 |
| Malaysia | 11284651(9152717,14355220) | 135538.25(109908.49,172588.64) | 14839043(11831535,18758769) | 144451.55(114900.68,182386.06) | 0.17(0.13,0.22) | <0.001 |
| Maldives | 192022(154229,241144) | 150405.22(120926.33,189835.32) | 198017(159083,249146) | 150411.62(120986.11,188651.08) | -0.01(-0.02,0.00) | 0.13 |
| Myanmar | 28470514(22841510,35385175) | 148785.63(119397.10,184686.00) | 30822422(24548418,38548699) | 148495.18(118051.34,185690.39) | 0.06(0.01,0.11) | 0.02 |
| Philippines | 50358173(41419302,62177256) | 157848.20(129812.84,195075.09) | 70348129(59446257,84766229) | 156628.55(132311.69,188390.64) | -0.03(-0.05,-0.02) | <0.001 |
| Sri Lanka | 10991375(8880221,13742914) | 150618.21(121777.33,187736.12) | 10477358(8556048,13308408) | 150313.45(122723.22,190314.14) | 0.03(0.01,0.04) | <0.001 |
| Thailand | 33304993(26968234,42122733) | 145757.36(118033.07,183377.77) | 20358444(16630137,25653929) | 148168.70(120869.59,185884.88) | 0.06(0.05,0.07) | <0.001 |
| Timor-Leste | 610441(494985,760502) | 151581.78(122715.07,190679.80) | 1024961(834213,1312447) | 150341.16(122165.32,192850.23) | -0.02(-0.06,0.01) | 0.14 |
| Viet Nam | 50511815(40491473,63407342) | 149682.39(120093.13,188077.35) | 47810672(38542548,60020131) | 149668.75(120964.40,187454.66) | 0.00(-0.01,0.02) | 0.75 |
| Fiji | 567775(456499,708904) | 156897.22(126385.61,195554.94) | 557987(453368,691610) | 158196.54(128658.19,195715.31) | 0.14(0.05,0.23) | <0.001 |
| Kiribati | 56486(45707,69746) | 155255.36(125550.21,192866.83) | 83448(67670,104053) | 155280.22(126118.57,193177.55) | -0.00(-0.02,0.01) | 0.54 |
| Marshall Islands | 42054(33981,52429) | 154014.39(125020.08,191560.20) | 35836(29213,45058) | 154958.70(126319.96,194341.41) | 0.02(0.02,0.03) | <0.001 |
| Micronesia (Federated States of) | 89047(71986,111888) | 153493.91(124430.33,192338.41) | 64178(52212,79727) | 154456.72(125540.19,191320.46) | 0.01(-0.00,0.03) | 0.11 |
| Papua New Guinea | 3260932(2638426,4061893) | 154145.06(124709.04,192445.88) | 7515071(6112546,9345291) | 154796.21(125845.12,193093.24) | 0.01(-0.00,0.01) | 0.18 |
| Samoa | 139992(114262,175183) | 153802.42(125499.42,192245.31) | 155477(126596,194403) | 154370.27(125746.10,193202.46) | 0.01(-0.00,0.02) | 0.14 |
| Solomon Islands | 297154(241242,372106) | 153914.72(124968.67,193095.95) | 506931(412509,627197) | 153966.54(125268.59,190633.96) | 0.00(-0.00,0.01) | 0.08 |
| Tonga | 81415(66148,101631) | 153624.09(124779.06,191760.97) | 76453(62025,95424) | 154900.68(125642.41,193668.08) | 0.02(0.02,0.03) | <0.001 |
| Vanuatu | 124622(100653,155700) | 150530.34(121665.33,188650.06) | 223654(180946,278570) | 151516.42(122668.09,188928.83) | 0.02(0.02,0.03) | <0.001 |
| Armenia | 1845912(1475849,2317826) | 139345.40(111360.80,175521.07) | 1113624(897412,1393700) | 144077.40(116418.54,179469.71) | 0.18(0.17,0.20) | <0.001 |
| Azerbaijan | 4294139(3422482,5372606) | 138125.39(109862.87,173545.58) | 4342742(3474726,5480360) | 140033.44(112359.38,175820.15) | 0.04(0.03,0.05) | <0.001 |
| Georgia | 2447725(1936076,3100474) | 136286.54(107670.40,172598.09) | 1261814(1018191,1608687) | 134187.55(108580.73,170598.51) | -0.06(-0.06,-0.05) | <0.001 |
| Kazakhstan | 9154405(7345483,11407711) | 138453.17(111084.59,172619.90) | 9408609(7473095,11826546) | 139758.69(111386.48,175391.39) | 0.03(0.03,0.04) | <0.001 |
| Kyrgyzstan | 2954463(2376931,3686527) | 141125.07(113471.27,176710.73) | 4064357(3252352,5113206) | 142779.97(114618.48,179402.23) | 0.03(0.03,0.04) | <0.001 |
| Mongolia | 1565721(1253619,1961877) | 139158.71(111397.97,174646.81) | 1863809(1485537,2335803) | 140286.83(112366.11,175943.32) | 0.03(0.03,0.04) | <0.001 |
| Tajikistan | 3908593(3137781,4887534) | 138104.92(110735.46,173863.32) | 6173922(4930833,7765865) | 138875.56(111040.28,175033.91) | 0.02(0.01,0.03) | <0.001 |
| Turkmenistan | 2580784(2061880,3226783) | 138462.86(110531.52,173893.92) | 2729828(2196226,3405336) | 139313.48(112058.61,173942.01) | 0.01(-0.00,0.02) | 0.06 |
| Uzbekistan | 14574031(11704038,18151410) | 138124.26(110852.02,172768.14) | 17441884(14038362,21944041) | 139189.30(112043.21,175710.72) | 0.03(0.02,0.03) | <0.001 |
| Albania | 1982000(1584732,2496112) | 137675.42(109934.43,173788.26) | 867425(703373,1087647) | 141036.51(113962.45,176836.27) | 0.09(0.07,0.10) | <0.001 |
| Bosnia and Herzegovina | 1965552(1577907,2523512) | 132617.88(106318.05,169952.26) | 943737(762749,1191695) | 141311.20(114208.67,177722.52) | 0.20(0.18,0.21) | <0.001 |
| Bulgaria | 3434094(2809672,4334501) | 145717.86(119385.10,182580.77) | 1903922(1537636,2391340) | 146512.21(118469.06,182791.43) | 0.07(0.03,0.10) | <0.001 |
| Croatia | 1804700(1467794,2306615) | 135399.97(110182.43,172196.60) | 1110516(933385,1319371) | 137708.49(115812.96,163048.36) | 0.07(0.03,0.10) | <0.001 |
| Czechia | 4327344(3471765,5467364) | 142778.28(114483.44,178693.63) | 3315517(2675737,4172639) | 149191.76(120677.52,186457.60) | 0.13(0.09,0.17) | <0.001 |
| Hungary | 3949278(3158061,5137526) | 135577.40(108526.91,174103.64) | 2661732(2144801,3329856) | 142169.38(114337.89,177620.11) | 0.19(0.17,0.21) | <0.001 |
| North Macedonia | 941422(762983,1213368) | 134635.99(109171.83,172963.99) | 637000(509928,799087) | 141750.37(113275.50,177489.16) | 0.20(0.18,0.22) | <0.001 |
| Montenegro | 291136(237333,366249) | 135440.19(110408.54,170007.20) | 213283(171677,266523) | 141977.76(114086.82,177136.80) | 0.16(0.14,0.19) | <0.001 |
| Poland | 18241961(14809640,22700469) | 144266.40(117452.29,178667.79) | 11162259(9083665,13894176) | 144428.33(117643.14,178703.53) | -0.00(-0.05,0.05) | 0.96 |
| Romania | 12595473(10795652,14269674) | 164068.32(140110.34,186707.60) | 6090256(4905871,7794408) | 150716.07(121727.93,191207.87) | -0.25(-0.28,-0.23) | <0.001 |
| Serbia | 3697544(2989011,4648575) | 127012.23(102628.89,159187.39) | 2377152(1903825,3019487) | 125610.27(100105.12,158966.48) | -0.01(-0.07,0.06) | 0.78 |
| Slovakia | 2488568(1992650,3149911) | 141056.37(113104.71,177382.97) | 1612538(1293231,2009948) | 143982.63(115507.29,178805.95) | 0.07(0.06,0.09) | <0.001 |
| Slovenia | 821010(668799,1033921) | 145931.26(118817.04,182623.08) | 605739(491192,757151) | 148332.97(120629.37,184345.86) | 0.04(0.01,0.08) | 0.02 |
| Belarus | 4554451(3669706,5811547) | 144327.81(116250.46,184189.92) | 3028806(2416543,3840704) | 145971.78(116938.15,184071.36) | 0.04(0.03,0.06) | <0.001 |
| Estonia | 640671(507487,822657) | 139621.21(110440.72,179428.00) | 396225(311448,511046) | 140816.28(111035.29,180154.66) | 0.01(-0.01,0.04) | 0.30 |
| Latvia | 1060382(855611,1328474) | 141341.71(113703.04,177914.88) | 555184(441555,699299) | 141649.40(112790.23,177931.48) | -0.00(-0.02,0.01) | 0.88 |
| Lithuania | 1609082(1257151,2063097) | 145629.54(113328.78,187390.01) | 820647(661167,1030586) | 151839.70(122402.91,190345.01) | 0.21(0.04,0.38) | 0.02 |
| Republic of Moldova | 2278611(1809516,2896671) | 143730.41(114220.59,182715.55) | 1015893(808233,1296555) | 143779.06(114565.88,182404.12) | 0.00(-0.01,0.01) | 0.80 |
| Russian Federation | 68066718(55294457,84610564) | 149767.81(121668.80,186155.61) | 52350715(43246515,63975450) | 151669.48(125795.24,184340.72) | 0.02(0.00,0.04) | 0.02 |
| Ukraine | 23024954(18708890,28534033) | 152622.48(123945.51,188995.18) | 13250946(10664836,16672073) | 153205.37(124111.69,190135.37) | 0.05(0.02,0.08) | <0.001 |
| Brunei Darussalam | 152157(119993,188480) | 133420.85(105195.72,165696.70) | 173463(137953,213633) | 133888.31(105947.33,165316.82) | 0.01(0.01,0.02) | <0.001 |
| Japan | 45060475(37768428,53250020) | 134064.97(111756.18,158882.81) | 26677593(22527060,30942733) | 123902.58(104244.73,144041.87) | -0.23(-0.27,-0.18) | <0.001 |
| Republic of Korea | 21753662(17114781,28536828) | 134349.29(105317.81,176464.34) | 12219001(9723096,15746856) | 141220.73(112320.45,180900.41) | 0.17(0.15,0.20) | <0.001 |
| Singapore | 1236258(994183,1502484) | 133739.11(106694.81,163211.13) | 1419723(1119521,1748085) | 134976.72(106420.16,166735.07) | 0.06(0.03,0.09) | <0.001 |
| Australia | 6397427(5884331,6938396) | 121250.28(111564.80,131471.89) | 9685881(7654808,11626310) | 151601.77(120102.87,181931.05) | 0.97(0.74,1.19) | <0.001 |
| New Zealand | 1472036(1202761,1772875) | 133027.88(107729.71,161238.81) | 1723413(1412817,2101479) | 129683.61(106177.05,158354.38) | -0.07(-0.13,-0.01) | 0.03 |
| Andorra | 18207(14598,22175) | 131337.84(104048.17,160897.34) | 19991(16094,24383) | 132943.23(105804.16,163045.45) | 0.06(0.03,0.09) | <0.001 |
| Austria | 2490995(2008161,3030811) | 130960.79(104650.16,160412.22) | 2353812(1875048,2880077) | 132634.19(104986.85,162984.40) | 0.03(0.01,0.04) | <0.001 |
| Belgium | 2744753(2235742,3364245) | 108306.53(87740.43,133515.09) | 3183081(2545985,3928835) | 122331.45(97477.64,151325.58) | 0.40(0.35,0.46) | <0.001 |
| Cyprus | 347098(277767,421975) | 132238.25(105711.74,160859.99) | 381564(304743,467818) | 133444.73(106428.75,163704.54) | 0.04(0.03,0.04) | <0.001 |
| Denmark | 1620953(1336966,1928572) | 128439.93(104497.73,154142.04) | 1659615(1376604,1996245) | 125705.84(103628.99,151993.12) | -0.08(-0.13,-0.04) | <0.001 |
| Finland | 1363423(1180499,1581667) | 105710.04(91649.39,122507.55) | 1514684(1197995,1856154) | 128390.86(101040.45,157605.64) | 0.58(0.55,0.61) | <0.001 |
| France | 19584371(16703597,23757333) | 118441.66(100487.57,144699.97) | 20554420(16730982,24943840) | 126669.78(102471.58,154404.04) | 0.28(0.24,0.32) | <0.001 |
| Germany | 22483102(18912089,27386739) | 128756.55(107922.17,157387.57) | 22512930(18676231,27332540) | 139721.17(115653.62,170060.43) | 0.28(0.25,0.31) | <0.001 |
| Greece | 3866893(3050762,5007355) | 130507.67(103134.08,168621.29) | 2418021(1946599,2925669) | 123014.20(98194.99,149452.67) | -0.29(-0.65,0.08) | 0.12 |
| Iceland | 111633(90633,136203) | 130994.47(105929.84,160277.02) | 120091(96251,146973) | 133408.99(106615.51,163404.58) | 0.07(0.05,0.09) | <0.001 |
| Ireland | 1795501(1424886,2252590) | 131470.47(103829.57,165316.74) | 1815924(1440031,2237559) | 134143.12(106005.77,165393.19) | 0.08(0.06,0.10) | <0.001 |
| Israel | 2847996(2315952,3565827) | 141105.01(114655.31,176664.48) | 4865663(3954962,6079433) | 144139.88(117210.13,180082.64) | 0.08(0.05,0.11) | <0.001 |
| Italy | 18183515(15261421,21273229) | 130182.15(108156.90,153157.54) | 13428650(11296002,15698340) | 124236.04(103942.12,145682.55) | -0.11(-0.15,-0.08) | <0.001 |
| Luxembourg | 115465(92625,140828) | 130389.98(104123.36,159520.97) | 179892(143272,220314) | 131689.65(104423.45,161630.10) | 0.03(0.03,0.04) | <0.001 |
| Malta | 149396(118613,184533) | 128092.81(101423.11,158361.04) | 109298(86736,135244) | 129732.36(102718.15,160760.70) | 0.04(0.03,0.05) | <0.001 |
| Netherlands | 5048407(4169217,6323300) | 130995.52(107031.84,165759.51) | 4861008(4123929,5683383) | 129452.51(109386.25,151909.51) | -0.08(-0.19,0.03) | 0.15 |
| Norway | 1501687(1226462,1797118) | 134070.61(108351.55,161639.70) | 1747055(1424040,2091291) | 137503.91(111485.33,165146.01) | 0.10(0.08,0.11) | <0.001 |
| Portugal | 3970242(3173140,4895435) | 128553.44(101751.00,159556.07) | 2556400(2034410,3154287) | 130804.25(102842.76,162757.53) | 0.07(0.04,0.10) | <0.001 |
| Spain | 17223829(14904122,19581220) | 141444.79(121859.59,161934.82) | 11333171(9070073,13919707) | 124053.19(98569.62,152784.10) | -0.43(-0.49,-0.38) | <0.001 |
| Sweden | 2955960(2411128,3713402) | 142377.36(115565.31,179435.88) | 3492128(2827428,4381642) | 142627.75(115271.71,178903.70) | 0.02(-0.00,0.05) | 0.10 |
| Switzerland | 2049157(1676830,2502352) | 129353.15(105185.93,158859.06) | 2239992(1814088,2752552) | 125346.82(101303.82,154310.69) | -0.10(-0.12,-0.07) | <0.001 |
| United Kingdom | 19888589(16356886,23695163) | 133607.55(109112.22,159970.45) | 19669300(16886163,22778407) | 122504.74(104918.05,142232.84) | -0.20(-0.23,-0.18) | <0.001 |
| Argentina | 17109674(13631259,21713897) | 130905.03(104371.30,165796.84) | 19216702(15378133,23832738) | 137274.46(109488.70,170425.51) | 0.18(0.13,0.23) | <0.001 |
| Chile | 7339224(5920535,9161700) | 140168.31(112639.55,175848.39) | 7133626(5760452,8912741) | 143492.28(115774.71,178661.51) | 0.07(0.03,0.11) | <0.001 |
| Uruguay | 1486944(1185392,1878331) | 137949.95(109820.21,173894.36) | 1287435(1030390,1613671) | 139806.74(111447.96,175500.72) | 0.06(0.05,0.07) | <0.001 |
| Canada | 8619459(6634792,11316284) | 111722.76(85665.28,147131.43) | 9728977(7567972,12272892) | 116533.67(90384.64,147164.20) | 0.14(0.11,0.16) | <0.001 |
| United States of America | 89851647(71622595,110469588) | 121303.67(96323.59,149601.01) | 104904529(86812369,124672168) | 127185.15(104699.12,151649.69) | 0.16(0.10,0.23) | <0.001 |
| Antigua and Barbuda | 38109(31250,47173) | 159163.05(130460.74,196839.36) | 37240(30569,45743) | 159862.83(130737.24,196008.24) | 0.02(0.01,0.03) | <0.001 |
| Bahamas | 172990(141969,211277) | 159776.11(131038.63,194926.16) | 185696(153493,227711) | 160701.37(132236.98,195684.96) | 0.02(0.00,0.04) | 0.02 |
| Barbados | 134910(110522,165916) | 158959.90(129904.61,195097.68) | 106256(87761,131007) | 160190.45(131803.61,196681.00) | 0.03(0.02,0.04) | <0.001 |
| Belize | 163090(134084,200213) | 158514.59(130594.16,194694.28) | 272068(222509,333473) | 160814.53(131143.08,196812.47) | 0.05(0.03,0.07) | <0.001 |
| Cuba | 5662907(4697455,6871420) | 158463.09(130222.39,193790.89) | 3570569(2950187,4203496) | 148047.38(122017.11,174432.25) | -0.43(-0.44,-0.42) | <0.001 |
| Dominica | 51333(42122,63529) | 157647.33(129121.53,195113.16) | 31263(25585,38617) | 159263.09(129919.79,195650.72) | 0.03(0.02,0.04) | <0.001 |
| Dominican Republic | 5437427(4457094,6694991) | 156844.20(128323.00,193424.90) | 6105141(5022837,7434010) | 157708.19(129471.67,192314.47) | 0.02(0.00,0.04) | 0.01 |
| Grenada | 66973(54613,82595) | 158613.65(129475.00,195684.73) | 47952(39473,58609) | 159762.81(131139.39,195144.29) | 0.03(0.02,0.04) | <0.001 |
| Guyana | 590337(482513,728700) | 157685.23(128498.83,194884.84) | 448334(367483,547321) | 160131.44(131041.19,195689.00) | 0.06(0.05,0.08) | <0.001 |
| Haiti | 5337400(4430804,6516982) | 160237.00(133080.12,196096.57) | 8441938(7021822,9916548) | 150916.30(125459.92,177363.50) | -0.30(-0.31,-0.29) | <0.001 |
| Jamaica | 1741431(1420189,2146580) | 159094.15(129730.97,195875.59) | 1303248(1068812,1607048) | 159731.95(130504.28,196343.85) | 0.01(0.01,0.02) | <0.001 |
| Saint Lucia | 106433(87115,132107) | 159301.21(130405.64,197452.42) | 64670(55504,75755) | 154122.67(132112.97,180369.67) | -0.21(-0.21,-0.20) | <0.001 |
| Saint Vincent and the Grenadines | 75886(62352,97231) | 140045.42(115213.83,178553.43) | 50515(41926,59596) | 148905.77(123306.67,175843.40) | 0.20(0.09,0.30) | <0.001 |
| Suriname | 269467(218542,335395) | 156966.82(127232.26,195542.80) | 291521(248285,340532) | 152646.93(130151.31,178004.12) | -0.12(-0.18,-0.07) | <0.001 |
| Trinidad and Tobago | 834615(679654,1021953) | 158520.22(129302.93,194007.74) | 554652(460536,648544) | 151158.91(125220.82,176886.89) | -0.17(-0.23,-0.11) | <0.001 |
| Bolivia (Plurinational State of) | 5303953(4353178,6425024) | 158694.40(130337.56,192410.04) | 7241349(5939337,8758570) | 159206.76(130495.65,192571.53) | 0.02(0.02,0.03) | <0.001 |
| Ecuador | 7644100(6364662,9283786) | 154096.37(128359.98,187012.39) | 10443103(9015908,11999258) | 157367.63(135904.65,180662.99) | 0.08(-0.00,0.17) | 0.06 |
| Peru | 17140102(14163420,20625594) | 161141.98(133174.38,193833.32) | 19960181(16458339,24248826) | 159814.58(131617.43,194275.61) | -0.02(-0.03,-0.01) | <0.001 |
| Colombia | 21262901(17037338,26550199) | 142017.45(113702.49,177414.82) | 18997815(15429109,22915717) | 130863.11(105605.31,158432.67) | -0.19(-0.25,-0.14) | <0.001 |
| Costa Rica | 2068689(1670983,2601440) | 145825.45(117926.26,183398.57) | 2035850(1635478,2555505) | 147296.09(118145.38,184608.04) | 0.04(0.03,0.04) | <0.001 |
| El Salvador | 4013213(3240155,5052095) | 146253.11(118118.68,183795.56) | 3484848(2800147,4396221) | 145332.53(116714.17,183373.94) | -0.02(-0.03,-0.01) | <0.001 |
| Guatemala | 7175466(5765042,9009746) | 145873.92(117628.38,183054.61) | 9676336(7762356,12159473) | 144970.12(116034.22,182353.31) | -0.01(-0.02,0.00) | 0.15 |
| Honduras | 3934938(3170124,4944301) | 144842.49(116980.81,181938.37) | 6048683(5110093,7139061) | 139045.32(117390.44,164107.37) | -0.13(-0.20,-0.06) | <0.001 |
| Mexico | 61468163(49562670,76105288) | 142424.50(114752.65,176236.60) | 63381094(53143084,75529083) | 146222.98(122560.64,173957.26) | 0.04(0.02,0.07) | <0.001 |
| Nicaragua | 3271166(2639472,4127600) | 144759.71(117097.94,182552.34) | 3471082(2821915,4158587) | 133017.88(107984.25,159497.98) | -0.27(-0.28,-0.26) | <0.001 |
| Panama | 1586642(1281907,1989146) | 145985.87(117879.66,182967.18) | 2075048(1700892,2461643) | 136673.62(111975.21,162172.07) | -0.20(-0.23,-0.17) | <0.001 |
| Venezuela (Bolivarian Republic of) | 13555092(10986634,16912372) | 149774.86(121414.73,186738.20) | 12003298(9865680,14207982) | 136985.48(112273.63,162443.11) | -0.30(-0.33,-0.28) | <0.001 |
| Brazil | 114997548(95704082,138328678) | 169679.72(141440.51,203706.20) | 111709462(92145320,137767216) | 175228.50(144324.68,216246.93) | 0.15(0.10,0.19) | <0.001 |
| Paraguay | 3399648(2764145,4238395) | 163552.72(133158.40,204180.02) | 4282832(3554733,5313965) | 160740.48(133393.45,198916.88) | -0.04(-0.05,-0.02) | <0.001 |
| Algeria | 17988580(14099563,22908850) | 131969.32(103666.89,168082.40) | 20069161(15967142,24324540) | 121248.82(97021.93,146474.46) | -0.28(-0.31,-0.25) | <0.001 |
| Bahrain | 272421(216471,338674) | 135681.97(108166.18,168686.17) | 553477(439935,694233) | 136334.74(108007.93,171305.44) | 0.01(0.00,0.02) | 0.01 |
| Egypt | 37926330(30149502,47728429) | 137411.76(109239.88,173186.31) | 58112599(46283920,70541013) | 124428.05(99408.79,150753.10) | -0.29(-0.31,-0.26) | <0.001 |
| Iran (Islamic Republic of) | 45056868(36391661,55971168) | 140623.02(114049.18,175038.72) | 37382865(30597855,46140675) | 141318.51(116023.92,173830.09) | 0.04(0.01,0.07) | 0.02 |
| Iraq | 13227972(10299764,16891904) | 129190.56(100715.75,165340.12) | 21224538(16843584,25723320) | 119524.25(94768.71,144887.66) | -0.21(-0.24,-0.19) | <0.001 |
| Jordan | 2601949(2095260,3221718) | 124879.00(100432.79,154902.34) | 6624359(5209933,8468517) | 133399.15(104556.85,170025.17) | 0.24(0.22,0.26) | <0.001 |
| Kuwait | 854704(698433,1050246) | 125179.61(102396.46,153544.31) | 1489656(1176447,1874077) | 134025.10(105930.55,168211.90) | 0.25(0.22,0.27) | <0.001 |
| Lebanon | 1747883(1371834,2216518) | 131728.81(103319.78,167319.19) | 2079614(1672163,2515998) | 123738.33(99457.44,149666.45) | -0.19(-0.21,-0.17) | <0.001 |
| Libya | 3092502(2438454,3889312) | 134151.03(105884.12,168424.75) | 2581570(2083434,3102876) | 123137.94(98534.68,148682.44) | -0.28(-0.30,-0.25) | <0.001 |
| Morocco | 16954102(13498945,21116493) | 136653.40(108865.27,170199.46) | 16394224(13257578,19595886) | 126427.76(101931.35,151427.51) | -0.24(-0.27,-0.22) | <0.001 |
| Palestine | 1552214(1224571,1947512) | 132002.22(104325.71,165757.88) | 2978170(2372954,3597667) | 122926.30(97984.48,148420.66) | -0.23(-0.30,-0.17) | <0.001 |
| Oman | 1299318(1044363,1637256) | 129654.65(104487.77,163430.55) | 2067748(1610584,2668961) | 136105.51(106659.89,175571.22) | 0.18(0.15,0.21) | <0.001 |
| Qatar | 201625(159736,254060) | 133864.87(106187.43,169023.56) | 820401(649993,1022983) | 137178.98(109169.10,170881.83) | 0.08(0.07,0.09) | <0.001 |
| Saudi Arabia | 12872388(10786420,14933501) | 158949.14(133341.20,184121.42) | 13642437(11089859,17256355) | 133515.75(108418.68,169015.77) | -0.56(-0.62,-0.50) | <0.001 |
| Syrian Arab Republic | 9810121(7696853,12383453) | 132955.53(104610.62,167834.19) | 6608850(5420806,7854992) | 122739.75(99130.71,147170.54) | -0.20(-0.31,-0.08) | <0.001 |
| Tunisia | 5310817(4169831,6783525) | 132643.77(104215.58,169287.55) | 4924015(3877689,6320743) | 135686.05(106906.73,173840.25) | 0.09(0.08,0.10) | <0.001 |
| Turkey | 36544334(29165342,46815685) | 137150.95(109426.74,175237.73) | 35063454(27776086,44592847) | 139717.95(110552.55,177303.60) | 0.07(0.06,0.09) | <0.001 |
| United Arab Emirates | 962800(763608,1200996) | 134684.21(107197.73,168683.38) | 2117130(1711526,2561815) | 123158.21(100015.82,148555.63) | -0.30(-0.34,-0.26) | <0.001 |
| Yemen | 10773338(8357772,13746427) | 127695.26(99697.15,163007.47) | 20588348(16386611,25164617) | 117499.89(93681.19,143445.44) | -0.29(-0.31,-0.26) | <0.001 |
| Afghanistan | 7117089(5651233,9002942) | 131231.93(103897.87,166525.35) | 21307280(17105540,25809542) | 120701.72(97111.51,146037.98) | -0.26(-0.29,-0.23) | <0.001 |
| Bangladesh | 92923639(75083825,116205494) | 154275.27(124668.22,193879.16) | 94215313(75589050,118478680) | 153722.58(123344.69,192600.27) | -0.02(-0.03,-0.01) | <0.001 |
| Bhutan | 514357(417106,644335) | 152062.04(123090.96,190783.20) | 345403(286126,412444) | 135528.68(111737.60,162339.00) | -0.38(-0.40,-0.37) | <0.001 |
| India | 672880456(558544286,817870820) | 163037.74(135511.99,198363.93) | 783826313(676213489,915426593) | 156143.22(134636.62,182066.47) | -0.13(-0.19,-0.07) | <0.001 |
| Nepal | 15485323(12673782,19542619) | 150566.98(123144.25,190591.80) | 17077961(14331060,20349399) | 137364.55(114903.49,164018.12) | -0.26(-0.34,-0.19) | <0.001 |
| Pakistan | 99152681(81498638,122278730) | 162793.16(133870.77,201148.82) | 166901720(141684985,195300272) | 152641.42(129659.07,178524.97) | -0.14(-0.24,-0.03) | 0.01 |
| Angola | 9969856(8257863,11974645) | 173672.49(143699.67,208956.99) | 29154230(24235511,34469190) | 155737.41(130017.07,183557.26) | -0.48(-0.58,-0.37) | <0.001 |
| Central African Republic | 2598663(2143399,3144973) | 173974.74(143351.79,211248.98) | 4541711(3787100,5350596) | 157137.42(131092.58,185046.29) | -0.33(-0.38,-0.28) | <0.001 |
| Congo | 2297109(1901789,2752063) | 173497.70(143665.04,207873.46) | 4277486(3533650,5153418) | 171323.73(141720.21,206063.00) | -0.03(-0.07,0.00) | 0.07 |
| Democratic Republic of the Congo | 37343650(30939002,44675384) | 172875.97(143079.97,207258.86) | 74263815(61662765,87606562) | 154833.24(128796.19,182396.89) | -0.39(-0.43,-0.35) | <0.001 |
| Equatorial Guinea | 412741(341228,494733) | 172729.39(142782.52,207375.82) | 1187215(992142,1399534) | 153977.97(128494.42,181801.87) | -0.36(-0.40,-0.32) | <0.001 |
| Gabon | 877510(735453,1047070) | 172819.83(144857.05,206513.26) | 1278298(1074997,1516222) | 154344.67(129852.61,183000.77) | -0.34(-0.42,-0.26) | <0.001 |
| Burundi | 5650851(4675818,6795112) | 178827.76(148068.26,215664.69) | 11726053(9897451,13788131) | 160997.23(136202.16,188990.45) | -0.30(-0.34,-0.26) | <0.001 |
| Comoros | 470100(388210,564999) | 178756.09(147612.90,214926.08) | 562893(466069,676632) | 179238.19(148364.37,215366.62) | 0.02(0.01,0.03) | <0.001 |
| Djibouti | 397334(329703,476965) | 178523.86(147933.81,214460.49) | 858005(717630,1002138) | 162416.76(135898.82,189629.69) | -0.29(-0.33,-0.25) | <0.001 |
| Eritrea | 3486108(2894002,4219398) | 177536.57(147416.07,215221.77) | 5214298(4402937,6104649) | 162125.17(137002.31,189706.25) | -0.33(-0.36,-0.29) | <0.001 |
| Ethiopia | 63278016(53813470,74988406) | 214032.34(182328.29,253525.73) | 119387136(101774984,141258650) | 209591.61(178569.55,247983.52) | -0.08(-0.11,-0.06) | <0.001 |
| Kenya | 24774941(20896460,29581129) | 179877.52(151884.36,214732.98) | 44198043(37851925,51617406) | 179338.93(153555.78,209244.96) | -0.01(-0.03,0.01) | 0.38 |
| Madagascar | 13453778(11883527,15206026) | 202148.47(178694.49,228160.70) | 23994703(20426474,28073248) | 160252.22(136543.58,187336.65) | -0.80(-0.87,-0.74) | <0.001 |
| Malawi | 9735317(8093886,11774717) | 176038.49(146147.06,213664.45) | 18623247(15428558,22784396) | 175967.12(145766.12,214895.03) | 0.00(-0.00,0.01) | 0.33 |
| Mauritius | 650218(522583,815366) | 150543.68(121133.73,187649.65) | 409832(339970,487515) | 137653.43(113059.88,164807.20) | -0.30(-0.34,-0.26) | <0.001 |
| Mozambique | 13096770(10868968,15931016) | 172495.45(143382.39,210053.66) | 28258736(23775322,33357499) | 158642.68(133909.96,186815.53) | -0.42(-0.43,-0.40) | <0.001 |
| Rwanda | 7613858(6320993,9283623) | 183137.79(152130.82,223985.12) | 10516308(8838586,12302020) | 163599.43(137409.29,191462.17) | -0.32(-0.37,-0.27) | <0.001 |
| Seychelles | 46830(37783,58924) | 150010.84(120899.77,188624.65) | 46037(37131,57056) | 150552.94(121401.08,186331.36) | 0.01(0.01,0.02) | <0.001 |
| Somalia | 8290673(6904558,10056228) | 175476.52(146248.41,212865.87) | 20486616(17320463,23996164) | 160485.50(136018.13,187718.11) | -0.28(-0.30,-0.26) | <0.001 |
| United Republic of Tanzania | 22800016(19685771,26821807) | 153424.11(132423.39,180425.16) | 49343986(41577761,57824468) | 159729.77(134722.09,187047.02) | 0.08(-0.03,0.19) | 0.14 |
| Uganda | 17542143(14609013,21210847) | 172022.98(142813.47,208827.82) | 43306456(35754654,52829715) | 173754.24(143502.55,212001.97) | 0.09(0.05,0.13) | <0.001 |
| Zambia | 8400920(7036358,10048696) | 180622.99(151051.43,216401.52) | 18907081(15643044,22778609) | 180426.80(149451.78,217229.60) | -0.01(-0.02,0.00) | 0.17 |
| Botswana | 1123391(916200,1377761) | 151810.18(124014.42,185932.98) | 1403326(1148057,1712986) | 152969.84(125037.29,186678.54) | 0.03(-0.00,0.07) | 0.05 |
| Lesotho | 1264846(1022583,1568143) | 150791.13(122419.02,186419.57) | 1184595(989288,1413313) | 140754.55(117184.14,168247.75) | -0.24(-0.26,-0.21) | <0.001 |
| Namibia | 1131474(925274,1391633) | 149439.28(122157.52,184000.69) | 1499221(1250578,1784005) | 139852.40(116541.21,166519.96) | -0.22(-0.24,-0.20) | <0.001 |
| South Africa | 27407691(22847718,32797888) | 155975.49(129916.13,186783.83) | 32206746(27239239,37944612) | 160941.92(136048.84,189519.11) | 0.10(0.09,0.12) | <0.001 |
| Eswatini | 724226(586838,893999) | 152070.02(123453.74,187770.96) | 829156(676398,1026344) | 154796.05(126307.18,191366.94) | 0.07(0.05,0.09) | <0.001 |
| Zimbabwe | 8644421(7043904,10897784) | 143908.81(117418.70,181215.13) | 11919739(9706257,14379755) | 149116.44(121584.67,179723.63) | 0.12(0.08,0.15) | <0.001 |
| Benin | 4773976(3889198,5834982) | 165916.81(135672.17,203312.09) | 12306013(10094765,14859900) | 164126.19(134865.58,198154.93) | -0.03(-0.05,-0.02) | <0.001 |
| Burkina Faso | 9366928(7739195,11315931) | 165240.92(136760.87,199770.52) | 19198575(16027870,22616760) | 150123.27(125647.03,176660.11) | -0.27(-0.39,-0.15) | <0.001 |
| Cameroon | 9463665(7784571,11410035) | 160640.97(132330.99,193832.83) | 26963557(22181948,32363331) | 159433.75(131410.13,191224.92) | -0.03(-0.06,-0.01) | 0.01 |
| Cabo Verde | 313978(258172,379953) | 161500.27(133096.67,195488.23) | 313091(257435,378796) | 161564.01(132563.01,195371.49) | 0.00(-0.01,0.01) | 0.98 |
| Chad | 5749375(4722659,7055603) | 164802.09(135445.04,203098.99) | 17854173(14579971,21641525) | 163988.79(134198.21,198876.77) | -0.02(-0.03,-0.00) | 0.03 |
| C么te d'Ivoire | 11277354(9274527,13574712) | 163527.31(134746.65,197166.77) | 23647192(19457894,28405295) | 164530.51(135608.57,197637.82) | 0.03(0.01,0.05) | <0.001 |
| Gambia | 918102(755229,1114905) | 164329.31(135409.41,199830.42) | 1970606(1682374,2282762) | 155897.44(133111.47,180567.07) | -0.31(-0.34,-0.28) | <0.001 |
| Ghana | 12830834(10390988,15512049) | 155342.50(126034.99,187969.62) | 25198525(20574948,30539117) | 154323.11(126141.33,187028.90) | -0.02(-0.04,-0.01) | <0.001 |
| Guinea | 5406786(4421055,6547073) | 165277.67(135433.61,200533.64) | 12330897(10107887,14916117) | 165006.35(135602.85,199481.01) | -0.02(-0.03,-0.00) | 0.01 |
| Guinea-Bissau | 982228(808716,1188351) | 166893.56(137697.65,201992.78) | 1856132(1519880,2253033) | 165898.13(136095.35,201311.15) | -0.01(-0.02,0.01) | 0.45 |
| Liberia | 2262809(1853714,2736281) | 165915.33(136134.72,201122.66) | 4556746(3747485,5516936) | 163370.42(134449.78,197582.67) | -0.04(-0.07,-0.01) | 0.02 |
| Mali | 8576991(7100455,10325780) | 174163.28(144344.90,210082.48) | 24775833(20458803,29601773) | 174793.59(144503.14,208875.74) | -0.00(-0.02,0.01) | 0.72 |
| Mauritania | 1870249(1529123,2278018) | 165799.81(135715.99,202289.95) | 3804976(3123896,4604744) | 162786.40(133908.61,196802.75) | -0.04(-0.05,-0.04) | <0.001 |
| Niger | 9800969(8368723,11146444) | 206874.70(177613.13,234359.47) | 24963375(21587273,28869821) | 160476.66(138699.58,186004.97) | -0.78(-0.87,-0.69) | <0.001 |
| Nigeria | 86186083(72851210,101274428) | 179691.52(151974.94,211192.34) | 226207578(195734923,259703673) | 177214.15(153452.07,203319.09) | -0.07(-0.12,-0.02) | <0.001 |
| Sao Tome and Principe | 115601(94077,142187) | 164735.57(134497.43,202285.92) | 150936(126623,178393) | 148077.66(124078.63,175152.54) | -0.61(-0.62,-0.59) | <0.001 |
| Senegal | 7511140(6148664,9098151) | 170044.15(139476.47,206142.61) | 13631993(11206754,16406863) | 168279.74(138401.69,202426.55) | -0.04(-0.05,-0.03) | <0.001 |
| Sierra Leone | 3647164(3033038,4515920) | 166753.67(138347.80,208142.60) | 7088515(5904564,8220325) | 156251.41(130219.34,181125.44) | -0.46(-0.49,-0.44) | <0.001 |
| Togo | 3594454(2991130,4292437) | 167178.52(139533.14,199475.08) | 6424873(5360003,7530485) | 153863.31(128635.52,180047.20) | -0.54(-0.58,-0.49) | <0.001 |
| American Samoa | 36441(29637,45509) | 154788.52(125682.78,194221.68) | 27998(22936,33325) | 144748.62(118345.16,172228.55) | -0.35(-0.50,-0.20) | <0.001 |
| Bermuda | 25158(20599,30883) | 159667.58(130273.91,196621.38) | 18565(15196,22865) | 161075.65(131547.40,197592.72) | 0.03(0.01,0.04) | <0.001 |
| Cook Islands | 13270(10725,16663) | 154073.49(124467.21,193260.43) | 7800(6608,9266) | 150099.86(127245.13,177712.52) | -0.10(-0.15,-0.04) | <0.001 |
| Greenland | 21035(16300,26528) | 119033.12(92366.61,150291.27) | 18647(14480,23574) | 121059.53(93845.22,153119.10) | 0.07(0.04,0.10) | <0.001 |
| Guam | 82704(67667,102581) | 155099.35(126543.95,193354.43) | 72587(61633,85653) | 151044.35(128099.50,178198.15) | -0.10(-0.14,-0.05) | <0.001 |
| Monaco | 6448(5156,7872) | 131512.75(104340.97,161418.55) | 9166(7318,11154) | 133235.28(105669.57,162871.39) | 0.05(0.04,0.06) | <0.001 |
| Nauru | 8067(6497,10013) | 155173.24(125119.26,193375.04) | 7663(6478,9030) | 149555.60(126409.55,176231.30) | -0.22(-0.22,-0.22) | <0.001 |
| Niue | 1610(1306,1999) | 153828.60(125124.76,190749.32) | 823(671,1031) | 155236.11(126270.67,193882.44) | 0.02(0.01,0.04) | <0.001 |
| Northern Mariana Islands | 24622(20062,30368) | 155567.07(126109.73,192876.28) | 23689(19159,29556) | 156352.85(126541.52,193576.96) | -0.02(-0.05,0.02) | 0.43 |
| Palau | 9492(7733,11878) | 154208.64(125432.23,192721.76) | 6896(5603,8648) | 154682.22(125558.93,192973.08) | 0.02(0.00,0.04) | 0.04 |
| Puerto Rico | 2122833(1741135,2637041) | 159558.59(130748.82,197532.07) | 983260(818834,1159140) | 150554.08(124151.64,178356.37) | -0.20(-0.21,-0.18) | <0.001 |
| Saint Kitts and Nevis | 29271(24003,35808) | 158624.86(130108.28,193794.00) | 22345(18369,27332) | 159536.57(130342.41,195400.76) | 0.02(0.01,0.04) | 0.01 |
| San Marino | 8050(6494,9835) | 130826.66(103916.95,161450.96) | 8169(6888,9564) | 125890.01(105539.34,148005.81) | -0.13(-0.22,-0.05) | <0.001 |
| Tokelau | 1197(966,1490) | 153487.55(124325.10,191506.02) | 786(658,939) | 149582.65(125732.21,177497.65) | -0.20(-0.25,-0.14) | <0.001 |
| Tuvalu | 6421(5247,7971) | 153784.25(125292.32,192589.33) | 7551(6136,9450) | 154106.93(125078.87,192898.54) | 0.01(0.00,0.02) | <0.001 |
| United States Virgin Islands | 66155(54286,81587) | 159344.87(130666.08,196062.06) | 29492(24219,36284) | 160980.78(132000.58,197090.40) | 0.03(0.02,0.04) | <0.001 |
| South Sudan | 5764359(4794230,6986116) | 176246.50(146499.98,213949.74) | 9544509(7975476,11581483) | 174975.60(146150.53,212087.00) | -0.03(-0.05,-0.01) | <0.001 |
| Sudan | 12685592(10157174,16086367) | 116961.56(93591.07,148296.83) | 25844190(20638139,31276482) | 120688.97(96370.40,146035.81) | 0.11(0.07,0.15) | <0.001 |

Supplementary Table 5：Numbers and ASMR of non-communicable diseases in children and adolescents in 204 countries in 1990 and 2021, along with the Average Annual Percentage Change (AAPC) in ASMR from 1990 to 2021.

| **Location** | **1990-Number** | **1990-ASMR** | **2021-Number** | **2021-ASMR** | **AAPC(95%CI)** | ***P*** |
| --- | --- | --- | --- | --- | --- | --- |
| Afghanistan | 14983(6513,19954) | 242.77(106.77,323.60) | 21929(14747,28024) | 112.81(76.40,143.84) | -2.36(-2.57,-2.15) | <0.001 |
| Albania | 780(656,912) | 52.99(44.60,61.95) | 147(113,188) | 26.39(20.20,34.01) | -2.65(-2.94,-2.36) | <0.001 |
| Algeria | 16769(10865,20574) | 122.30(79.34,150.02) | 7434(5989,9238) | 43.68(35.22,54.22) | -3.32(-3.42,-3.22) | <0.001 |
| American Samoa | 7(6,9) | 29.15(24.33,34.65) | 4(3,6) | 25.03(19.02,32.24) | -0.30(-0.55,-0.06) | 0.02 |
| Andorra | 3(2,4) | 24.83(18.51,31.62) | 1(1,1) | 6.70(4.94,8.52) | -4.04(-4.73,-3.35) | <0.001 |
| Angola | 7425(3914,11597) | 109.02(58.72,168.68) | 9054(6612,12269) | 45.85(33.40,62.10) | -2.77(-2.88,-2.66) | <0.001 |
| Antigua and Barbuda | 7(6,8) | 28.51(24.04,33.41) | 6(5,7) | 26.56(23.15,30.18) | -0.44(-0.76,-0.12) | 0.01 |
| Argentina | 6603(6278,6926) | 51.74(49.20,54.27) | 3183(2668,3767) | 26.21(21.74,31.31) | -2.17(-2.36,-1.98) | <0.001 |
| Armenia | 653(580,742) | 47.08(41.91,53.48) | 158(130,191) | 22.04(18.11,26.79) | -2.36(-2.82,-1.90) | <0.001 |
| Australia | 1575(1506,1652) | 32.36(30.93,33.94) | 646(565,739) | 10.88(9.46,12.49) | -3.11(-3.53,-2.70) | <0.001 |
| Austria | 606(572,639) | 34.99(33.05,36.92) | 195(173,218) | 11.47(10.15,12.90) | -3.49(-4.07,-2.92) | <0.001 |
| Azerbaijan | 1985(1682,2331) | 61.01(51.75,71.57) | 1034(811,1345) | 36.29(28.25,47.58) | -1.89(-2.18,-1.60) | <0.001 |
| Bahamas | 44(37,51) | 70.89(61.36,78.96) | 28(22,36) | 23.53(20.04,27.61) | -1.46(-1.81,-1.11) | <0.001 |
| Bahrain | 153(132,170) | 98.15(65.75,130.00) | 90(77,105) | 33.32(22.98,47.21) | -3.66(-3.97,-3.35) | <0.001 |
| Bangladesh | 65074(43300,86513) | 41.93(36.00,48.09) | 19271(13478,26997) | 30.32(21.81,41.75) | -3.47(-3.60,-3.34) | <0.001 |
| Barbados | 33(29,38) | 42.28(36.08,48.62) | 18(13,24) | 12.40(9.99,15.73) | -1.03(-2.51,0.46) | 0.17 |
| Belarus | 1281(1094,1472) | 34.08(32.39,35.97) | 235(191,295) | 13.14(11.29,15.35) | -4.14(-4.62,-3.66) | <0.001 |
| Belgium | 784(745,827) | 63.92(57.30,71.61) | 312(270,363) | 28.56(23.60,34.64) | -3.29(-3.88,-2.69) | <0.001 |
| Belize | 68(61,77) | 121.27(63.80,164.08) | 44(37,53) | 72.81(54.33,95.85) | -2.59(-2.95,-2.24) | <0.001 |
| Benin | 4126(2103,5609) | 27.14(22.90,32.76) | 5973(4455,7869) | 14.98(11.89,19.48) | -1.73(-1.93,-1.53) | <0.001 |
| Bermuda | 4(4,5) | 70.43(36.81,101.94) | 2(1,2) | 32.71(21.50,45.43) | -1.95(-2.30,-1.61) | <0.001 |
| Bhutan | 246(129,356) | 167.70(107.49,212.47) | 79(52,109) | 63.44(50.32,79.46) | -2.52(-2.91,-2.12) | <0.001 |
| Bolivia (Plurinational State of) | 6089(3874,7729) | 24.35(20.71,28.42) | 2822(2238,3537) | 12.15(10.10,14.70) | -3.06(-3.10,-3.01) | <0.001 |
| Bosnia and Herzegovina | 338(288,393) | 34.46(25.63,44.35) | 76(63,92) | 30.36(21.67,40.96) | -2.33(-2.99,-1.68) | <0.001 |
| Botswana | 261(194,335) | 46.03(40.06,51.66) | 270(193,364) | 29.50(24.55,34.86) | -0.43(-0.57,-0.29) | <0.001 |
| Brazil | 28894(25194,32366) | 43.16(37.25,49.39) | 18446(15402,21740) | 30.39(24.26,37.52) | -1.57(-1.73,-1.42) | <0.001 |
| Brunei Darussalam | 52(45,60) | 46.89(43.39,50.05) | 36(29,44) | 18.86(15.74,22.08) | -1.20(-1.47,-0.92) | <0.001 |
| Bulgaria | 1001(926,1068) | 130.22(74.15,193.80) | 230(193,268) | 91.58(61.36,145.72) | -2.91(-3.17,-2.66) | <0.001 |
| Burkina Faso | 8592(4874,12707) | 112.42(69.21,154.20) | 13212(8872,21009) | 51.41(37.76,68.74) | -1.10(-1.24,-0.95) | <0.001 |
| Burundi | 4100(2465,5673) | 124.94(64.49,168.46) | 3895(2855,5219) | 54.52(40.81,71.30) | -2.60(-2.74,-2.46) | <0.001 |
| Cabo Verde | 112(78,144) | 86.45(51.05,109.66) | 41(31,53) | 58.55(42.14,75.92) | -2.64(-2.88,-2.40) | <0.001 |
| Cambodia | 7994(4013,10834) | 29.38(28.17,30.60) | 3552(2658,4646) | 12.52(10.85,14.36) | -2.65(-2.75,-2.54) | <0.001 |
| Cameroon | 5966(3442,7590) | 52.95(36.99,67.82) | 10200(7349,13209) | 22.44(16.80,29.27) | -1.24(-1.46,-1.03) | <0.001 |
| Canada | 2145(2057,2234) | 113.13(70.31,168.20) | 966(843,1101) | 77.08(53.10,110.91) | -2.71(-3.04,-2.37) | <0.001 |
| Central African Republic | 1980(1211,2960) | 88.83(47.87,125.59) | 2323(1599,3345) | 72.16(42.97,98.33) | -1.27(-1.40,-1.13) | <0.001 |
| Chad | 3803(1996,5404) | 40.57(38.68,42.55) | 8997(5263,12304) | 17.76(15.54,20.06) | -0.66(-0.78,-0.54) | <0.001 |
| Chile | 2151(2050,2256) | 77.99(61.41,95.63) | 786(693,882) | 16.21(13.37,19.79) | -2.73(-3.05,-2.40) | <0.001 |
| China | 331011(261657,404778) | 47.64(42.28,52.97) | 50642(41935,61556) | 27.68(21.02,36.44) | -5.13(-5.30,-4.96) | <0.001 |
| Colombia | 7296(6472,8114) | 96.69(53.96,142.72) | 3798(2913,4951) | 54.18(38.58,76.86) | -2.16(-2.43,-1.89) | <0.001 |
| Comoros | 282(159,418) | 71.44(48.34,99.74) | 166(118,235) | 36.42(27.61,47.31) | -1.93(-2.70,-1.16) | <0.001 |
| Congo | 1007(679,1409) | 21.96(16.46,28.34) | 874(663,1135) | 10.76(7.92,15.61) | -2.23(-2.50,-1.95) | <0.001 |
| Cook Islands | 2(1,2) | 39.22(36.71,41.61) | 1(0,1) | 25.80(22.05,30.34) | -0.83(-1.85,0.20) | 0.11 |
| Costa Rica | 576(539,611) | 85.39(52.78,108.83) | 323(278,377) | 57.19(44.20,73.31) | -1.43(-1.83,-1.02) | <0.001 |
| Croatia | 339(317,362) | 28.26(26.46,30.22) | 94(78,114) | 12.75(10.49,15.50) | -2.46(-2.93,-1.98) | <0.001 |
| Cuba | 1261(1194,1329) | 35.53(33.62,37.48) | 357(307,413) | 15.64(13.36,18.15) | -2.28(-2.98,-1.58) | <0.001 |
| Cyprus | 72(55,84) | 29.57(22.60,34.86) | 21(17,25) | 7.31(5.96,8.88) | -5.01(-5.42,-4.60) | <0.001 |
| Czechia | 785(739,830) | 29.36(27.59,31.08) | 165(140,193) | 7.58(6.41,8.89) | -4.13(-4.41,-3.84) | <0.001 |
| C么te d'Ivoire | 6838(4162,8716) | 94.46(62.85,131.78) | 8764(6796,11221) | 42.05(30.33,58.70) | -1.35(-1.61,-1.10) | <0.001 |
| Democratic People's Republic of Korea | 4456(3276,5881) | 33.79(31.65,36.25) | 1493(1090,2055) | 10.89(9.34,12.45) | -2.58(-2.68,-2.49) | <0.001 |
| Democratic Republic of the Congo | 24038(15861,33585) | 69.71(44.20,102.74) | 20513(14795,28616) | 40.36(26.56,59.75) | -2.63(-2.72,-2.55) | <0.001 |
| Denmark | 380(356,408) | 36.68(30.13,43.49) | 134(115,152) | 57.08(43.11,73.95) | -3.51(-4.03,-2.99) | <0.001 |
| Djibouti | 163(103,241) | 83.08(70.81,96.26) | 215(141,318) | 37.40(27.42,54.28) | -1.58(-1.97,-1.19) | <0.001 |
| Dominica | 12(10,14) | 70.59(64.50,76.91) | 9(7,11) | 34.24(26.79,43.45) | 1.40(1.18,1.61) | <0.001 |
| Dominican Republic | 3008(2564,3486) | 191.45(131.27,231.10) | 1444(1061,2091) | 45.49(36.65,55.90) | -2.43(-2.67,-2.19) | <0.001 |
| Ecuador | 3497(3195,3810) | 85.68(67.83,101.63) | 2172(1705,2748) | 26.33(19.23,35.29) | -2.56(-2.92,-2.19) | <0.001 |
| Egypt | 58299(39638,70486) | 87.79(59.13,123.81) | 21428(17252,26350) | 34.35(21.49,55.65) | -4.48(-4.81,-4.14) | <0.001 |
| El Salvador | 2409(1906,2859) | 87.03(51.41,130.23) | 611(448,815) | 56.03(38.41,81.72) | -3.80(-4.07,-3.53) | <0.001 |
| Equatorial Guinea | 247(165,351) | 38.55(35.89,41.37) | 250(156,408) | 11.08(9.56,12.66) | -2.85(-3.14,-2.56) | <0.001 |
| Eritrea | 1886(1095,2850) | 118.89(65.63,182.78) | 1852(1267,2708) | 49.77(36.76,69.64) | -1.45(-1.61,-1.29) | <0.001 |
| Estonia | 175(163,187) | 55.13(36.33,70.71) | 31(26,35) | 29.35(22.17,37.86) | -3.81(-4.30,-3.32) | <0.001 |
| Eswatini | 216(156,266) | 45.22(36.00,56.02) | 196(138,264) | 43.84(32.72,56.95) | -0.40(-0.51,-0.30) | <0.001 |
| Ethiopia | 39431(21092,61557) | 25.33(23.61,27.20) | 28948(21366,40560) | 11.29(9.99,12.78) | -2.80(-2.97,-2.64) | <0.001 |
| Fiji | 159(127,197) | 30.61(29.45,31.75) | 151(113,196) | 11.52(10.03,13.03) | -0.11(-0.28,0.06) | 0.19 |
| Finland | 304(284,326) | 54.35(39.08,75.52) | 122(109,138) | 32.36(22.10,47.06) | -2.45(-2.94,-1.97) | <0.001 |
| France | 4588(4413,4760) | 28.81(25.24,32.59) | 1651(1450,1856) | 19.29(15.75,23.37) | -3.00(-3.39,-2.61) | <0.001 |
| Gabon | 298(214,417) | 32.11(30.93,33.32) | 261(178,379) | 12.08(10.84,13.30) | -1.73(-1.91,-1.55) | <0.001 |
| Gambia | 470(303,681) | 84.92(59.85,106.75) | 600(441,881) | 49.43(35.55,68.08) | -1.42(-2.06,-0.78) | <0.001 |
| Georgia | 510(448,576) | 31.22(29.35,33.06) | 173(142,210) | 12.71(10.95,14.55) | -1.25(-1.76,-0.73) | <0.001 |
| Germany | 5419(5219,5626) | 56.83(45.04,70.96) | 1875(1686,2061) | 20.01(15.62,26.42) | -2.94(-3.23,-2.65) | <0.001 |
| Ghana | 7705(5403,9690) | 44.80(37.56,53.20) | 8216(5902,11329) | 31.05(25.38,37.69) | -1.84(-2.00,-1.69) | <0.001 |
| Greece | 723(680,764) | 26.20(22.43,30.36) | 223(194,254) | 20.64(16.16,25.78) | -2.86(-3.31,-2.41) | <0.001 |
| Greenland | 11(9,14) | 73.42(66.93,80.22) | 3(2,4) | 41.52(33.17,52.15) | -3.09(-3.28,-2.91) | <0.001 |
| Grenada | 19(16,23) | 143.46(75.32,192.75) | 9(7,11) | 76.24(56.07,97.31) | -0.99(-1.20,-0.78) | <0.001 |
| Guam | 15(13,17) | 128.58(76.20,179.37) | 10(8,12) | 59.51(43.59,86.23) | -1.04(-1.56,-0.52) | <0.001 |
| Guatemala | 3917(3564,4286) | 60.60(50.67,70.23) | 2547(2046,3182) | 40.27(30.20,52.77) | -1.67(-2.14,-1.20) | <0.001 |
| Guinea | 5800(2975,7805) | 194.84(150.89,238.19) | 6063(4459,7729) | 116.66(86.14,156.08) | -2.00(-2.22,-1.77) | <0.001 |
| Guinea-Bissau | 839(490,1170) | 105.62(89.71,123.45) | 691(506,999) | 35.14(24.98,47.93) | -2.43(-2.62,-2.24) | <0.001 |
| Guyana | 243(204,282) | 38.81(36.67,41.02) | 112(84,147) | 11.68(9.65,13.92) | -1.30(-1.68,-0.92) | <0.001 |
| Haiti | 7311(5656,8926) | 26.06(23.61,28.61) | 6680(4932,8937) | 10.48(8.92,12.23) | -1.55(-1.71,-1.39) | <0.001 |
| Honduras | 3094(2626,3619) | 60.82(45.49,75.57) | 1468(1043,2002) | 28.86(22.88,37.15) | -3.55(-3.65,-3.45) | <0.001 |
| Hungary | 972(919,1026) | 68.78(47.85,85.59) | 211(175,250) | 36.59(28.07,46.83) | -3.88(-4.30,-3.45) | <0.001 |
| Iceland | 21(19,23) | 120.79(83.14,143.61) | 9(8,11) | 20.60(17.51,23.94) | -2.90(-3.11,-2.69) | <0.001 |
| India | 255212(190214,317767) | 105.82(74.71,129.46) | 130538(104553,166039) | 40.17(30.99,52.50) | -2.55(-2.78,-2.32) | <0.001 |
| Indonesia | 57840(40377,71892) | 33.80(31.76,35.85) | 31506(24263,40236) | 12.27(10.64,14.07) | -2.00(-2.08,-1.92) | <0.001 |
| Iran (Islamic Republic of) | 38847(26704,46212) | 34.20(32.15,36.11) | 5151(4397,5955) | 10.41(9.10,12.00) | -5.65(-6.04,-5.26) | <0.001 |
| Iraq | 11846(8320,14511) | 29.64(28.84,30.54) | 6625(5118,8636) | 10.38(8.99,11.78) | -3.29(-3.54,-3.04) | <0.001 |
| Ireland | 390(367,413) | 46.03(40.41,52.60) | 146(127,167) | 25.38(19.38,33.38) | -2.96(-3.41,-2.51) | <0.001 |
| Israel | 662(622,698) | 22.70(22.01,23.42) | 351(307,405) | 9.85(8.98,10.75) | -4.15(-4.45,-3.85) | <0.001 |
| Italy | 3487(3397,3589) | 85.91(68.01,101.06) | 989(872,1107) | 33.18(27.05,41.64) | -3.54(-3.77,-3.32) | <0.001 |
| Jamaica | 485(426,553) | 44.52(39.55,50.01) | 182(140,237) | 27.17(22.71,32.65) | -1.87(-2.19,-1.55) | <0.001 |
| Japan | 6459(6272,6651) | 50.94(36.36,74.71) | 1900(1748,2058) | 31.97(23.57,43.15) | -2.81(-3.02,-2.60) | <0.001 |
| Jordan | 1871(1479,2202) | 87.87(48.62,115.47) | 1444(1181,1803) | 56.56(37.03,73.82) | -3.07(-3.22,-2.92) | <0.001 |
| Kazakhstan | 3027(2687,3404) | 51.38(45.54,57.50) | 1889(1575,2275) | 27.48(22.63,33.21) | -1.44(-2.06,-0.82) | <0.001 |
| Kenya | 7679(5449,11342) | 44.07(39.31,49.28) | 7464(5532,10039) | 26.72(22.67,31.14) | -1.53(-1.70,-1.36) | <0.001 |
| Kiribati | 35(19,47) | 144.83(64.03,210.85) | 30(20,39) | 64.40(43.14,89.73) | -1.40(-1.56,-1.24) | <0.001 |
| Kuwait | 374(331,419) | 46.55(43.98,49.30) | 279(230,337) | 10.84(9.46,12.32) | -1.34(-2.79,0.12) | 0.07 |
| Kyrgyzstan | 991(882,1109) | 50.77(33.17,65.41) | 764(648,891) | 18.42(13.65,24.85) | -1.64(-2.10,-1.18) | <0.001 |
| Lao People's Democratic Republic | 3636(1560,5324) | 39.18(26.22,51.54) | 1957(1310,2729) | 42.27(29.07,57.61) | -2.62(-2.71,-2.53) | <0.001 |
| Latvia | 350(330,370) | 152.02(81.99,201.72) | 40(35,45) | 57.79(41.34,80.82) | -4.77(-5.23,-4.31) | <0.001 |
| Lebanon | 711(462,918) | 107.74(82.30,132.93) | 291(217,390) | 73.26(56.40,93.33) | -3.18(-3.41,-2.96) | <0.001 |
| Lesotho | 343(228,452) | 40.51(37.73,43.06) | 339(234,460) | 12.80(11.28,14.50) | 0.30(0.14,0.45) | <0.001 |
| Liberia | 2485(1317,3295) | 33.65(30.93,36.74) | 1621(1160,2265) | 9.38(7.88,11.13) | -3.59(-4.06,-3.13) | <0.001 |
| Libya | 2516(1921,3105) | 58.42(45.55,69.63) | 1274(986,1617) | 13.31(10.89,16.30) | -1.18(-1.73,-0.63) | <0.001 |
| Lithuania | 439(409,467) | 100.90(69.30,144.56) | 66(58,75) | 58.42(43.25,78.30) | -3.52(-3.94,-3.10) | <0.001 |
| Luxembourg | 29(27,32) | 130.48(74.56,191.60) | 12(10,14) | 55.07(41.20,74.84) | -3.77(-4.30,-3.24) | <0.001 |
| Madagascar | 7592(5155,10971) | 33.16(25.51,39.67) | 8728(6462,11703) | 17.76(14.47,21.13) | -1.76(-1.89,-1.64) | <0.001 |
| Malawi | 8650(4845,12818) | 71.90(38.45,93.90) | 5671(4242,7703) | 24.23(18.93,31.53) | -2.74(-2.99,-2.49) | <0.001 |
| Malaysia | 2832(2170,3393) | 147.76(89.98,194.80) | 1752(1437,2077) | 76.19(55.43,103.37) | -2.37(-3.20,-1.53) | <0.001 |
| Maldives | 103(53,136) | 30.92(27.24,34.13) | 30(23,38) | 22.02(17.94,26.44) | -3.49(-3.66,-3.33) | <0.001 |
| Mali | 8884(5342,11698) | 38.23(27.58,47.63) | 12105(8814,16380) | 35.62(26.00,47.94) | -2.24(-2.53,-1.96) | <0.001 |
| Malta | 33(29,37) | 65.53(39.41,85.93) | 18(15,22) | 34.44(26.46,44.28) | -1.18(-1.57,-0.78) | <0.001 |
| Marshall Islands | 10(7,13) | 37.90(34.08,41.35) | 8(6,11) | 31.07(26.20,35.07) | -0.26(-0.40,-0.12) | <0.001 |
| Mauritania | 835(490,1101) | 58.56(53.44,64.09) | 815(626,1047) | 36.99(29.93,45.84) | -2.08(-2.25,-1.91) | <0.001 |
| Mauritius | 154(139,168) | 58.68(52.03,65.89) | 85(72,95) | 27.62(21.71,35.54) | -0.78(-1.25,-0.30) | <0.001 |
| Mexico | 25428(23209,27830) | 24.89(18.94,32.25) | 14589(11917,17911) | 16.17(13.87,18.62) | -1.70(-1.92,-1.48) | <0.001 |
| Micronesia (Federated States of) | 31(21,40) | 80.23(55.43,102.07) | 12(9,15) | 29.15(23.17,36.09) | -2.06(-2.21,-1.90) | <0.001 |
| Monaco | 1(1,1) | 25.10(21.31,29.35) | 1(1,1) | 8.56(6.61,11.05) | -0.13(-0.80,0.54) | 0.07 |
| Mongolia | 973(667,1242) | 83.11(66.81,103.12) | 393(310,489) | 25.70(18.98,36.41) | -3.20(-3.47,-2.93) | <0.001 |
| Montenegro | 51(43,59) | 129.59(73.74,202.87) | 13(10,16) | 61.06(43.94,87.62) | -3.60(-4.50,-2.69) | <0.001 |
| Morocco | 10754(8649,13340) | 147.30(76.60,205.24) | 3197(2367,4513) | 71.55(50.63,94.83) | -3.81(-3.94,-3.69) | <0.001 |
| Mozambique | 11147(6275,17556) | 36.98(25.88,47.21) | 11263(8108,16191) | 28.54(20.28,39.90) | -2.36(-2.69,-2.03) | <0.001 |
| Myanmar | 27596(14408,38421) | 55.26(39.40,70.67) | 14202(10088,18813) | 49.22(35.56,66.14) | -2.38(-2.50,-2.27) | <0.001 |
| Namibia | 296(207,378) | 67.93(52.88,84.97) | 299(213,419) | 24.25(16.72,37.65) | -0.75(-0.90,-0.59) | <0.001 |
| Nauru | 3(2,4) | 31.38(29.90,32.95) | 3(2,3) | 12.44(11.13,13.77) | -0.42(-0.56,-0.27) | <0.001 |
| Nepal | 7726(6029,9632) | 39.68(37.01,42.38) | 2974(2061,4583) | 14.87(13.12,16.51) | -3.20(-3.35,-3.06) | <0.001 |
| Netherlands | 1129(1076,1185) | 78.04(56.85,96.33) | 426(383,470) | 27.44(20.55,36.56) | -2.76(-3.21,-2.31) | <0.001 |
| New Zealand | 422(394,450) | 123.83(65.83,165.46) | 182(161,202) | 54.95(38.10,72.78) | -3.21(-3.57,-2.86) | <0.001 |
| Nicaragua | 1870(1356,2314) | 99.39(55.34,131.26) | 679(511,901) | 72.44(51.07,92.92) | -3.35(-3.48,-3.21) | <0.001 |
| Niger | 7251(3767,9710) | 39.46(29.61,50.21) | 9653(6688,12757) | 114.85(101.87,128.46) | -2.42(-2.77,-2.06) | <0.001 |
| Nigeria | 55440(30289,73362) | 53.30(39.15,70.36) | 96939(68426,124162) | 23.81(17.15,33.06) | -1.04(-1.11,-0.96) | <0.001 |
| Niue | 0(0,0) | 18.54(14.15,24.06) | 1(0,1) | 13.11(10.59,16.04) | 6.78(4.60,8.96) | <0.001 |
| North Macedonia | 377(295,448) | 30.56(29.02,32.37) | 57(47,69) | 10.44(9.49,11.56) | -4.68(-5.41,-3.94) | <0.001 |
| Northern Mariana Islands | 3(2,4) | 72.12(55.84,89.95) | 2(2,2) | 23.61(20.04,28.48) | -1.49(-3.06,0.09) | 0.06 |
| Norway | 323(307,342) | 69.31(51.88,88.06) | 124(113,136) | 49.07(38.09,63.03) | -3.22(-3.77,-2.68) | <0.001 |
| Oman | 828(641,1032) | 45.50(29.62,61.79) | 358(304,432) | 31.99(24.37,40.37) | -3.56(-3.86,-3.26) | <0.001 |
| Pakistan | 45009(33781,57215) | 86.27(64.41,103.26) | 53521(41533,68744) | 30.23(23.84,39.07) | -1.18(-1.43,-0.92) | <0.001 |
| Palau | 3(2,4) | 55.86(48.81,63.24) | 1(1,2) | 38.10(30.50,47.55) | -1.11(-1.20,-1.02) | <0.001 |
| Palestine | 1178(876,1409) | 85.09(41.01,116.37) | 702(555,904) | 72.28(38.69,99.72) | -3.12(-3.35,-2.90) | <0.001 |
| Panama | 594(519,672) | 41.10(33.48,51.98) | 541(434,673) | 30.96(21.69,43.55) | -1.27(-1.56,-0.98) | <0.001 |
| Papua New Guinea | 1959(941,2673) | 120.69(98.87,142.53) | 3862(2032,5339) | 30.42(21.51,40.11) | -0.38(-0.61,-0.16) | <0.001 |
| Paraguay | 905(735,1148) | 63.74(47.93,81.11) | 780(551,1091) | 36.23(29.93,43.59) | -0.80(-1.16,-0.43) | <0.001 |
| Peru | 12966(10620,15314) | 43.88(42.29,45.55) | 3760(2665,4952) | 12.99(10.95,15.25) | -4.34(-4.58,-4.09) | <0.001 |
| Philippines | 21172(15854,27023) | 40.56(38.44,42.93) | 15661(12966,18789) | 10.89(9.58,12.24) | -1.89(-2.14,-1.64) | <0.001 |
| Poland | 4936(4760,5120) | 33.49(31.35,35.58) | 949(804,1108) | 16.46(13.78,19.65) | -4.07(-4.58,-3.56) | <0.001 |
| Portugal | 1009(957,1066) | 50.44(38.04,62.45) | 196(173,218) | 15.27(12.03,19.39) | -3.99(-4.42,-3.56) | <0.001 |
| Puerto Rico | 418(392,444) | 52.98(49.34,56.63) | 84(71,100) | 15.84(13.76,17.97) | -2.31(-2.83,-1.79) | <0.001 |
| Qatar | 88(66,109) | 40.30(39.42,41.14) | 95(75,121) | 15.60(14.20,16.74) | -3.79(-4.08,-3.49) | <0.001 |
| Republic of Korea | 5143(4114,6056) | 117.69(75.11,170.43) | 587(498,677) | 48.67(36.81,64.41) | -4.60(-4.90,-4.29) | <0.001 |
| Republic of Moldova | 930(824,1044) | 45.98(41.35,51.18) | 171(135,217) | 33.48(26.68,41.63) | -2.21(-2.67,-1.75) | <0.001 |
| Romania | 3624(3378,3871) | 42.91(36.28,50.40) | 596(519,675) | 31.63(24.02,41.15) | -3.50(-3.90,-3.10) | <0.001 |
| Russian Federation | 17686(17301,18052) | 53.23(44.49,63.09) | 4852(4431,5195) | 34.19(27.69,42.20) | -2.84(-3.31,-2.36) | <0.001 |
| Rwanda | 5438(3394,7977) | 42.66(29.72,56.68) | 3138(2373,4153) | 26.54(19.40,35.43) | -2.75(-3.27,-2.22) | <0.001 |
| Saint Kitts and Nevis | 8(7,9) | 21.97(17.73,27.16) | 4(4,5) | 7.21(5.26,9.79) | -0.98(-1.27,-0.70) | <0.001 |
| Saint Lucia | 28(24,33) | 96.11(63.61,125.05) | 12(9,16) | 31.43(21.89,46.42) | -0.97(-1.20,-0.74) | <0.001 |
| Saint Vincent and the Grenadines | 27(22,32) | 90.97(61.88,118.73) | 11(9,13) | 15.87(12.42,20.52) | -1.36(-1.67,-1.04) | <0.001 |
| Samoa | 40(28,53) | 95.25(59.49,124.12) | 27(20,37) | 47.13(36.83,60.44) | -1.31(-1.43,-1.19) | <0.001 |
| San Marino | 1(1,1) | 54.73(42.33,62.97) | 0(0,1) | 10.83(9.07,13.06) | -3.50(-3.64,-3.35) | <0.001 |
| Sao Tome and Principe | 71(47,92) | 32.76(28.21,38.06) | 31(22,45) | 22.89(17.89,28.72) | -3.25(-4.37,-2.13) | <0.001 |
| Saudi Arabia | 7938(5386,10361) | 155.42(77.25,203.13) | 1559(1226,2002) | 77.99(56.45,101.19) | -5.48(-5.59,-5.36) | <0.001 |
| Senegal | 4835(2974,6310) | 36.54(33.75,39.49) | 3884(3036,4979) | 8.71(7.60,9.93) | -2.31(-2.94,-1.68) | <0.001 |
| Serbia | 1441(1123,1654) | 30.41(27.13,33.41) | 177(149,211) | 15.39(12.75,18.31) | -4.27(-5.71,-2.83) | <0.001 |
| Seychelles | 10(9,12) | 26.84(24.74,29.01) | 7(5,8) | 7.50(6.48,8.56) | -0.95(-2.25,0.35) | 0.15 |
| Sierra Leone | 4233(2060,5532) | 49.57(30.16,67.50) | 3757(2717,4874) | 33.62(25.14,44.01) | -2.25(-2.62,-1.87) | <0.001 |
| Singapore | 297(275,320) | 95.97(55.32,150.67) | 90(79,103) | 60.99(39.37,93.35) | -4.25(-4.98,-3.53) | <0.001 |
| Slovakia | 488(436,536) | 39.27(33.68,45.93) | 167(139,198) | 28.54(21.88,35.85) | -2.23(-2.89,-1.56) | <0.001 |
| Slovenia | 134(123,144) | 35.55(28.06,42.29) | 29(25,33) | 7.90(6.62,9.21) | -4.03(-4.36,-3.70) | <0.001 |
| Solomon Islands | 104(63,141) | 110.97(60.17,170.41) | 114(85,150) | 100.81(64.34,151.92) | -1.31(-1.64,-0.98) | <0.001 |
| Somalia | 5163(2935,8184) | 31.07(29.70,32.44) | 8612(5531,13311) | 10.86(9.74,11.93) | -1.57(-1.72,-1.42) | <0.001 |
| South Africa | 7008(6010,8199) | 39.95(31.91,49.25) | 5430(4187,6797) | 16.94(12.62,22.19) | -0.92(-1.11,-0.74) | <0.001 |
| South Sudan | 4053(2177,6248) | 239.64(127.53,308.03) | 5720(3643,8635) | 85.73(64.61,111.59) | -0.37(-0.55,-0.19) | <0.001 |
| Spain | 2865(2737,2991) | 66.09(50.76,78.27) | 870(787,949) | 43.76(32.69,56.57) | -3.66(-4.09,-3.23) | <0.001 |
| Sri Lanka | 2784(2235,3414) | 42.70(31.07,52.68) | 1108(828,1448) | 37.19(26.09,50.02) | -2.68(-3.20,-2.16) | <0.001 |
| Sudan | 30012(15850,38569) | 29.12(27.57,30.66) | 17952(13524,23371) | 9.73(8.67,10.93) | -3.31(-3.37,-3.25) | <0.001 |
| Suriname | 109(83,129) | 35.02(33.09,37.01) | 77(58,99) | 13.01(11.26,14.86) | -1.25(-1.67,-0.82) | <0.001 |
| Sweden | 611(578,643) | 110.37(82.69,135.02) | 225(201,253) | 35.72(27.64,44.90) | -3.46(-3.97,-2.95) | <0.001 |
| Switzerland | 533(503,563) | 25.47(24.23,26.75) | 220(191,251) | 12.07(10.57,13.46) | -3.08(-3.38,-2.77) | <0.001 |
| Syrian Arab Republic | 8507(6350,10414) | 51.65(44.11,62.68) | 1749(1365,2191) | 36.49(26.41,54.10) | -3.56(-3.97,-3.15) | <0.001 |
| Taiwan (Province of China) | 1694(1613,1778) | 111.31(67.21,161.72) | 457(403,507) | 65.57(48.73,89.01) | -2.36(-2.88,-1.84) | <0.001 |
| Tajikistan | 1667(1419,2037) | 44.98(36.28,54.88) | 1716(1233,2570) | 19.97(16.04,24.17) | -1.02(-1.31,-0.73) | <0.001 |
| Thailand | 9444(7652,11476) | 43.73(36.94,51.32) | 2553(2054,3093) | 28.16(21.76,36.90) | -2.59(-2.88,-2.31) | <0.001 |
| Timor-Leste | 517(230,740) | 73.72(47.76,107.28) | 352(252,472) | 46.65(34.23,68.39) | -2.36(-2.64,-2.08) | <0.001 |
| Togo | 2296(1469,2971) | 105.82(48.79,150.66) | 2298(1674,3171) | 51.36(36.82,68.89) | -1.93(-2.17,-1.69) | <0.001 |
| Tokelau | 0(0,0) | 96.33(62.23,124.81) | 1(0,1) | 54.39(39.54,75.20) | 12.23(6.68,17.78) | <0.001 |
| Tonga | 16(12,20) | 42.01(29.26,53.69) | 11(8,14) | 127.73(108.90,155.45) | -0.94(-1.25,-0.62) | <0.001 |
| Trinidad and Tobago | 264(231,299) | 28.82(22.18,36.41) | 122(94,158) | 21.26(16.08,28.49) | -1.17(-1.57,-0.76) | <0.001 |
| Tunisia | 4070(2641,5061) | 52.36(45.73,59.44) | 943(735,1175) | 37.29(28.49,48.43) | -4.16(-4.30,-4.03) | <0.001 |
| Turkey | 39565(24622,48833) | 103.19(66.89,128.38) | 7586(6196,9250) | 27.72(21.60,34.52) | -4.72(-4.91,-4.53) | <0.001 |
| Turkmenistan | 1078(948,1221) | 153.13(94.98,189.10) | 1103(891,1364) | 34.19(27.84,41.82) | 0.51(0.08,0.94) | 0.02 |
| Tuvalu | 4(2,6) | 52.74(46.50,59.60) | 2(1,2) | 55.88(45.13,69.06) | -3.06(-3.21,-2.91) | <0.001 |
| Uganda | 10265(6589,15509) | 84.36(46.72,114.34) | 13964(10691,18688) | 31.68(24.05,41.50) | -1.64(-2.01,-1.27) | <0.001 |
| Ukraine | 6442(5725,7240) | 82.33(53.23,123.53) | 1412(1167,1674) | 53.64(41.07,71.68) | -2.47(-2.75,-2.19) | <0.001 |
| United Arab Emirates | 484(374,604) | 44.76(39.70,50.39) | 291(234,348) | 19.72(16.30,23.39) | -4.47(-5.01,-3.93) | <0.001 |
| United Kingdom | 4803(4670,4941) | 62.40(48.39,77.95) | 1910(1717,2073) | 17.85(14.38,21.37) | -3.03(-3.46,-2.61) | <0.001 |
| United Republic of Tanzania | 18877(11234,27598) | 33.06(32.13,34.03) | 20908(15545,28388) | 13.01(11.62,14.19) | -1.70(-1.84,-1.55) | <0.001 |
| United States of America | 24185(23691,24697) | 32.97(32.29,33.67) | 12843(11767,13937) | 16.98(15.51,18.49) | -2.04(-2.19,-1.89) | <0.001 |
| United States Virgin Islands | 15(12,18) | 46.14(43.41,48.86) | 3(2,4) | 23.15(19.17,28.04) | -3.05(-3.53,-2.56) | <0.001 |
| Uruguay | 475(447,503) | 37.48(33.75,42.02) | 188(157,225) | 35.15(28.86,43.38) | -2.30(-3.02,-1.58) | <0.001 |
| Uzbekistan | 4311(3871,4852) | 45.39(27.89,61.12) | 4725(3868,5850) | 34.02(24.94,44.27) | -0.16(-0.39,0.08) | 0.18 |
| Vanuatu | 41(25,55) | 42.72(40.71,44.89) | 51(37,66) | 39.11(28.66,50.87) | -0.54(-1.11,0.03) | 0.06 |
| Venezuela (Bolivarian Republic of) | 3936(3750,4136) | 31.39(21.04,39.83) | 3291(2419,4274) | 15.62(11.41,20.48) | -0.11(-0.80,0.59) | 0.77 |
| Viet Nam | 10738(7186,13622) | 36.18(29.35,43.04) | 4787(3501,6271) | 15.44(10.71,21.07) | -2.19(-2.28,-2.09) | <0.001 |
| Yemen | 18375(9276,24055) | 183.29(92.73,240.89) | 14104(10281,18334) | 81.28(59.18,105.77) | -2.59(-2.76,-2.42) | <0.001 |
| Zambia | 5677(3316,8775) | 106.70(63.40,163.39) | 5920(4205,8265) | 56.08(39.79,78.26) | -2.15(-2.46,-1.85) | <0.001 |
| Zimbabwe | 1990(1503,2474) | 31.79(24.02,39.57) | 3527(2608,4592) | 44.07(32.61,57.38) | 1.00(0.58,1.43) | <0.001 |

Supplementary Table 6：Numbers and ASDR of non-communicable diseases in children and adolescents in 204 countries in 1990 and 2021, along with the Average Annual Percentage Change (AAPC) in ASDR from 1990 to 2021.

| **Location** | **1990-Number** | **1990- ASDR** | **2021-Number** | **2021- ASDR** | **AAPC(95%CI)** | ***P*** |
| --- | --- | --- | --- | --- | --- | --- |
| China | 41141277(32340568,50829766) | 9453.54(7423.34,11681.54) | 13214853(10422790,16697782) | 4009.25(3173.32,5055.72) | -2.81(-2.91,-2.71) | <0.001 |
| Democratic People's Republic of Korea | 606848(468415,771616) | 7452.45(5753.46,9470.48) | 317412(243404,415420) | 4763.36(3640.47,6261.16) | -1.43(-1.49,-1.37) | <0.001 |
| Taiwan (Province of China) | 360667(297675,441515) | 5012.22(4184.73,6070.30) | 164345(127430,212517) | 3987.13(3111.16,5124.80) | -0.70(-0.86,-0.53) | <0.001 |
| Cambodia | 870537(494848,1149951) | 14036.82(8237.95,18447.12) | 517251(401052,660091) | 7861.67(6096.83,10032.56) | -1.86(-1.94,-1.78) | <0.001 |
| Indonesia | 7567481(5696375,9391437) | 8866.98(6652.92,11002.27) | 5407608(4244116,6813061) | 6036.83(4740.60,7601.02) | -1.27(-1.32,-1.21) | <0.001 |
| Lao People's Democratic Republic | 385678(192841,547126) | 15722.08(8152.42,22125.78) | 261896(189668,348206) | 8653.99(6273.58,11499.79) | -1.97(-2.02,-1.91) | <0.001 |
| Malaysia | 483507(377365,596091) | 5772.36(4507.41,7124.52) | 481679(373990,614500) | 4618.98(3599.00,5871.99) | -0.76(-1.02,-0.50) | <0.001 |
| Maldives | 13214(8140,17110) | 9664.91(6156.29,12466.78) | 6837(5280,8825) | 5324.66(4119.86,6858.55) | -1.89(-1.96,-1.82) | <0.001 |
| Myanmar | 2973095(1752888,3995778) | 15801.68(9272.43,21262.30) | 1892579(1404752,2443653) | 9317.88(6893.19,12041.09) | -1.78(-1.85,-1.70) | <0.001 |
| Philippines | 2837508(2176519,3622812) | 8682.13(6671.83,11076.62) | 2788436(2235697,3474523) | 6269.15(5034.42,7798.92) | -1.08(-1.23,-0.92) | <0.001 |
| Sri Lanka | 455783(360779,571667) | 6364.95(5036.14,7985.20) | 317427(244917,411545) | 4504.75(3483.88,5830.09) | -1.85(-2.22,-1.47) | <0.001 |
| Thailand | 1542001(1237677,1937026) | 6949.28(5593.07,8704.44) | 666360(522994,852276) | 4754.37(3755.78,6040.25) | -1.19(-1.30,-1.07) | <0.001 |
| Timor-Leste | 57133(30366,79269) | 12313.90(6868.91,16914.45) | 51798(39335,67801) | 7517.60(5707.81,9841.42) | -1.64(-1.75,-1.53) | <0.001 |
| Viet Nam | 1851850(1344892,2352029) | 5491.26(3992.33,6980.86) | 1293859(981103,1692946) | 4136.00(3138.07,5407.78) | -0.86(-0.94,-0.78) | <0.001 |
| Fiji | 23853(19059,29553) | 6725.41(5372.31,8333.40) | 23576(18403,29827) | 6783.27(5289.70,8585.33) | 0.06(0.01,0.10) | 0.01 |
| Kiribati | 4094(2529,5312) | 10537.39(6735.81,13595.34) | 4122(2961,5208) | 7788.18(5591.67,9837.43) | -1.00(-1.15,-0.85) | <0.001 |
| Marshall Islands | 1601(1214,1983) | 6125.24(4657.74,7584.73) | 1377(1055,1755) | 5952.08(4550.92,7596.90) | -0.08(-0.15,-0.00) | 0.04 |
| Micronesia (Federated States of) | 4326(3120,5467) | 7607.46(5496.51,9606.71) | 2281(1785,2861) | 5413.42(4235.56,6799.06) | -1.14(-1.23,-1.06) | <0.001 |
| Papua New Guinea | 232996(137656,304495) | 10408.10(6262.02,13583.79) | 478643(297546,637590) | 9281.66(5893.34,12319.38) | -0.30(-0.45,-0.15) | <0.001 |
| Samoa | 6013(4467,7738) | 6517.72(4848.15,8381.74) | 5230(3993,6737) | 5202.43(3977.79,6694.47) | -0.67(-0.77,-0.56) | <0.001 |
| Solomon Islands | 14382(10198,18776) | 7143.84(5105.96,9310.68) | 19343(15118,24629) | 5813.90(4544.83,7398.67) | -0.73(-0.93,-0.53) | <0.001 |
| Tonga | 2894(2258,3708) | 5380.58(4196.84,6895.09) | 2371(1854,3065) | 4794.60(3746.82,6199.33) | -0.32(-0.45,-0.19) | <0.001 |
| Vanuatu | 5727(4041,7370) | 6702.18(4813.42,8588.54) | 8501(6568,10824) | 5796.57(4484.63,7372.87) | -0.28(-0.55,-0.01) | 0.04 |
| Armenia | 96368(80594,115534) | 7116.39(5932.11,8557.77) | 38310(29799,48584) | 5105.24(3985.09,6458.39) | -0.90(-1.08,-0.71) | <0.001 |
| Azerbaijan | 267440(220013,323759) | 8350.27(6862.53,10123.83) | 186385(146659,238628) | 6284.53(4947.06,8059.09) | -0.93(-1.02,-0.85) | <0.001 |
| Georgia | 98520(80128,121421) | 5483.90(4467.91,6749.18) | 43844(34277,56029) | 4801.00(3759.13,6126.25) | -0.51(-0.76,-0.25) | <0.001 |
| Kazakhstan | 460781(385665,552087) | 6864.10(5735.26,8236.83) | 365863(290678,460484) | 5448.60(4319.97,6869.75) | -0.64(-0.95,-0.34) | <0.001 |
| Kyrgyzstan | 149299(124756,177771) | 6890.76(5730.88,8241.53) | 153037(121628,189561) | 5474.02(4342.59,6790.52) | -0.70(-0.90,-0.50) | <0.001 |
| Mongolia | 119856(86029,151289) | 10122.30(7310.23,12762.04) | 72404(56764,91059) | 5601.04(4389.80,7050.90) | -1.89(-2.01,-1.78) | <0.001 |
| Tajikistan | 229765(190425,284482) | 7548.21(6217.10,9367.89) | 283864(212852,391138) | 6285.61(4724.27,8604.85) | -0.55(-0.70,-0.40) | <0.001 |
| Turkmenistan | 151423(127642,179882) | 7708.64(6466.53,9198.46) | 156824(127125,192759) | 7972.01(6461.23,9800.09) | 0.21(-0.11,0.54) | 0.20 |
| Uzbekistan | 689477(577734,831808) | 6299.08(5246.75,7643.60) | 794436(640466,993096) | 6188.69(4973.28,7755.14) | -0.11(-0.23,0.01) | 0.07 |
| Albania | 111899(91557,133833) | 7657.94(6262.97,9164.31) | 33872(26155,43268) | 5507.27(4265.95,7025.85) | -1.13(-1.21,-1.06) | <0.001 |
| Bosnia and Herzegovina | 76416(60814,94412) | 5165.51(4134.66,6352.21) | 29211(22381,37471) | 4281.66(3297.11,5472.74) | -0.58(-0.76,-0.40) | <0.001 |
| Bulgaria | 162068(137437,190662) | 7091.86(6083.83,8254.93) | 64174(50618,80756) | 4917.65(3900.55,6164.26) | -1.23(-1.46,-1.00) | <0.001 |
| Croatia | 71165(58573,86538) | 5467.06(4551.29,6581.63) | 34971(26728,44913) | 4252.01(3271.61,5437.32) | -0.86(-1.06,-0.65) | <0.001 |
| Czechia | 167710(136933,203288) | 5588.88(4642.41,6682.94) | 86607(65697,112563) | 3855.42(2931.63,5003.16) | -1.23(-1.32,-1.14) | <0.001 |
| Hungary | 182314(152830,218248) | 6525.04(5563.18,7687.43) | 81250(62351,103754) | 4223.19(3252.02,5382.50) | -1.46(-1.54,-1.37) | <0.001 |
| North Macedonia | 54462(42356,66468) | 8123.12(6318.06,9886.04) | 19901(15524,25281) | 4330.78(3390.92,5488.86) | -1.93(-2.14,-1.72) | <0.001 |
| Montenegro | 10975(8735,13600) | 5149.20(4117.20,6359.28) | 6104(4601,7975) | 3908.04(2947.98,5106.68) | -0.78(-1.06,-0.50) | <0.001 |
| Poland | 811228(694811,948981) | 6839.30(5915.43,7927.03) | 355389(272138,457189) | 4593.19(3532.11,5895.43) | -1.26(-1.43,-1.09) | <0.001 |
| Romania | 549634(470999,640631) | 7588.79(6568.97,8765.05) | 185642(146213,234065) | 4524.73(3593.57,5669.33) | -1.50(-1.91,-1.09) | <0.001 |
| Serbia | 216366(168123,261201) | 7788.43(6049.62,9351.69) | 79049(59788,102164) | 4018.52(3071.84,5155.02) | -2.07(-2.58,-1.56) | <0.001 |
| Slovakia | 96344(78274,116655) | 5608.07(4594.28,6734.97) | 50906(39416,64700) | 4509.02(3501.37,5718.25) | -0.72(-0.89,-0.56) | <0.001 |
| Slovenia | 29356(23999,36026) | 5369.26(4445.58,6520.41) | 15530(11712,20162) | 3798.93(2874.39,4920.41) | -1.09(-1.19,-0.99) | <0.001 |
| Belarus | 204745(167904,250327) | 6620.02(5438.51,8079.39) | 85497(65089,110509) | 4246.62(3242.93,5477.70) | -1.31(-1.57,-1.04) | <0.001 |
| Estonia | 29201(24774,34624) | 6358.71(5406.21,7526.48) | 11997(9264,15373) | 4221.50(3264.91,5403.02) | -1.28(-1.44,-1.13) | <0.001 |
| Latvia | 51332(44472,59429) | 6790.54(5889.78,7854.54) | 15563(11925,19976) | 3991.92(3071.62,5107.74) | -1.79(-1.98,-1.60) | <0.001 |
| Lithuania | 71122(60444,83548) | 6424.83(5480.09,7525.21) | 23489(18145,29834) | 4343.14(3367.61,5502.22) | -1.33(-1.71,-0.95) | <0.001 |
| Republic of Moldova | 126489(106911,149268) | 8004.15(6762.44,9449.29) | 36978(28472,47384) | 5446.14(4206.67,6980.46) | -1.25(-1.42,-1.07) | <0.001 |
| Russian Federation | 2920267(2510747,3414520) | 6539.16(5641.17,7622.78) | 1514479(1187840,1909081) | 4526.65(3577.25,5674.58) | -1.13(-1.36,-0.91) | <0.001 |
| Ukraine | 1009229(838558,1208710) | 6809.56(5677.21,8129.04) | 398228(308723,501609) | 4783.67(3754.02,5963.81) | -1.07(-1.25,-0.88) | <0.001 |
| Brunei Darussalam | 8259(6843,9851) | 7065.35(5834.69,8452.13) | 7754(6062,9756) | 6066.75(4754.61,7619.11) | -0.51(-0.64,-0.38) | <0.001 |
| Japan | 1861305(1493851,2314786) | 5565.70(4547.56,6824.70) | 973755(746675,1250083) | 4441.79(3432.41,5675.29) | -0.71(-0.79,-0.63) | <0.001 |
| Republic of Korea | 1010127(805778,1241437) | 6408.80(5120.69,7844.08) | 364007(273552,471007) | 4145.22(3139.63,5341.60) | -1.32(-1.40,-1.24) | <0.001 |
| Singapore | 58185(48391,69580) | 6471.80(5452.73,7660.00) | 41664(32335,53991) | 4037.26(3132.58,5232.51) | -1.45(-1.61,-1.30) | <0.001 |
| Australia | 355776(294732,430705) | 6817.41(5696.62,8195.33) | 311773(240029,399413) | 4910.69(3793.93,6277.11) | -0.94(-1.09,-0.80) | <0.001 |
| New Zealand | 85533(70834,102864) | 7656.85(6379.50,9173.84) | 70352(54345,89741) | 5313.06(4126.86,6752.03) | -1.16(-1.26,-1.07) | <0.001 |
| Andorra | 815(615,1045) | 5969.58(4532.53,7616.83) | 733(537,980) | 4581.22(3377.81,6098.22) | -0.70(-0.90,-0.50) | <0.001 |
| Austria | 125260(103229,151434) | 6680.98(5572.12,8002.19) | 88508(66313,114822) | 4879.09(3671.91,6316.77) | -1.04(-1.18,-0.89) | <0.001 |
| Belgium | 165489(134957,202357) | 6676.71(5505.41,8090.22) | 132975(99534,172674) | 5093.04(3836.65,6584.55) | -0.90(-1.07,-0.73) | <0.001 |
| Cyprus | 16095(12150,20242) | 6287.04(4749.91,7880.77) | 13092(9663,17516) | 4542.32(3356.98,6072.03) | -0.97(-1.05,-0.90) | <0.001 |
| Denmark | 84623(68789,103735) | 6700.22(5529.55,8112.06) | 65673(49921,84565) | 4870.40(3719.99,6249.90) | -1.06(-1.15,-0.97) | <0.001 |
| Finland | 75145(60488,93137) | 5926.09(4801.80,7303.86) | 58092(43970,75294) | 4811.54(3668.46,6209.58) | -0.65(-0.79,-0.51) | <0.001 |
| France | 1084085(883549,1330910) | 6666.75(5494.89,8112.54) | 854273(636470,1115224) | 5222.90(3922.08,6785.38) | -0.75(-0.87,-0.64) | <0.001 |
| Germany | 1168141(957840,1417444) | 6683.37(5508.27,8077.72) | 855050(646532,1111362) | 5245.26(3977.56,6803.29) | -0.78(-0.87,-0.69) | <0.001 |
| Greece | 180866(145385,224440) | 6543.53(5377.58,7968.92) | 106345(79896,136871) | 5307.00(4023.92,6795.74) | -0.71(-0.80,-0.62) | <0.001 |
| Iceland | 5242(4190,6538) | 6163.39(4952.08,7657.94) | 4433(3373,5763) | 4833.51(3686.37,6274.29) | -0.66(-0.76,-0.55) | <0.001 |
| Ireland | 91101(73864,111739) | 6977.32(5744.59,8447.61) | 73130(55154,94791) | 5400.20(4104.33,6963.77) | -0.82(-0.99,-0.64) | <0.001 |
| Israel | 129875(107262,157592) | 6511.65(5402.59,7869.44) | 152485(115575,197615) | 4536.67(3437.23,5880.48) | -1.18(-1.33,-1.04) | <0.001 |
| Italy | 896102(720430,1114351) | 6411.01(5282.52,7821.44) | 552561(412640,722013) | 4976.51(3749.01,6463.57) | -0.88(-1.01,-0.75) | <0.001 |
| Luxembourg | 6054(4950,7405) | 6801.89(5581.26,8297.31) | 6698(5030,8803) | 4822.43(3633.50,6323.83) | -1.14(-1.34,-0.94) | <0.001 |
| Malta | 7333(5887,9093) | 6410.98(5183.73,7902.29) | 4954(3819,6290) | 5858.38(4522.73,7429.50) | -0.23(-0.37,-0.08) | <0.001 |
| Netherlands | 247402(203064,302790) | 6349.85(5269.86,7701.42) | 195198(147458,252716) | 5066.03(3858.74,6523.04) | -0.60(-0.75,-0.45) | <0.001 |
| Norway | 72834(59302,88861) | 6425.11(5279.60,7784.72) | 62504(47255,81533) | 4834.39(3675.19,6283.05) | -0.85(-0.98,-0.71) | <0.001 |
| Portugal | 223683(180760,274127) | 7613.65(6315.38,9147.91) | 111711(83335,146850) | 5415.31(4078.04,7088.74) | -1.04(-1.23,-0.85) | <0.001 |
| Spain | 717643(578731,891632) | 6437.01(5329.61,7821.22) | 467762(355199,609764) | 5046.91(3868.99,6537.65) | -0.76(-0.86,-0.65) | <0.001 |
| Sweden | 134837(110642,164614) | 6255.28(5154.70,7616.53) | 117487(88809,152515) | 4741.05(3600.54,6136.00) | -0.78(-0.91,-0.65) | <0.001 |
| Switzerland | 112781(92659,136388) | 7047.77(5830.62,8477.53) | 92355(70051,118692) | 5151.50(3921.68,6604.58) | -0.97(-1.12,-0.83) | <0.001 |
| United Kingdom | 1037837(855412,1257259) | 6887.51(5708.12,8310.78) | 838978(641225,1076419) | 5229.29(4020.18,6680.51) | -0.81(-0.94,-0.68) | <0.001 |
| Argentina | 1024209(885164,1191116) | 7943.31(6877.16,9222.85) | 814626(633186,1028068) | 6008.32(4705.59,7544.46) | -0.95(-1.06,-0.84) | <0.001 |
| Chile | 382543(324575,448532) | 7169.52(6090.25,8400.53) | 268905(207848,339354) | 5436.50(4235.02,6819.61) | -0.91(-1.03,-0.78) | <0.001 |
| Uruguay | 79508(67459,93237) | 7483.07(6380.24,8736.86) | 52122(40583,65759) | 5739.93(4497.34,7206.17) | -1.01(-1.18,-0.84) | <0.001 |
| Canada | 470035(387831,571068) | 6123.94(5088.80,7398.90) | 408975(311650,523890) | 4843.73(3714.11,6183.58) | -0.68(-0.77,-0.59) | <0.001 |
| United States of America | 5026269(4165566,6055282) | 6730.63(5594.38,8095.03) | 5057594(3912834,6366063) | 5967.73(4659.56,7472.39) | -0.33(-0.43,-0.22) | <0.001 |
| Antigua and Barbuda | 1417(1128,1782) | 5946.29(4745.55,7463.62) | 1395(1099,1761) | 5892.15(4682.72,7384.65) | -0.07(-0.18,0.04) | 0.21 |
| Bahamas | 7590(6083,9451) | 7230.20(5814.07,8960.45) | 7018(5389,9044) | 6062.77(4678.79,7800.20) | -0.64(-0.89,-0.39) | <0.001 |
| Barbados | 5868(4764,7249) | 6998.04(5703.55,8599.67) | 4170(3188,5416) | 6294.51(4811.44,8196.43) | -0.37(-0.94,0.19) | 0.20 |
| Belize | 9466(8005,11375) | 9052.68(7628.21,10917.86) | 10101(7930,12936) | 5980.69(4718.10,7630.47) | -1.30(-1.44,-1.15) | <0.001 |
| Cuba | 245089(202846,297648) | 6550.01(5459.00,7899.34) | 121468(92976,156802) | 4969.73(3822.05,6390.85) | -0.87(-0.95,-0.78) | <0.001 |
| Dominica | 2162(1720,2710) | 6559.68(5220.78,8212.93) | 1550(1203,1978) | 8587.98(6642.16,11032.17) | 0.84(0.70,0.98) | <0.001 |
| Dominican Republic | 382951(320249,459384) | 10678.18(8922.82,12820.15) | 258434(197954,353154) | 6604.79(5057.18,9038.86) | -1.46(-1.57,-1.35) | <0.001 |
| Grenada | 3153(2548,3898) | 7443.60(6009.24,9213.50) | 1942(1537,2464) | 6412.41(5090.35,8100.63) | -0.43(-0.54,-0.33) | <0.001 |
| Guyana | 34168(28471,41139) | 8673.93(7218.93,10461.59) | 20210(15604,25971) | 7171.09(5534.57,9218.23) | -0.60(-0.88,-0.31) | <0.001 |
| Haiti | 773505(612283,935183) | 21091.76(16718.52,25515.97) | 808669(629470,1048484) | 14209.43(11065.61,18415.22) | -1.22(-1.35,-1.09) | <0.001 |
| Jamaica | 81182(66674,99857) | 7527.73(6198.00,9234.93) | 47348(36061,61805) | 5808.99(4440.32,7565.50) | -0.85(-1.00,-0.69) | <0.001 |
| Saint Lucia | 4796(3869,5946) | 7215.15(5825.54,8935.50) | 2680(2078,3467) | 6341.04(4905.97,8216.18) | -0.41(-0.55,-0.27) | <0.001 |
| Saint Vincent and the Grenadines | 4167(3379,5170) | 7997.24(6499.92,9898.85) | 2188(1737,2765) | 6471.08(5139.42,8174.69) | -0.68(-0.87,-0.49) | <0.001 |
| Suriname | 15484(12448,18894) | 9214.78(7421.43,11226.50) | 14009(10827,17956) | 7518.96(5798.51,9643.33) | -0.62(-0.81,-0.42) | <0.001 |
| Trinidad and Tobago | 40213(33280,48479) | 7888.53(6534.77,9500.16) | 24230(18756,31380) | 6809.55(5275.38,8816.93) | -0.56(-0.74,-0.37) | <0.001 |
| Bolivia (Plurinational State of) | 652317(439938,818727) | 18294.42(12437.97,22930.45) | 409520(328006,506194) | 9088.63(7282.97,11229.18) | -2.24(-2.32,-2.17) | <0.001 |
| Ecuador | 482462(416819,564633) | 9742.35(8416.93,11401.23) | 428596(331568,549993) | 6531.85(5056.17,8377.90) | -1.30(-1.54,-1.07) | <0.001 |
| Peru | 1521360(1236967,1817066) | 14196.91(11543.31,16958.58) | 771502(585229,991958) | 6153.73(4665.21,7915.99) | -2.63(-2.86,-2.39) | <0.001 |
| Colombia | 1140762(946162,1365614) | 7508.82(6222.41,8999.95) | 849167(643822,1111174) | 5779.76(4379.64,7577.44) | -1.05(-1.17,-0.92) | <0.001 |
| Costa Rica | 97260(82166,116562) | 6799.33(5719.98,8183.08) | 78185(61256,98843) | 5736.18(4523.64,7212.58) | -0.52(-0.72,-0.32) | <0.001 |
| El Salvador | 312482(248000,378425) | 11197.28(8887.73,13568.60) | 136172(103423,177611) | 5720.33(4341.06,7467.61) | -2.11(-2.21,-2.01) | <0.001 |
| Guatemala | 506130(438026,583673) | 9902.46(8511.22,11508.34) | 455023(356338,583048) | 7013.22(5503.67,8981.63) | -1.06(-1.29,-0.83) | <0.001 |
| Honduras | 360458(303184,428081) | 12650.01(10610.26,15074.53) | 278218(212222,364800) | 6420.69(4899.11,8424.79) | -2.20(-2.27,-2.13) | <0.001 |
| Mexico | 3619515(3094473,4272952) | 8319.82(7115.66,9818.16) | 2740989(2142645,3475413) | 6473.10(5068.59,8192.75) | -0.87(-0.94,-0.80) | <0.001 |
| Nicaragua | 239954(177980,300996) | 10313.56(7664.21,12954.47) | 149156(112864,197584) | 5782.75(4377.72,7657.99) | -1.83(-1.88,-1.78) | <0.001 |
| Panama | 89550(73968,107781) | 8316.95(6880.30,9994.78) | 100138(78568,126566) | 6776.12(5324.78,8550.38) | -0.68(-0.83,-0.52) | <0.001 |
| Venezuela (Bolivarian Republic of) | 654646(554936,777614) | 7170.53(6068.55,8530.26) | 583372(446239,743965) | 6712.57(5129.33,8562.20) | -0.14(-0.39,0.12) | 0.29 |
| Brazil | 5286476(4228855,6604054) | 8091.37(6500.95,10055.72) | 4258024(3294408,5464125) | 6617.22(5130.80,8476.26) | -0.67(-0.74,-0.60) | <0.001 |
| Paraguay | 154923(121170,201046) | 7391.45(5756.17,9626.18) | 179309(131806,241113) | 6728.94(4937.81,9054.62) | -0.17(-0.32,-0.02) | 0.03 |
| Algeria | 1930078(1322268,2375578) | 14191.03(9735.96,17478.97) | 1205658(941184,1519799) | 7336.73(5713.98,9266.10) | -2.10(-2.16,-2.03) | <0.001 |
| Bahrain | 20328(16937,23929) | 9878.78(8193.73,11709.87) | 23702(18494,30136) | 5782.81(4541.73,7319.04) | -1.77(-1.89,-1.65) | <0.001 |
| Egypt | 6058119(4215938,7367187) | 20227.79(14196.42,24597.64) | 3475106(2781521,4332842) | 7491.58(5988.54,9347.53) | -3.17(-3.33,-3.01) | <0.001 |
| Iran (Islamic Republic of) | 4575219(3278868,5550273) | 14439.58(10358.33,17544.03) | 1464969(1145819,1860932) | 5684.49(4452.82,7209.83) | -3.00(-3.18,-2.82) | <0.001 |
| Iraq | 1373340(1007155,1699067) | 12636.95(9318.84,15642.24) | 1203654(932157,1558430) | 6963.40(5406.60,9010.01) | -1.94(-2.09,-1.79) | <0.001 |
| Jordan | 235298(186489,284339) | 10963.55(8686.89,13265.85) | 314267(243349,404900) | 6487.05(5060.07,8321.16) | -1.71(-1.78,-1.65) | <0.001 |
| Kuwait | 55118(45913,66187) | 7908.54(6550.69,9549.34) | 63492(49786,80793) | 5897.46(4647.50,7472.63) | -0.51(-1.40,0.38) | 0.26 |
| Lebanon | 109199(76814,140298) | 7993.82(5648.24,10272.23) | 94702(69910,124752) | 5662.52(4187.20,7462.30) | -1.00(-1.12,-0.87) | <0.001 |
| Libya | 302644(234187,374421) | 13018.66(10075.78,16106.72) | 194521(152003,247033) | 10063.86(7876.91,12767.15) | -0.81(-1.13,-0.49) | <0.001 |
| Morocco | 1370191(1109757,1690880) | 10750.24(8698.92,13274.91) | 765503(578022,1020972) | 5903.30(4462.93,7884.07) | -1.94(-2.05,-1.83) | <0.001 |
| Palestine | 143460(109265,173735) | 11130.92(8502.05,13547.39) | 153380(116754,197763) | 6422.11(4895.83,8275.50) | -1.68(-1.87,-1.50) | <0.001 |
| Oman | 105223(82264,130923) | 9834.94(7672.40,12271.30) | 84237(65508,107328) | 5808.84(4496.04,7414.81) | -1.62(-1.71,-1.53) | <0.001 |
| Qatar | 12789(9897,15842) | 7953.86(6155.59,9896.44) | 28308(21808,36428) | 4920.28(3775.55,6357.74) | -1.48(-1.62,-1.35) | <0.001 |
| Saudi Arabia | 978609(703544,1247670) | 11525.70(8312.90,14688.25) | 508567(385271,662870) | 4912.71(3729.64,6400.80) | -2.71(-2.77,-2.64) | <0.001 |
| Syrian Arab Republic | 986936(750900,1205998) | 13063.35(9963.93,15968.46) | 377676(292078,473638) | 6731.29(5246.66,8421.91) | -2.20(-2.41,-1.99) | <0.001 |
| Tunisia | 497547(342805,622911) | 12577.37(8656.91,15746.70) | 224348(172390,285600) | 6315.43(4865.89,8020.70) | -2.14(-2.29,-1.99) | <0.001 |
| Turkey | 4480395(3005317,5536529) | 17178.67(11480.84,21223.17) | 1634598(1273233,2060593) | 6745.70(5286.86,8466.56) | -2.95(-3.09,-2.80) | <0.001 |
| United Arab Emirates | 65404(51856,81278) | 9005.45(7132.44,11209.12) | 87429(66860,110824) | 5259.11(4028.60,6657.01) | -1.84(-2.14,-1.55) | <0.001 |
| Yemen | 1909213(1063999,2469235) | 19853.97(11297.70,25692.29) | 1865137(1415841,2383221) | 10752.70(8158.70,13743.04) | -1.99(-2.11,-1.88) | <0.001 |
| Afghanistan | 1525309(745060,1991082) | 25066.93(12504.04,32684.51) | 2560824(1817278,3267564) | 13608.78(9732.87,17339.39) | -1.83(-1.96,-1.69) | <0.001 |
| Bangladesh | 7330935(5200908,9505316) | 11410.07(8165.68,14749.98) | 3423526(2576858,4566965) | 5653.21(4229.63,7587.52) | -2.21(-2.31,-2.10) | <0.001 |
| Bhutan | 30888(20010,42454) | 8909.83(5779.88,12238.21) | 14219(10479,18784) | 5605.68(4109.63,7416.35) | -1.45(-1.84,-1.05) | <0.001 |
| India | 34761314(26986742,43215461) | 8399.50(6531.47,10442.30) | 27186178(21169575,35022784) | 5524.41(4314.11,7114.28) | -1.38(-1.45,-1.30) | <0.001 |
| Nepal | 960004(768521,1181342) | 8806.17(7040.76,10855.79) | 639781(481283,875561) | 5049.08(3794.05,6948.25) | -1.67(-1.76,-1.59) | <0.001 |
| Pakistan | 5587432(4432987,7029185) | 8845.81(7020.87,11123.04) | 7709223(6203414,9684256) | 7076.35(5693.78,8888.27) | -0.76(-0.91,-0.60) | <0.001 |
| Angola | 830047(492932,1236292) | 12812.29(7836.23,18747.22) | 1369417(1048964,1771343) | 7259.53(5569.32,9366.47) | -1.89(-1.97,-1.81) | <0.001 |
| Central African Republic | 219856(144848,313956) | 13137.24(8841.79,18522.36) | 295915(217924,400927) | 9989.58(7378.31,13498.57) | -0.89(-0.99,-0.78) | <0.001 |
| Congo | 129774(93173,173492) | 9457.86(6823.48,12593.65) | 156262(122309,197301) | 6362.75(4979.70,8031.95) | -1.36(-1.48,-1.24) | <0.001 |
| Democratic Republic of the Congo | 2781523(1952988,3730284) | 11540.43(8203.00,15357.63) | 3262574(2508217,4242157) | 6799.93(5231.57,8831.69) | -1.77(-1.83,-1.71) | <0.001 |
| Equatorial Guinea | 29187(20985,39594) | 10998.32(8007.96,14775.46) | 47970(35040,64350) | 6305.75(4592.44,8491.34) | -1.82(-1.96,-1.68) | <0.001 |
| Gabon | 42529(31897,56254) | 8090.80(6090.48,10650.04) | 50737(38271,66976) | 6174.11(4655.50,8166.25) | -0.94(-1.04,-0.85) | <0.001 |
| Burundi | 455219(298148,611307) | 13026.64(8788.58,17274.15) | 556945(433368,720729) | 7555.47(5891.16,9746.31) | -1.86(-2.00,-1.72) | <0.001 |
| Comoros | 32532(21437,45763) | 11510.47(7665.23,16066.05) | 24160(18348,31951) | 7774.14(5892.79,10304.30) | -1.32(-1.75,-0.88) | <0.001 |
| Djibouti | 21115(14822,29543) | 9156.64(6453.77,12769.61) | 34571(25227,46156) | 6534.65(4771.58,8715.97) | -0.96(-1.16,-0.76) | <0.001 |
| Eritrea | 223272(144439,318506) | 10660.76(7042.34,15010.16) | 258107(191785,349219) | 7926.43(5902.36,10691.78) | -0.95(-1.05,-0.86) | <0.001 |
| Ethiopia | 4314352(2576331,6420854) | 13431.67(8298.98,19617.31) | 4226287(3268538,5591978) | 7318.26(5663.11,9672.29) | -1.91(-1.99,-1.83) | <0.001 |
| Kenya | 1077634(811138,1494774) | 7507.24(5677.74,10315.86) | 1414137(1097335,1799856) | 5806.37(4497.56,7407.31) | -0.80(-0.86,-0.73) | <0.001 |
| Madagascar | 902443(647035,1240376) | 12464.79(9035.50,16947.96) | 1249522(971322,1606318) | 8384.36(6520.07,10773.60) | -1.31(-1.40,-1.22) | <0.001 |
| Malawi | 925606(568937,1325456) | 14536.35(9196.62,20531.02) | 824469(645172,1052837) | 7873.85(6159.02,10062.80) | -2.02(-2.19,-1.85) | <0.001 |
| Mauritius | 27447(22462,33001) | 6477.02(5326.50,7746.63) | 18013(14350,22425) | 5988.26(4806.93,7375.66) | -0.26(-0.52,0.01) | 0.06 |
| Mozambique | 1205411(744345,1811743) | 14481.35(9106.62,21513.94) | 1520973(1157984,2065265) | 8452.12(6446.15,11429.18) | -1.61(-1.82,-1.40) | <0.001 |
| Rwanda | 616807(417077,859736) | 13807.89(9535.10,18964.03) | 490188(390318,620708) | 7596.87(6049.16,9620.17) | -1.92(-2.23,-1.60) | <0.001 |
| Seychelles | 1777(1445,2204) | 5699.72(4643.81,7054.63) | 1548(1177,2017) | 5111.43(3894.59,6642.53) | -0.32(-0.73,0.09) | 0.13 |
| Somalia | 592519(376024,883626) | 11469.30(7438.43,16833.92) | 1130408(810701,1607652) | 8405.49(6091.12,11780.22) | -1.10(-1.21,-1.00) | <0.001 |
| United Republic of Tanzania | 2146339(1380570,3005464) | 13137.05(8617.62,18215.13) | 2863129(2230504,3680862) | 9131.90(7119.35,11726.55) | -1.22(-1.32,-1.12) | <0.001 |
| Uganda | 1228471(854053,1750316) | 10551.26(7443.33,14811.02) | 2028833(1611098,2606909) | 8000.43(6353.80,10261.97) | -1.07(-1.34,-0.80) | <0.001 |
| Zambia | 639170(405340,941618) | 12434.01(8040.46,18084.04) | 843464(649346,1105302) | 8063.03(6211.93,10549.35) | -1.36(-1.49,-1.23) | <0.001 |
| Botswana | 42630(33427,53020) | 5750.33(4509.46,7158.40) | 52170(39585,67728) | 5701.91(4324.90,7408.50) | -0.01(-0.11,0.09) | 0.88 |
| Lesotho | 51417(37788,65521) | 6102.36(4504.86,7767.71) | 55449(41649,71824) | 6657.25(4976.91,8650.57) | 0.30(0.16,0.43) | <0.001 |
| Namibia | 45808(34826,57207) | 5903.52(4500.28,7380.12) | 57873(43657,74812) | 5414.37(4082.66,7003.68) | -0.18(-0.28,-0.08) | <0.001 |
| South Africa | 1109583(911851,1348083) | 6251.82(5135.77,7598.18) | 1068860(830437,1349156) | 5406.13(4199.71,6821.60) | -0.47(-0.56,-0.38) | <0.001 |
| Eswatini | 31959(24476,39127) | 6585.05(5063.20,8068.85) | 34025(25936,43216) | 6373.15(4857.08,8098.47) | -0.04(-0.11,0.03) | 0.24 |
| Zimbabwe | 323851(257128,401477) | 5341.52(4241.04,6638.04) | 517478(404803,650137) | 6502.01(5089.04,8164.87) | 0.67(0.40,0.94) | <0.001 |
| Benin | 448972(261253,592216) | 13864.75(8385.26,18161.80) | 755483(576282,968621) | 9586.74(7333.41,12285.32) | -1.21(-1.39,-1.03) | <0.001 |
| Burkina Faso | 932158(584143,1311423) | 14710.65(9380.82,20594.78) | 1567825(1130991,2277128) | 11332.54(8215.86,16314.75) | -0.81(-0.92,-0.70) | <0.001 |
| Cameroon | 683202(436753,859683) | 10436.81(6855.09,13126.38) | 1380128(1054598,1733591) | 8072.50(6176.14,10143.18) | -0.90(-1.06,-0.74) | <0.001 |
| Cabo Verde | 15225(11151,19358) | 7551.04(5556.82,9608.85) | 9677(7487,12414) | 4971.16(3844.60,6385.17) | -1.32(-1.43,-1.22) | <0.001 |
| Chad | 432091(258130,590388) | 10780.44(6685.64,14592.90) | 1099608(725744,1452983) | 9341.35(6297.70,12276.86) | -0.45(-0.56,-0.34) | <0.001 |
| C么te d'Ivoire | 802451(537249,1006102) | 10576.59(7234.54,13264.76) | 1204494(964197,1503736) | 8141.41(6509.33,10173.71) | -0.75(-0.88,-0.62) | <0.001 |
| Gambia | 57328(40759,77474) | 9498.39(6842.40,12763.07) | 90835(70390,122205) | 7135.52(5531.89,9583.77) | -0.85(-1.17,-0.53) | <0.001 |
| Ghana | 900363(668255,1117006) | 10278.37(7698.72,12741.52) | 1194659(921387,1558707) | 7288.62(5626.90,9499.61) | -1.16(-1.27,-1.05) | <0.001 |
| Guinea | 608961(344153,802001) | 15790.32(9263.01,20687.35) | 766323(585797,956215) | 9928.39(7598.28,12397.00) | -1.46(-1.61,-1.31) | <0.001 |
| Guinea-Bissau | 90174(57443,120328) | 14206.37(9245.85,18858.05) | 92261(70913,121743) | 8137.43(6264.96,10709.87) | -1.79(-1.94,-1.64) | <0.001 |
| Liberia | 260947(150976,338766) | 16608.67(9903.57,21510.59) | 227786(175163,298351) | 8150.88(6269.04,10672.04) | -2.36(-2.69,-2.04) | <0.001 |
| Mali | 913370(592911,1176701) | 15827.34(10496.78,20334.41) | 1469889(1122500,1897644) | 9677.34(7410.23,12483.43) | -1.54(-1.88,-1.21) | <0.001 |
| Mauritania | 105905(69538,136256) | 8770.98(5897.19,11251.28) | 140184(109233,177187) | 6019.54(4688.95,7610.48) | -1.27(-1.37,-1.16) | <0.001 |
| Niger | 782770(455155,1023739) | 14059.32(8484.04,18297.95) | 1308363(977736,1669410) | 7929.24(5955.14,10114.67) | -1.72(-1.96,-1.48) | <0.001 |
| Nigeria | 6461351(4010339,8339903) | 12112.52(7706.56,15598.29) | 12694993(9650738,15871221) | 9689.88(7376.11,12120.99) | -0.74(-0.80,-0.67) | <0.001 |
| Sao Tome and Principe | 8318(5995,10527) | 11527.05(8325.02,14601.12) | 5868(4498,7870) | 5789.63(4428.85,7804.25) | -2.16(-2.63,-1.70) | <0.001 |
| Senegal | 549698(370374,703593) | 11315.69(7780.14,14438.23) | 584638(464034,733870) | 7163.17(5683.80,8991.67) | -1.45(-1.87,-1.03) | <0.001 |
| Sierra Leone | 442598(240419,567141) | 16993.99(9598.57,21718.56) | 477105(363245,602445) | 10126.43(7725.43,12785.90) | -1.71(-1.99,-1.44) | <0.001 |
| Togo | 267775(187469,339040) | 11661.26(8270.84,14748.38) | 332505(257284,425945) | 7950.53(6151.14,10177.39) | -1.27(-1.43,-1.12) | <0.001 |
| American Samoa | 1296(1045,1588) | 5345.00(4288.73,6576.93) | 977(767,1249) | 5082.74(3980.63,6504.53) | -0.08(-0.17,0.01) | 0.07 |
| Bermuda | 933(735,1182) | 5792.74(4567.48,7337.62) | 579(436,765) | 4928.98(3729.41,6482.04) | -0.48(-0.56,-0.40) | <0.001 |
| Cook Islands | 408(312,522) | 4719.94(3607.00,6040.61) | 210(157,280) | 3897.92(2914.52,5206.27) | -0.33(-0.51,-0.15) | <0.001 |
| Greenland | 1650(1312,2054) | 8917.23(7058.19,11125.40) | 914(692,1190) | 5900.68(4471.39,7671.86) | -1.18(-1.34,-1.02) | <0.001 |
| Guam | 2813(2283,3486) | 5126.73(4147.54,6370.10) | 2393(1858,3077) | 4855.64(3771.14,6242.03) | -0.44(-0.64,-0.24) | <0.001 |
| Monaco | 293(224,376) | 5921.77(4532.60,7603.52) | 382(293,489) | 5463.21(4215.71,6969.87) | 0.06(-0.12,0.25) | 0.48 |
| Nauru | 403(302,509) | 7606.69(5752.50,9580.05) | 366(281,469) | 7120.86(5467.77,9122.79) | -0.28(-0.37,-0.19) | <0.001 |
| Niue | 62(48,77) | 6209.74(4810.15,7755.96) | 63(55,72) | 12743.23(11196.10,14517.39) | 4.13(2.94,5.33) | <0.001 |
| Northern Mariana Islands | 745(571,959) | 4480.53(3430.43,5772.01) | 643(492,832) | 4160.92(3200.28,5363.07) | -0.31(-0.67,0.05) | 0.09 |
| Palau | 413(299,533) | 6788.26(4856.68,8786.74) | 251(197,317) | 5632.79(4405.10,7111.14) | -0.58(-0.65,-0.51) | <0.001 |
| Puerto Rico | 85340(69925,105037) | 6472.88(5343.86,7906.91) | 34532(26131,44837) | 5193.57(3988.32,6668.84) | -0.73(-0.92,-0.53) | <0.001 |
| Saint Kitts and Nevis | 1346(1116,1627) | 7427.01(6173.54,8952.47) | 915(716,1160) | 6451.38(5064.16,8179.25) | -0.44(-0.57,-0.31) | <0.001 |
| San Marino | 353(267,455) | 5680.22(4338.66,7248.64) | 321(235,424) | 4676.96(3435.33,6168.31) | -0.57(-0.66,-0.48) | <0.001 |
| Tokelau | 47(36,59) | 6423.07(4882.54,8050.07) | 63(53,76) | 13826.31(11743.56,16843.01) | 6.12(3.97,8.26) | <0.001 |
| Tuvalu | 487(291,647) | 10207.01(6400.85,13422.20) | 276(214,353) | 5582.48(4321.15,7154.34) | -1.88(-1.97,-1.79) | <0.001 |
| United States Virgin Islands | 2740(2170,3394) | 6547.52(5189.37,8101.09) | 926(700,1226) | 4964.67(3749.01,6558.21) | -0.88(-1.01,-0.76) | <0.001 |
| South Sudan | 459735(281673,676896) | 12963.71(8066.38,18930.12) | 667973(460818,949391) | 11881.73(8224.15,16835.01) | -0.32(-0.44,-0.19) | <0.001 |
| Sudan | 3042849(1742148,3863048) | 24824.73(14434.87,31495.00) | 2369030(1852686,3001138) | 11219.34(8774.23,14213.24) | -2.54(-2.59,-2.49) | <0.001 |

Supplementary Table 7: Results of the 2021 Global Analysis of the Health Frontiers of NCDs among Children and Adolescents in 204 Countries.

|  |  | **ASPR** | | | **ASIR** | | | **ASMR** | | | **ASDR** | | |
| --- | --- | --- | --- | --- | --- | --- | --- | --- | --- | --- | --- | --- | --- |
| **Location** | **SDI** | **val** | **frontier** | **eff_diff** | **val** | **frontier** | **eff_diff** | **val** | **frontier** | **eff_diff** | **val** | **frontier** | **eff_diff** |
| China | 0.722 | 76881.196 | 70397.369 | 6483.826 | 130883.622 | 110814.880 | 20068.742 | 16.209 | 10.994 | 5.215 | 4009.254 | 3868.618 | 140.6361 |
| Democratic People's Republic of Korea | 0.570 | 74019.249 | 73682.366 | 336.883 | 120294.522 | 111865.291 | 8429.232 | 23.807 | 21.964 | 1.844 | 4763.356 | 4617.341 | 146.0151 |
| Taiwan (Province of China) | 0.875 | 75189.373 | 67549.215 | 7640.159 | 123280.412 | 105264.329 | 18016.083 | 12.071 | 7.087 | 4.984 | 3987.132 | 3499.775 | 487.3573 |
| Cambodia | 0.474 | 83894.546 | 73814.217 | 10080.329 | 153908.873 | 116239.559 | 37669.313 | 54.518 | 24.658 | 29.861 | 7861.667 | 4894.01 | 2967.657 |
| Indonesia | 0.657 | 80087.084 | 73461.433 | 6625.651 | 157658.515 | 110941.667 | 46716.848 | 36.589 | 16.342 | 20.247 | 6036.825 | 4162.949 | 1873.877 |
| Lao People's Democratic Republic | 0.489 | 80850.960 | 73817.976 | 7032.984 | 150429.871 | 116199.036 | 34230.834 | 64.403 | 24.569 | 39.834 | 8653.99 | 4891.203 | 3762.787 |
| Malaysia | 0.743 | 76667.929 | 69935.827 | 6732.102 | 144451.548 | 110227.237 | 34224.311 | 17.756 | 9.454 | 8.303 | 4618.978 | 3685.365 | 933.613 |
| Maldives | 0.651 | 81640.514 | 73582.845 | 8057.669 | 150411.619 | 110985.781 | 39425.838 | 24.226 | 16.611 | 7.614 | 5324.657 | 4170.284 | 1154.373 |
| Myanmar | 0.534 | 80217.663 | 73816.357 | 6401.306 | 148495.179 | 115990.500 | 32504.680 | 71.545 | 22.986 | 48.559 | 9317.876 | 4827.848 | 4490.028 |
| Philippines | 0.651 | 78940.584 | 73612.860 | 5327.724 | 156628.549 | 110940.083 | 45688.466 | 36.235 | 16.546 | 19.688 | 6269.146 | 4180.399 | 2088.747 |
| Sri Lanka | 0.702 | 80615.583 | 71384.008 | 9231.575 | 150313.447 | 110922.995 | 39390.451 | 16.944 | 13.326 | 3.618 | 4504.747 | 4057.825 | 446.9225 |
| Thailand | 0.683 | 80784.519 | 72552.191 | 8232.328 | 148168.702 | 110766.272 | 37402.430 | 19.970 | 14.845 | 5.126 | 4754.372 | 4097.997 | 656.3755 |
| Timor-Leste | 0.445 | 82069.618 | 73819.690 | 8249.928 | 150341.158 | 116240.446 | 34100.712 | 51.365 | 24.802 | 26.563 | 7517.6 | 4890.364 | 2627.237 |
| Viet Nam | 0.628 | 78413.604 | 73696.333 | 4717.272 | 149668.751 | 110901.528 | 38767.223 | 15.621 | 15.621 | 0.000 | 4136 | 4127.521 | 8.479071 |
| Fiji | 0.675 | 79363.091 | 72896.925 | 6466.166 | 158196.544 | 110826.602 | 47369.942 | 43.838 | 15.130 | 28.708 | 6783.269 | 4145.274 | 2637.996 |
| Kiribati | 0.527 | 81260.604 | 73815.600 | 7445.004 | 155280.222 | 116204.408 | 39075.814 | 56.557 | 23.791 | 32.765 | 7788.177 | 4830.386 | 2957.791 |
| Marshall Islands | 0.574 | 80272.301 | 73702.311 | 6569.990 | 154958.696 | 111945.876 | 43012.820 | 35.625 | 21.757 | 13.868 | 5952.082 | 4604.376 | 1347.706 |
| Micronesia (Federated States of) | 0.588 | 80284.518 | 73687.294 | 6597.224 | 154456.722 | 111119.667 | 43337.055 | 29.354 | 19.932 | 9.423 | 5413.42 | 4451.009 | 962.4111 |
| Papua New Guinea | 0.418 | 82198.187 | 73862.577 | 8335.611 | 154796.208 | 116192.525 | 38603.682 | 72.282 | 27.742 | 44.541 | 9281.659 | 5194.009 | 4087.651 |
| Samoa | 0.593 | 80031.178 | 73689.270 | 6341.908 | 154370.275 | 110947.235 | 43423.040 | 26.540 | 19.346 | 7.193 | 5202.427 | 4409.266 | 793.1608 |
| Solomon Islands | 0.429 | 81800.415 | 73817.410 | 7983.005 | 153966.535 | 116201.669 | 37764.866 | 33.617 | 25.605 | 8.012 | 5813.899 | 5057.272 | 756.627 |
| Tonga | 0.626 | 79858.283 | 73693.942 | 6164.341 | 154900.676 | 110884.318 | 44016.358 | 21.264 | 17.197 | 4.067 | 4794.601 | 4181.296 | 613.3052 |
| Vanuatu | 0.473 | 80130.204 | 73816.320 | 6313.884 | 151516.420 | 116252.751 | 35263.669 | 34.020 | 24.785 | 9.236 | 5796.572 | 4888.812 | 907.7601 |
| Armenia | 0.702 | 77387.654 | 71533.420 | 5854.234 | 144077.404 | 110908.394 | 33169.010 | 22.044 | 13.298 | 8.745 | 5105.244 | 4063.279 | 1041.965 |
| Azerbaijan | 0.695 | 77698.831 | 71997.554 | 5701.277 | 140033.438 | 110906.636 | 29126.802 | 36.287 | 14.038 | 22.249 | 6284.531 | 4090 | 2194.531 |
| Georgia | 0.732 | 78454.983 | 70065.121 | 8389.861 | 134187.555 | 110830.177 | 23357.378 | 19.289 | 10.546 | 8.742 | 4801 | 3834.252 | 966.7483 |
| Kazakhstan | 0.725 | 76401.343 | 70467.197 | 5934.146 | 139758.688 | 110951.031 | 28807.657 | 27.174 | 11.136 | 16.038 | 5448.601 | 3875.3 | 1573.301 |
| Kyrgyzstan | 0.604 | 78078.383 | 73691.740 | 4386.643 | 142779.969 | 110859.789 | 31920.180 | 26.722 | 18.928 | 7.794 | 5474.017 | 4355.701 | 1118.316 |
| Mongolia | 0.618 | 78023.511 | 73688.676 | 4334.836 | 140286.831 | 110868.939 | 29417.892 | 29.149 | 18.023 | 11.127 | 5601.04 | 4261.404 | 1339.636 |
| Tajikistan | 0.542 | 78088.242 | 73817.607 | 4270.635 | 138875.560 | 114164.154 | 24711.406 | 36.489 | 23.082 | 13.407 | 6285.606 | 4824.521 | 1461.085 |
| Turkmenistan | 0.682 | 77487.430 | 72766.523 | 4720.907 | 139313.482 | 110892.939 | 28420.543 | 55.876 | 14.691 | 41.185 | 7972.008 | 4114.178 | 3857.829 |
| Uzbekistan | 0.663 | 77591.937 | 73301.642 | 4290.295 | 139189.302 | 110791.004 | 28398.297 | 35.154 | 16.046 | 19.109 | 6188.69 | 4157.826 | 2030.864 |
| Albania | 0.707 | 76622.380 | 70859.394 | 5762.986 | 141036.510 | 110910.059 | 30126.451 | 26.385 | 12.408 | 13.978 | 5507.27 | 3988.188 | 1519.081 |
| Bosnia and Herzegovina | 0.723 | 78046.199 | 70388.059 | 7658.140 | 141311.198 | 111033.644 | 30277.554 | 12.152 | 10.857 | 1.295 | 4281.662 | 3888.945 | 392.7164 |
| Bulgaria | 0.768 | 77735.943 | 69420.584 | 8315.358 | 146512.207 | 108288.157 | 38224.050 | 18.855 | 7.837 | 11.018 | 4917.648 | 3616.228 | 1301.42 |
| Croatia | 0.798 | 76930.660 | 68479.628 | 8451.032 | 137708.491 | 105902.029 | 31806.462 | 12.752 | 7.852 | 4.900 | 4252.013 | 3583.35 | 668.6632 |
| Czechia | 0.828 | 76682.741 | 67724.086 | 8958.655 | 149191.761 | 105780.375 | 43411.386 | 7.579 | 7.261 | 0.318 | 3855.419 | 3564.607 | 290.8119 |
| Hungary | 0.791 | 76736.329 | 68803.009 | 7933.320 | 142169.379 | 106552.101 | 35617.278 | 11.684 | 8.365 | 3.320 | 4223.185 | 3606.566 | 616.6189 |
| North Macedonia | 0.751 | 76985.833 | 69815.040 | 7170.794 | 141750.374 | 109583.891 | 32166.483 | 13.314 | 8.684 | 4.630 | 4330.782 | 3662.058 | 668.7238 |
| Montenegro | 0.796 | 76809.749 | 68643.712 | 8166.036 | 141977.764 | 106101.821 | 35875.943 | 8.559 | 7.610 | 0.949 | 3908.044 | 3543.081 | 364.9636 |
| Poland | 0.812 | 78390.713 | 67765.408 | 10625.305 | 144428.335 | 105653.842 | 38774.492 | 12.986 | 7.428 | 5.559 | 4593.19 | 3585.476 | 1007.714 |
| Romania | 0.768 | 79727.811 | 69287.869 | 10439.942 | 150716.075 | 108303.933 | 42412.142 | 15.843 | 8.493 | 7.349 | 4524.726 | 3573.009 | 951.7164 |
| Serbia | 0.792 | 73830.907 | 68733.595 | 5097.312 | 125610.273 | 105971.849 | 19638.423 | 10.832 | 8.004 | 2.829 | 4018.52 | 3583.99 | 434.5298 |
| Slovakia | 0.811 | 76520.813 | 67669.023 | 8851.790 | 143982.630 | 105490.298 | 38492.332 | 15.386 | 7.742 | 7.644 | 4509.023 | 3520.877 | 988.1461 |
| Slovenia | 0.842 | 76374.332 | 67539.507 | 8834.825 | 148332.972 | 105426.582 | 42906.390 | 7.495 | 7.152 | 0.343 | 3798.929 | 3569.253 | 229.6761 |
| Belarus | 0.784 | 75617.599 | 68989.211 | 6628.388 | 145971.783 | 106998.437 | 38973.347 | 12.405 | 7.589 | 4.816 | 4246.625 | 3533.285 | 713.3401 |
| Estonia | 0.845 | 74742.974 | 67600.642 | 7142.332 | 140816.283 | 105477.940 | 35338.343 | 11.081 | 7.138 | 3.943 | 4221.5 | 3541.181 | 680.3183 |
| Latvia | 0.831 | 73360.167 | 67683.128 | 5677.039 | 141649.399 | 105854.066 | 35795.333 | 10.836 | 7.479 | 3.358 | 3991.92 | 3609.701 | 382.2195 |
| Lithuania | 0.856 | 77241.289 | 67487.510 | 9753.779 | 151839.696 | 105397.807 | 46441.888 | 12.803 | 7.381 | 5.421 | 4343.137 | 3566.498 | 776.6398 |
| Republic of Moldova | 0.732 | 75940.248 | 70141.764 | 5798.484 | 143779.063 | 110878.449 | 32900.613 | 27.622 | 10.520 | 17.101 | 5446.136 | 3825.694 | 1620.442 |
| Russian Federation | 0.809 | 76487.529 | 67787.897 | 8699.633 | 151669.476 | 105455.157 | 46214.320 | 15.600 | 7.626 | 7.975 | 4526.65 | 3565.167 | 961.4835 |
| Ukraine | 0.761 | 75963.172 | 69650.465 | 6312.706 | 153205.367 | 108601.153 | 44604.214 | 19.720 | 8.325 | 11.395 | 4783.67 | 3653.459 | 1130.21 |
| Brunei Darussalam | 0.810 | 72343.982 | 67731.940 | 4612.042 | 133888.308 | 105574.562 | 28313.746 | 30.394 | 7.603 | 22.791 | 6066.754 | 3538.428 | 2528.325 |
| Japan | 0.871 | 70377.907 | 67490.449 | 2887.458 | 123902.577 | 105437.999 | 18464.578 | 9.846 | 7.274 | 2.572 | 4441.794 | 3529.784 | 912.0094 |
| Republic of Korea | 0.887 | 75063.967 | 67627.434 | 7436.533 | 141220.734 | 105377.397 | 35843.337 | 7.905 | 7.130 | 0.774 | 4145.222 | 3551.149 | 594.073 |
| Singapore | 0.856 | 69517.789 | 67566.741 | 1951.048 | 134976.724 | 105339.244 | 29637.480 | 8.710 | 7.278 | 1.431 | 4037.258 | 3561.401 | 475.8567 |
| Australia | 0.844 | 75857.751 | 67571.854 | 8285.898 | 151601.775 | 105376.183 | 46225.592 | 10.877 | 7.443 | 3.434 | 4910.693 | 3553.459 | 1357.235 |
| New Zealand | 0.849 | 70357.433 | 67495.171 | 2862.262 | 129683.613 | 105451.987 | 24231.625 | 14.872 | 7.335 | 7.537 | 5313.063 | 3526.013 | 1787.051 |
| Andorra | 0.869 | 74529.958 | 67485.982 | 7043.977 | 132943.232 | 105759.338 | 27183.894 | 6.698 | 6.693 | 0.005 | 4581.224 | 3550.338 | 1030.886 |
| Austria | 0.854 | 74026.027 | 67506.836 | 6519.192 | 132634.191 | 105610.472 | 27023.720 | 11.470 | 7.291 | 4.179 | 4879.095 | 3532.575 | 1346.519 |
| Belgium | 0.854 | 72638.767 | 67502.998 | 5135.769 | 122331.447 | 105519.450 | 16811.997 | 13.138 | 7.358 | 5.781 | 5093.04 | 3569.066 | 1523.975 |
| Cyprus | 0.836 | 78023.152 | 67650.452 | 10372.700 | 133444.734 | 105390.060 | 28054.673 | 7.315 | 7.104 | 0.211 | 4542.319 | 3549.425 | 992.8944 |
| Denmark | 0.896 | 71564.645 | 67550.100 | 4014.545 | 125705.838 | 105635.492 | 20070.347 | 10.886 | 7.156 | 3.730 | 4870.395 | 3540.555 | 1329.841 |
| Finland | 0.860 | 73551.133 | 67560.043 | 5991.089 | 128390.860 | 105176.329 | 23214.531 | 11.289 | 7.352 | 3.936 | 4811.536 | 3537.53 | 1274.006 |
| France | 0.838 | 73272.280 | 67468.638 | 5803.642 | 126669.776 | 105523.290 | 21146.486 | 11.520 | 7.498 | 4.022 | 5222.899 | 3531.953 | 1690.946 |
| Germany | 0.903 | 73056.942 | 67536.981 | 5519.961 | 139721.171 | 105468.406 | 34252.765 | 12.080 | 7.045 | 5.035 | 5245.258 | 3579.795 | 1665.463 |
| Greece | 0.792 | 73813.439 | 68829.810 | 4983.629 | 123014.197 | 106023.908 | 16990.289 | 12.709 | 8.144 | 4.565 | 5307.004 | 3539.795 | 1767.209 |
| Iceland | 0.876 | 75597.324 | 67486.860 | 8110.464 | 133408.986 | 105557.349 | 27851.637 | 10.481 | 7.211 | 3.269 | 4833.511 | 3524.898 | 1308.613 |
| Ireland | 0.874 | 75749.678 | 67592.168 | 8157.511 | 134143.119 | 105461.678 | 28681.441 | 12.272 | 7.084 | 5.188 | 5400.202 | 3544.851 | 1855.351 |
| Israel | 0.809 | 77678.222 | 67808.866 | 9869.356 | 144139.876 | 105447.685 | 38692.191 | 10.407 | 7.838 | 2.569 | 4536.67 | 3530.282 | 1006.388 |
| Italy | 0.806 | 77574.480 | 67934.361 | 9640.120 | 124236.040 | 105414.283 | 18821.757 | 10.378 | 7.768 | 2.610 | 4976.512 | 3563.413 | 1413.099 |
| Luxembourg | 0.884 | 74367.859 | 67469.213 | 6898.646 | 131689.654 | 105772.249 | 25917.405 | 9.382 | 7.125 | 2.257 | 4822.427 | 3547.108 | 1275.32 |
| Malta | 0.802 | 74342.944 | 68088.736 | 6254.209 | 129732.360 | 105549.848 | 24182.512 | 22.024 | 7.901 | 14.123 | 5858.377 | 3573.896 | 2284.481 |
| Netherlands | 0.888 | 74001.200 | 67562.011 | 6439.190 | 129452.515 | 105168.199 | 24284.316 | 12.437 | 7.018 | 5.419 | 5066.028 | 3563.026 | 1503.002 |
| Norway | 0.916 | 74204.479 | 67547.201 | 6657.278 | 137503.910 | 105671.415 | 31832.495 | 10.443 | 6.998 | 3.445 | 4834.386 | 3519.813 | 1314.573 |
| Portugal | 0.744 | 75057.403 | 69900.735 | 5156.668 | 130804.249 | 109938.035 | 20866.214 | 10.894 | 9.003 | 1.891 | 5415.308 | 3687.979 | 1727.329 |
| Spain | 0.769 | 73051.175 | 69351.698 | 3699.477 | 124053.194 | 108436.650 | 15616.544 | 10.855 | 8.114 | 2.741 | 5046.912 | 3634.095 | 1412.817 |
| Sweden | 0.887 | 75141.783 | 67571.842 | 7569.941 | 142627.751 | 105388.723 | 37239.028 | 9.730 | 7.308 | 2.422 | 4741.046 | 3540.875 | 1200.171 |
| Switzerland | 0.933 | 72390.948 | 67510.735 | 4880.214 | 125346.824 | 105661.116 | 19685.708 | 13.014 | 7.167 | 5.848 | 5151.503 | 3549.787 | 1601.716 |
| United Kingdom | 0.859 | 73996.488 | 67514.371 | 6482.117 | 122504.744 | 105559.575 | 16945.168 | 13.014 | 7.427 | 5.587 | 5229.293 | 3539.547 | 1689.746 |
| Argentina | 0.723 | 75990.158 | 70578.000 | 5412.158 | 137274.460 | 110753.766 | 26520.694 | 26.210 | 11.274 | 14.936 | 6008.316 | 3892.612 | 2115.704 |
| Chile | 0.772 | 76354.825 | 69393.727 | 6961.098 | 143492.278 | 108258.011 | 35234.267 | 17.765 | 7.903 | 9.862 | 5436.504 | 3552.034 | 1884.469 |
| Uruguay | 0.719 | 76428.266 | 70626.222 | 5802.044 | 139806.742 | 110886.934 | 28919.808 | 23.149 | 12.174 | 10.975 | 5739.931 | 3958.946 | 1780.986 |
| Canada | 0.873 | 72308.846 | 67597.669 | 4711.178 | 116533.672 | 105347.571 | 11186.101 | 12.516 | 7.207 | 5.309 | 4843.731 | 3546.665 | 1297.066 |
| United States of America | 0.862 | 76179.261 | 67531.520 | 8647.741 | 127185.150 | 105239.286 | 21945.864 | 16.984 | 7.156 | 9.828 | 5967.727 | 3557.425 | 2410.302 |
| Antigua and Barbuda | 0.750 | 81326.682 | 69757.665 | 11569.017 | 159862.829 | 109749.691 | 50113.137 | 26.561 | 8.752 | 17.809 | 5892.15 | 3657.998 | 2234.153 |
| Bahamas | 0.805 | 82175.120 | 67901.709 | 14273.411 | 160701.370 | 105361.513 | 55339.857 | 28.160 | 7.841 | 20.319 | 6062.77 | 3550.779 | 2511.99 |
| Barbados | 0.747 | 81955.636 | 70017.479 | 11938.157 | 160190.454 | 109951.686 | 50238.768 | 30.320 | 8.595 | 21.725 | 6294.511 | 3670.003 | 2624.508 |
| Belize | 0.610 | 81589.663 | 73694.732 | 7894.931 | 160814.530 | 110899.228 | 49915.303 | 28.563 | 18.384 | 10.179 | 5980.688 | 4328.588 | 1652.1 |
| Cuba | 0.669 | 80229.817 | 73120.855 | 7108.962 | 148047.381 | 111152.438 | 36894.942 | 15.645 | 15.566 | 0.078 | 4969.733 | 4167.104 | 802.6292 |
| Dominica | 0.747 | 81550.458 | 69938.340 | 11612.118 | 159263.094 | 109931.142 | 49331.952 | 57.081 | 9.207 | 47.875 | 8587.98 | 3704.237 | 4883.743 |
| Dominican Republic | 0.619 | 81004.221 | 73688.577 | 7315.644 | 157708.190 | 110836.830 | 46871.360 | 37.403 | 17.494 | 19.909 | 6604.793 | 4233.871 | 2370.922 |
| Grenada | 0.669 | 82560.993 | 72990.884 | 9570.109 | 159762.810 | 110869.548 | 48893.262 | 31.048 | 15.566 | 15.481 | 6412.411 | 4136.529 | 2275.882 |
| Guyana | 0.651 | 81504.365 | 73620.270 | 7884.094 | 160131.442 | 110817.528 | 49313.915 | 40.274 | 16.557 | 23.717 | 7171.094 | 4172.405 | 2998.688 |
| Haiti | 0.448 | 85783.792 | 73817.601 | 11966.191 | 150916.296 | 116257.481 | 34658.815 | 116.662 | 24.883 | 91.779 | 14209.43 | 4899.003 | 9310.427 |
| Jamaica | 0.683 | 83344.377 | 72524.569 | 10819.807 | 159731.950 | 110781.431 | 48950.519 | 25.377 | 14.851 | 10.526 | 5808.986 | 4114.609 | 1694.377 |
| Saint Lucia | 0.673 | 81201.215 | 72958.363 | 8242.852 | 154122.668 | 110772.897 | 43349.771 | 31.633 | 15.472 | 16.162 | 6341.036 | 4153.748 | 2187.288 |
| Saint Vincent and the Grenadines | 0.637 | 81707.474 | 73698.677 | 8008.797 | 148905.770 | 110765.289 | 38140.481 | 34.188 | 16.767 | 17.421 | 6471.085 | 4168.19 | 2302.895 |
| Suriname | 0.634 | 81413.270 | 73694.133 | 7719.136 | 152646.927 | 110964.513 | 41682.414 | 43.764 | 16.569 | 27.195 | 7518.965 | 4173.441 | 3345.524 |
| Trinidad and Tobago | 0.769 | 79575.086 | 69370.733 | 10204.353 | 151158.914 | 108280.101 | 42878.813 | 37.292 | 7.855 | 29.437 | 6809.553 | 3625.239 | 3184.313 |
| Bolivia (Plurinational State of) | 0.599 | 80823.052 | 73692.617 | 7130.435 | 159206.760 | 111004.126 | 48202.635 | 63.437 | 18.898 | 44.539 | 9088.628 | 4363.352 | 4725.276 |
| Ecuador | 0.661 | 80694.825 | 73275.387 | 7419.438 | 157367.626 | 111030.380 | 46337.246 | 34.239 | 16.223 | 18.016 | 6531.853 | 4168.483 | 2363.37 |
| Peru | 0.662 | 81028.224 | 73323.599 | 7704.625 | 159814.578 | 110822.253 | 48992.325 | 30.421 | 16.176 | 14.245 | 6153.726 | 4161.194 | 1992.532 |
| Colombia | 0.655 | 76869.599 | 73497.791 | 3371.808 | 130863.111 | 110704.573 | 20158.538 | 27.677 | 16.476 | 11.200 | 5779.758 | 4165.49 | 1614.268 |
| Costa Rica | 0.700 | 76729.484 | 71394.651 | 5334.833 | 147296.092 | 110899.556 | 36396.536 | 25.803 | 13.472 | 12.331 | 5736.179 | 4058.647 | 1677.532 |
| El Salvador | 0.564 | 77032.510 | 73815.381 | 3217.130 | 145332.535 | 111865.693 | 33466.842 | 26.334 | 22.358 | 3.977 | 5720.327 | 4661.549 | 1058.777 |
| Guatemala | 0.540 | 76437.407 | 73817.759 | 2619.647 | 144970.118 | 114179.684 | 30790.435 | 41.522 | 23.155 | 18.367 | 7013.218 | 4821.358 | 2191.86 |
| Honduras | 0.513 | 76441.322 | 73816.539 | 2624.782 | 139045.324 | 116230.138 | 22815.186 | 35.140 | 23.856 | 11.284 | 6420.694 | 4836.78 | 1583.913 |
| Mexico | 0.665 | 73912.622 | 73322.383 | 590.239 | 146222.985 | 111020.949 | 35202.036 | 36.987 | 15.890 | 21.097 | 6473.099 | 4158.851 | 2314.248 |
| Nicaragua | 0.524 | 75761.468 | 73816.469 | 1944.999 | 133017.879 | 116229.128 | 16788.751 | 27.439 | 23.811 | 3.628 | 5782.746 | 4831.414 | 951.3321 |
| Panama | 0.709 | 76942.101 | 70956.663 | 5985.437 | 136673.617 | 111037.742 | 25635.874 | 38.103 | 12.722 | 25.381 | 6776.117 | 3999.044 | 2777.073 |
| Venezuela (Bolivarian Republic of) | 0.597 | 76627.501 | 73691.623 | 2935.878 | 136985.485 | 110893.772 | 26091.713 | 39.111 | 19.377 | 19.734 | 6712.571 | 4406.926 | 2305.645 |
| Brazil | 0.653 | 81497.111 | 73454.854 | 8042.257 | 175228.502 | 110847.731 | 64380.771 | 29.496 | 16.316 | 13.180 | 6617.225 | 4168.444 | 2448.781 |
| Paraguay | 0.636 | 83733.059 | 73694.451 | 10038.607 | 160740.476 | 110861.519 | 49878.957 | 30.962 | 16.644 | 14.318 | 6728.94 | 4175.421 | 2553.52 |
| Algeria | 0.660 | 78483.258 | 73340.719 | 5142.539 | 121248.817 | 110958.629 | 10290.188 | 43.685 | 16.384 | 27.301 | 7336.725 | 4160.127 | 3176.598 |
| Bahrain | 0.753 | 85301.509 | 69823.121 | 15478.388 | 136334.740 | 109662.898 | 26671.842 | 23.528 | 8.867 | 14.661 | 5782.814 | 3713.921 | 2068.893 |
| Egypt | 0.607 | 79303.342 | 73688.894 | 5614.448 | 124428.054 | 110793.737 | 13634.317 | 45.494 | 18.287 | 27.207 | 7491.579 | 4329.743 | 3161.836 |
| Iran (Islamic Republic of) | 0.697 | 83076.094 | 71475.891 | 11600.203 | 141318.507 | 110919.100 | 30399.406 | 20.602 | 13.414 | 7.189 | 5684.495 | 4065.76 | 1618.735 |
| Iraq | 0.663 | 79975.570 | 73348.730 | 6626.841 | 119524.252 | 110788.554 | 8735.699 | 40.170 | 16.072 | 24.098 | 6963.397 | 4151.527 | 2811.871 |
| Jordan | 0.725 | 79316.821 | 70574.857 | 8741.963 | 133399.151 | 110829.483 | 22569.668 | 33.177 | 11.273 | 21.904 | 6487.046 | 3886.244 | 2600.802 |
| Kuwait | 0.847 | 76885.953 | 67540.384 | 9345.569 | 134025.103 | 105528.368 | 28496.735 | 27.477 | 7.303 | 20.174 | 5897.457 | 3526.672 | 2370.786 |
| Lebanon | 0.745 | 79326.282 | 69989.345 | 9336.937 | 123738.332 | 110161.904 | 13576.428 | 18.418 | 9.687 | 8.731 | 5662.525 | 3718.918 | 1943.607 |
| Libya | 0.726 | 82765.259 | 70431.990 | 12333.270 | 123137.942 | 110887.693 | 12250.249 | 73.260 | 11.230 | 62.029 | 10063.86 | 3874.698 | 6189.165 |
| Morocco | 0.563 | 78286.821 | 73822.163 | 4464.658 | 126427.764 | 112194.903 | 14232.861 | 25.701 | 22.712 | 2.990 | 5903.298 | 4707.687 | 1195.612 |
| Palestine | 0.631 | 78727.110 | 73684.590 | 5042.521 | 122926.301 | 110863.187 | 12063.114 | 30.226 | 16.690 | 13.536 | 6422.105 | 4184.991 | 2237.114 |
| Oman | 0.773 | 84541.622 | 69421.322 | 15120.300 | 136105.515 | 108209.043 | 27896.472 | 23.609 | 7.886 | 15.723 | 5808.845 | 3586.792 | 2222.052 |
| Qatar | 0.847 | 80342.356 | 67605.150 | 12737.206 | 137178.978 | 105341.124 | 31837.854 | 15.267 | 7.317 | 7.950 | 4920.277 | 3539.571 | 1380.707 |
| Saudi Arabia | 0.815 | 83132.884 | 67632.231 | 15500.653 | 133515.750 | 105895.483 | 27620.267 | 15.871 | 7.382 | 8.489 | 4912.707 | 3571.622 | 1341.085 |
| Syrian Arab Republic | 0.623 | 79630.905 | 73698.044 | 5932.861 | 122739.747 | 110817.375 | 11922.372 | 35.717 | 17.518 | 18.200 | 6731.287 | 4235.346 | 2495.942 |
| Tunisia | 0.682 | 80707.011 | 72540.114 | 8166.898 | 135686.049 | 110957.285 | 24728.765 | 27.718 | 14.744 | 12.975 | 6315.428 | 4116.546 | 2198.882 |
| Turkey | 0.713 | 80489.585 | 70402.530 | 10087.055 | 139717.948 | 110834.868 | 28883.080 | 34.193 | 11.968 | 22.225 | 6745.701 | 3953.904 | 2791.797 |
| United Arab Emirates | 0.849 | 81226.698 | 67566.849 | 13659.849 | 123158.209 | 105443.542 | 17714.668 | 17.854 | 7.240 | 10.614 | 5259.106 | 3546.531 | 1712.575 |
| Yemen | 0.450 | 79894.526 | 73816.880 | 6077.646 | 117499.888 | 116225.027 | 1274.861 | 81.276 | 24.860 | 56.415 | 10752.7 | 4895.172 | 5857.523 |
| Afghanistan | 0.337 | 79167.109 | 75468.164 | 3698.945 | 120701.720 | 116531.829 | 4169.891 | 112.812 | 40.368 | 72.444 | 13608.78 | 6359.551 | 7249.233 |
| Bangladesh | 0.492 | 79827.615 | 73818.445 | 6009.170 | 153722.580 | 116246.643 | 37475.936 | 33.322 | 24.393 | 8.929 | 5653.212 | 4883.118 | 770.0941 |
| Bhutan | 0.473 | 73939.039 | 73820.103 | 118.936 | 135528.678 | 116202.221 | 19326.458 | 32.709 | 24.703 | 8.006 | 5605.683 | 4887.228 | 718.455 |
| India | 0.575 | 81633.350 | 73686.048 | 7947.303 | 156143.217 | 111722.425 | 44420.792 | 28.859 | 21.216 | 7.643 | 5524.413 | 4559.593 | 964.8201 |
| Nepal | 0.433 | 73870.737 | 73819.232 | 51.506 | 137364.549 | 116259.308 | 21105.241 | 24.254 | 24.254 | 0.000 | 5049.076 | 5045.964 | 3.112621 |
| Pakistan | 0.504 | 75069.022 | 73821.076 | 1247.946 | 152641.425 | 116250.571 | 36390.854 | 49.074 | 24.095 | 24.979 | 7076.351 | 4859.019 | 2217.332 |
| Angola | 0.454 | 85221.413 | 73816.622 | 11404.791 | 155737.415 | 116226.210 | 39511.204 | 45.854 | 24.828 | 21.027 | 7259.532 | 4895.421 | 2364.111 |
| Central African Republic | 0.309 | 85702.777 | 75551.081 | 10151.696 | 157137.423 | 117088.168 | 40049.255 | 77.079 | 42.261 | 34.818 | 9989.579 | 6521.757 | 3467.823 |
| Congo | 0.583 | 84949.231 | 73681.919 | 11267.312 | 171323.725 | 111276.873 | 60046.853 | 36.421 | 20.640 | 15.781 | 6362.752 | 4476.655 | 1886.098 |
| Democratic Republic of the Congo | 0.383 | 86052.445 | 74527.327 | 11525.118 | 154833.240 | 116209.525 | 38623.715 | 42.053 | 32.665 | 9.388 | 6799.93 | 5658.419 | 1141.511 |
| Equatorial Guinea | 0.658 | 81723.731 | 73458.060 | 8265.671 | 153977.969 | 111000.394 | 42977.576 | 34.352 | 16.261 | 18.091 | 6305.747 | 4150.468 | 2155.279 |
| Gabon | 0.635 | 84644.696 | 73694.999 | 10949.697 | 154344.671 | 110852.224 | 43492.447 | 32.355 | 16.662 | 15.693 | 6174.112 | 4178.319 | 1995.793 |
| Burundi | 0.289 | 80904.861 | 75596.594 | 5308.267 | 160997.226 | 119571.619 | 41425.607 | 51.413 | 47.136 | 4.277 | 7555.469 | 6964.812 | 590.6569 |
| Comoros | 0.476 | 81100.641 | 73813.742 | 7286.899 | 179238.191 | 116219.602 | 63018.588 | 54.177 | 24.848 | 29.328 | 7774.14 | 4888.709 | 2885.431 |
| Djibouti | 0.488 | 79332.124 | 73816.360 | 5515.765 | 162416.757 | 116199.070 | 46217.687 | 40.356 | 24.602 | 15.754 | 6534.654 | 4884.113 | 1650.54 |
| Eritrea | 0.404 | 81207.499 | 74086.194 | 7121.305 | 162125.170 | 116225.782 | 45899.388 | 56.030 | 29.770 | 26.260 | 7926.425 | 5353.987 | 2572.438 |
| Ethiopia | 0.359 | 84329.645 | 74713.251 | 9616.394 | 209591.607 | 116184.596 | 93407.011 | 49.768 | 36.560 | 13.209 | 7318.261 | 5954.683 | 1363.578 |
| Kenya | 0.524 | 83109.791 | 73815.176 | 9294.615 | 179338.925 | 116176.692 | 63162.233 | 31.970 | 23.799 | 8.171 | 5806.369 | 4828.759 | 977.6095 |
| Madagascar | 0.400 | 83485.558 | 74145.533 | 9340.025 | 160252.220 | 116191.188 | 44061.032 | 58.419 | 30.497 | 27.922 | 8384.363 | 5404.765 | 2979.598 |
| Malawi | 0.385 | 82053.658 | 74523.087 | 7530.571 | 175967.125 | 116207.431 | 59759.693 | 55.072 | 32.596 | 22.477 | 7873.847 | 5659.478 | 2214.369 |
| Mauritius | 0.718 | 77819.150 | 70583.171 | 7235.979 | 137653.430 | 110820.070 | 26833.360 | 31.068 | 12.080 | 18.988 | 5988.258 | 3939.016 | 2049.242 |
| Mozambique | 0.326 | 80723.068 | 75554.400 | 5168.668 | 158642.675 | 116981.508 | 41661.167 | 61.057 | 41.173 | 19.885 | 8452.116 | 6430.738 | 2021.378 |
| Rwanda | 0.436 | 80367.739 | 73817.690 | 6550.049 | 163599.434 | 116190.369 | 47409.065 | 48.674 | 25.064 | 23.610 | 7596.874 | 4930.007 | 2666.867 |
| Seychelles | 0.730 | 78730.472 | 70125.347 | 8605.125 | 150552.938 | 110878.474 | 39674.464 | 22.888 | 10.555 | 12.333 | 5111.434 | 3790.193 | 1321.24 |
| Somalia | 0.078 | 82882.791 | 82838.960 | 43.830 | 160485.496 | 158828.251 | 1657.245 | 60.990 | 60.990 | 0.000 | 8405.491 | 8405.491 | 0 |
| United Republic of Tanzania | 0.447 | 83241.215 | 73819.368 | 9421.847 | 159729.774 | 116232.901 | 43496.873 | 65.567 | 24.873 | 40.694 | 9131.899 | 4900.634 | 4231.265 |
| Uganda | 0.423 | 84175.619 | 73824.273 | 10351.346 | 173754.241 | 116202.192 | 57552.048 | 53.641 | 26.377 | 27.265 | 8000.425 | 5100.645 | 2899.78 |
| Zambia | 0.506 | 85111.335 | 73815.138 | 11296.197 | 180426.803 | 116221.960 | 64204.844 | 56.079 | 23.836 | 32.243 | 8063.031 | 4839.772 | 3223.259 |
| Botswana | 0.643 | 76792.454 | 73676.336 | 3116.119 | 152969.844 | 110839.440 | 42130.403 | 30.356 | 16.610 | 13.747 | 5701.91 | 4161.41 | 1540.499 |
| Lesotho | 0.510 | 74491.801 | 73820.519 | 671.282 | 140754.550 | 116221.556 | 24532.994 | 42.268 | 23.833 | 18.435 | 6657.246 | 4837.509 | 1819.737 |
| Namibia | 0.618 | 74569.766 | 73700.758 | 869.008 | 139852.398 | 110865.486 | 28986.912 | 28.540 | 18.061 | 10.479 | 5414.369 | 4282.074 | 1132.296 |
| South Africa | 0.680 | 76556.611 | 72808.588 | 3748.022 | 160941.917 | 110867.437 | 50074.480 | 28.537 | 15.016 | 13.521 | 5406.127 | 4136.316 | 1269.811 |
| Eswatini | 0.585 | 77663.871 | 73703.111 | 3960.760 | 154796.048 | 111166.913 | 43629.136 | 37.189 | 20.356 | 16.833 | 6373.152 | 4480.446 | 1892.707 |
| Zimbabwe | 0.474 | 75148.671 | 73817.935 | 1330.736 | 149116.443 | 116202.934 | 32913.509 | 44.074 | 24.666 | 19.408 | 6502.008 | 4893.414 | 1608.594 |
| Benin | 0.373 | 82507.864 | 74622.017 | 7885.847 | 164126.185 | 116222.248 | 47903.938 | 72.809 | 33.690 | 39.119 | 9586.74 | 5740.117 | 3846.623 |
| Burkina Faso | 0.285 | 85095.441 | 75585.544 | 9509.897 | 150123.271 | 119579.789 | 30543.482 | 91.578 | 47.347 | 44.231 | 11332.54 | 6974.428 | 4358.111 |
| Cameroon | 0.480 | 80505.427 | 73820.723 | 6684.703 | 159433.752 | 116201.517 | 43232.235 | 58.551 | 24.742 | 33.809 | 8072.497 | 4896.193 | 3176.304 |
| Cabo Verde | 0.534 | 77223.127 | 73814.720 | 3408.406 | 161564.006 | 115939.112 | 45624.894 | 22.445 | 22.445 | 0.000 | 4971.162 | 4830.495 | 140.6676 |
| Chad | 0.240 | 81921.833 | 76028.831 | 5893.002 | 163988.788 | 119572.435 | 44416.353 | 72.158 | 55.890 | 16.268 | 9341.349 | 7898.649 | 1442.7 |
| Côte d'Ivoire | 0.426 | 81356.976 | 73816.164 | 7540.812 | 164530.506 | 116251.597 | 48278.908 | 57.192 | 26.584 | 30.608 | 8141.412 | 5085.271 | 3056.141 |
| Gambia | 0.410 | 79803.628 | 73938.191 | 5865.436 | 155897.444 | 116207.222 | 39690.222 | 46.647 | 28.353 | 18.293 | 7135.521 | 5267.764 | 1867.757 |
| Ghana | 0.565 | 80799.997 | 73813.510 | 6986.486 | 154323.113 | 112285.760 | 42037.352 | 49.429 | 22.153 | 27.276 | 7288.622 | 4650.578 | 2638.044 |
| Guinea | 0.336 | 83594.806 | 75467.982 | 8126.824 | 165006.353 | 116549.901 | 48456.452 | 76.235 | 40.302 | 35.933 | 9928.388 | 6366.145 | 3562.243 |
| Guinea-Bissau | 0.353 | 81017.116 | 74910.910 | 6106.206 | 165898.132 | 116253.602 | 49644.530 | 59.506 | 37.807 | 21.699 | 8137.433 | 6023.531 | 2113.902 |
| Liberia | 0.352 | 82745.593 | 74850.987 | 7894.606 | 163370.420 | 116222.732 | 47147.688 | 57.792 | 37.659 | 20.133 | 8150.876 | 6028.265 | 2122.61 |
| Mali | 0.269 | 83212.776 | 75660.210 | 7552.567 | 174793.594 | 119566.642 | 55226.952 | 76.193 | 50.666 | 25.527 | 9677.335 | 7338.213 | 2339.123 |
| Mauritania | 0.499 | 80878.783 | 73816.228 | 7062.555 | 162786.403 | 116217.180 | 46569.224 | 34.436 | 24.110 | 10.325 | 6019.538 | 4858.034 | 1161.504 |
| Niger | 0.168 | 84126.761 | 82843.105 | 1283.656 | 160476.660 | 158825.934 | 1650.726 | 54.953 | 54.953 | 0.000 | 7929.242 | 7929.242 | 0 |
| Nigeria | 0.503 | 86365.540 | 73819.252 | 12546.288 | 177214.146 | 116247.537 | 60966.608 | 72.438 | 24.046 | 48.392 | 9689.88 | 4861.607 | 4828.273 |
| Sao Tome and Principe | 0.505 | 81226.861 | 73822.675 | 7404.187 | 148077.656 | 116220.116 | 31857.540 | 31.430 | 24.042 | 7.388 | 5789.627 | 4867.103 | 922.524 |
| Senegal | 0.408 | 81893.638 | 74001.764 | 7891.874 | 168279.742 | 116245.965 | 52033.777 | 47.126 | 29.608 | 17.518 | 7163.173 | 5310.116 | 1853.057 |
| Sierra Leone | 0.359 | 84331.249 | 74742.406 | 9588.843 | 156251.413 | 116265.722 | 39985.690 | 77.990 | 36.412 | 41.578 | 10126.43 | 5987.07 | 4139.355 |
| Togo | 0.409 | 81522.385 | 73983.653 | 7538.732 | 153863.308 | 116201.763 | 37661.546 | 54.385 | 28.617 | 25.768 | 7950.533 | 5268.206 | 2682.328 |
| American Samoa | 0.724 | 79042.507 | 70356.353 | 8686.154 | 144748.618 | 110914.635 | 33833.983 | 25.028 | 11.208 | 13.819 | 5082.739 | 3910.619 | 1172.12 |
| Bermuda | 0.821 | 81266.029 | 67620.438 | 13645.591 | 161075.651 | 105715.267 | 55360.384 | 14.979 | 7.649 | 7.330 | 4928.98 | 3559.657 | 1369.324 |
| Cook Islands | 0.779 | 79310.434 | 69155.917 | 10154.517 | 150099.856 | 107465.371 | 42634.485 | 10.764 | 7.803 | 2.961 | 3897.921 | 3515.791 | 382.1301 |
| Greenland | 0.826 | 73530.058 | 67570.602 | 5959.456 | 121059.535 | 105381.459 | 15678.076 | 20.011 | 7.615 | 12.396 | 5900.677 | 3534.128 | 2366.549 |
| Guam | 0.804 | 79408.552 | 67879.160 | 11529.393 | 151044.347 | 105549.336 | 45495.011 | 20.639 | 7.756 | 12.883 | 4855.643 | 3562.365 | 1293.278 |
| Monaco | 0.908 | 74302.158 | 67502.258 | 6799.899 | 133235.285 | 105824.881 | 27410.404 | 16.174 | 7.221 | 8.953 | 5463.214 | 3542.125 | 1921.089 |
| Nauru | 0.625 | 79612.478 | 73696.566 | 5915.911 | 149555.604 | 110901.155 | 38654.449 | 49.223 | 17.023 | 32.200 | 7120.858 | 4194.915 | 2925.943 |
| Niue | 0.726 | 79409.686 | 70414.710 | 8994.976 | 155236.112 | 110924.314 | 44311.799 | 114.848 | 10.949 | 103.899 | 12743.23 | 3892.057 | 8851.169 |
| Northern Mariana Islands | 0.772 | 79772.904 | 69340.774 | 10432.130 | 156352.848 | 108100.242 | 48252.606 | 13.109 | 8.583 | 4.525 | 4160.919 | 3534.89 | 626.0285 |
| Palau | 0.754 | 79749.387 | 69762.995 | 9986.392 | 154682.216 | 109221.517 | 45460.699 | 31.989 | 8.159 | 23.830 | 5632.789 | 3629.595 | 2003.194 |
| Puerto Rico | 0.826 | 82264.801 | 67647.410 | 14617.391 | 150554.085 | 105607.198 | 44946.887 | 16.463 | 7.657 | 8.805 | 5193.574 | 3554.879 | 1638.695 |
| Saint Kitts and Nevis | 0.755 | 81412.153 | 69629.729 | 11782.424 | 159536.572 | 109483.396 | 50053.176 | 33.479 | 8.984 | 24.495 | 6451.381 | 3688.976 | 2762.405 |
| San Marino | 0.888 | 74394.668 | 67505.171 | 6889.497 | 125890.008 | 105586.727 | 20303.281 | 7.207 | 6.853 | 0.354 | 4676.956 | 3549.496 | 1127.46 |
| Tokelau | 0.686 | 79527.606 | 72146.626 | 7380.980 | 149582.653 | 110790.858 | 38791.795 | 127.725 | 14.744 | 112.981 | 13826.31 | 4108.958 | 9717.355 |
| Tuvalu | 0.577 | 79970.497 | 73700.523 | 6269.975 | 154106.932 | 111427.997 | 42678.934 | 31.680 | 21.231 | 10.448 | 5582.484 | 4566.79 | 1015.694 |
| United States Virgin Islands | 0.822 | 81692.832 | 67759.622 | 13933.210 | 160980.780 | 105444.006 | 55536.774 | 15.443 | 7.511 | 7.931 | 4964.671 | 3546.772 | 1417.899 |
| South Sudan | 0.278 | 82074.606 | 75591.533 | 6483.074 | 174975.600 | 119577.257 | 55398.343 | 100.813 | 48.966 | 51.847 | 11881.73 | 7150.757 | 4730.972 |
| Sudan | 0.542 | 79931.774 | 73814.993 | 6116.781 | 120688.974 | 114344.313 | 6344.661 | 85.732 | 23.089 | 62.643 | 11219.34 | 4825.985 | 6393.359 |

Supplementary Table 8: Results of Age-Period-Cohort Analysis of NCDs among Global Children and Adolescents.

|  |  |  | **ASPR** | | | **ASIR** | | | **ASMR** | | | **ASDR** | | |
| --- | --- | --- | --- | --- | --- | --- | --- | --- | --- | --- | --- | --- | --- | --- |
| **Age** | **Type** | **Sex** | **Value** | **Low** | **Up** | **Value** | **Low** | **Up** | **Value** | **Low** | **Up** | **Value** | **Low** | **Up** |
| 2.5 | Age effect | Both | 68546.283 | 68418.322 | 68674.484 | 110686.488 | 108656.950 | 112753.936 | 173.560 | 170.080 | 177.112 | 17203.753 | 16519.237 | 17916.634 |
| 7.5 | Age effect | Both | 80565.538 | 80429.070 | 80702.236 | 204300.056 | 201177.226 | 207471.360 | 17.742 | 17.226 | 18.273 | 3967.009 | 3762.124 | 4183.051 |
| 12.5 | Age effect | Both | 85069.163 | 84927.234 | 85211.329 | 150188.060 | 147758.440 | 152657.631 | 15.124 | 14.665 | 15.596 | 5124.148 | 4875.494 | 5385.483 |
| 17.5 | Age effect | Both | 90798.778 | 90640.910 | 90956.922 | 136844.498 | 134460.476 | 139270.789 | 24.141 | 23.431 | 24.874 | 7204.632 | 6856.439 | 7570.508 |
| 1977 | Cohort RR | Both | 0.992 | 0.989 | 0.996 | 0.943 | 0.909 | 0.979 | 1.189 | 1.121 | 1.261 | 0.981 | 0.888 | 1.083 |
| 1982 | Cohort RR | Both | 0.995 | 0.993 | 0.998 | 0.971 | 0.947 | 0.996 | 1.189 | 1.141 | 1.240 | 1.005 | 0.937 | 1.078 |
| 1987 | Cohort RR | Both | 0.996 | 0.994 | 0.998 | 0.983 | 0.964 | 1.003 | 1.157 | 1.115 | 1.201 | 1.016 | 0.954 | 1.082 |
| 1992 | Cohort RR | Both | 1.000 | 0.998 | 1.002 | 0.994 | 0.976 | 1.012 | 1.080 | 1.048 | 1.113 | 1.037 | 0.983 | 1.094 |
| 1997 | Cohort RR | Both | 1.000 | 1.000 | 1.000 | 1.000 | 1.000 | 1.000 | 1.000 | 1.000 | 1.000 | 1.000 | 1.000 | 1.000 |
| 2002 | Cohort RR | Both | 0.994 | 0.992 | 0.996 | 0.997 | 0.979 | 1.016 | 0.927 | 0.899 | 0.955 | 0.944 | 0.894 | 0.996 |
| 2007 | Cohort RR | Both | 0.986 | 0.983 | 0.988 | 0.987 | 0.967 | 1.007 | 0.825 | 0.799 | 0.852 | 0.874 | 0.825 | 0.926 |
| 2012 | Cohort RR | Both | 0.979 | 0.977 | 0.982 | 0.988 | 0.965 | 1.011 | 0.715 | 0.693 | 0.738 | 0.766 | 0.720 | 0.815 |
| 2017 | Cohort RR | Both | 0.978 | 0.975 | 0.982 | 0.993 | 0.957 | 1.029 | 0.609 | 0.588 | 0.631 | 0.620 | 0.574 | 0.669 |
| 1994.5 | Period RR | Both | 1.008 | 1.006 | 1.011 | 1.014 | 0.994 | 1.035 | 1.262 | 1.226 | 1.299 | 1.202 | 1.139 | 1.269 |
| 1999.5 | Period RR | Both | 1.004 | 1.002 | 1.006 | 1.002 | 0.984 | 1.021 | 1.121 | 1.088 | 1.155 | 1.085 | 1.028 | 1.146 |
| 2004.5 | Period RR | Both | 1.000 | 1.000 | 1.000 | 1.000 | 1.000 | 1.000 | 1.000 | 1.000 | 1.000 | 1.000 | 1.000 | 1.000 |
| 2009.5 | Period RR | Both | 0.997 | 0.995 | 0.998 | 1.000 | 0.982 | 1.019 | 0.928 | 0.899 | 0.958 | 0.945 | 0.894 | 0.999 |
| 2014.5 | Period RR | Both | 0.995 | 0.993 | 0.997 | 1.020 | 1.001 | 1.039 | 0.889 | 0.860 | 0.919 | 0.939 | 0.887 | 0.993 |
| 2019.5 | Period RR | Both | 0.998 | 0.996 | 1.000 | 1.028 | 1.008 | 1.049 | 0.819 | 0.791 | 0.848 | 0.952 | 0.898 | 1.008 |
| 2.5 | Local Drifts | Both | -0.103 | -0.115 | -0.091 | -0.030 | -0.146 | 0.086 | -2.252 | -2.335 | -2.168 | -1.953 | -2.172 | -1.734 |
| 7.5 | Local Drifts | Both | -0.078 | -0.087 | -0.069 | 0.000 | -0.077 | 0.077 | -1.863 | -1.993 | -1.733 | -1.127 | -1.363 | -0.892 |
| 12.5 | Local Drifts | Both | -0.031 | -0.040 | -0.023 | 0.074 | -0.009 | 0.157 | -1.459 | -1.618 | -1.301 | -0.544 | -0.800 | -0.286 |
| 17.5 | Local Drifts | Both | 0.015 | 0.004 | 0.027 | 0.215 | 0.097 | 0.334 | -1.044 | -1.236 | -0.852 | -0.106 | -0.421 | 0.210 |
| 1 | Net drift | Both | -0.049 | -0.055 | -0.042 | 0.069 | 0.007 | 0.130 | -1.661 | -1.760 | -1.561 | -0.945 | -1.117 | -0.772 |

Supplementary Table 9: Bayesian Age-Period-Cohort (BAPC) Prediction Results of Age-Standardized Rates of NCDs among Global Children and Adolescents by Different Age Groups.

|  |  | ASPR | | ASIR | | ASMR | | ASDR | |
| --- | --- | --- | --- | --- | --- | --- | --- | --- | --- |
| Time | Age | Value | SD | Value | SD | Value | SD | Value | SD |
| 2021 | <5 years | 67645.48 | 3.21 | 113052.2 | 4.14 | 97.14 | 0.12 | 10314.57 | 1.25 |
| 2022 | <5 years | 67718.1 | 1013.66 | 112959.5 | 2300.26 | 94.32 | 2.7 | 9885.64 | 411.46 |
| 2023 | <5 years | 67821.85 | 1275.64 | 113543.3 | 2812.09 | 90.89 | 3.14 | 9377.8 | 468.83 |
| 2024 | <5 years | 67928.29 | 1604.63 | 114136.1 | 3453.76 | 87.6 | 3.66 | 8897.37 | 531.2 |
| 2025 | <5 years | 68038.03 | 1989.06 | 114739.2 | 4207.61 | 84.43 | 4.21 | 8443.06 | 596.2 |
| 2026 | <5 years | 68151.69 | 2420.65 | 115354.1 | 5059.96 | 81.39 | 4.8 | 8013.62 | 661.99 |
| 2027 | <5 years | 68269.9 | 2893.85 | 115982.2 | 6001.24 | 78.47 | 5.4 | 7607.82 | 727.25 |
| 2028 | <5 years | 68393.28 | 3404.84 | 116625.2 | 7024.99 | 75.66 | 6.01 | 7224.51 | 791.04 |
| 2029 | <5 years | 68522.47 | 3951.01 | 117284.4 | 8126.9 | 72.97 | 6.62 | 6862.53 | 852.73 |
| 2030 | <5 years | 68658.11 | 4530.49 | 117961.7 | 9304.22 | 70.39 | 7.23 | 6520.81 | 911.92 |
| 2031 | <5 years | 68800.85 | 5141.98 | 118658.5 | 10555.31 | 67.91 | 7.82 | 6198.29 | 968.37 |
| 2032 | <5 years | 68951.36 | 5784.56 | 119376.5 | 11879.4 | 65.53 | 8.41 | 5893.99 | 1021.98 |
| 2033 | <5 years | 69110.28 | 6457.63 | 120117.6 | 13276.4 | 63.26 | 8.98 | 5606.94 | 1072.75 |
| 2034 | <5 years | 69278.3 | 7160.85 | 120883.4 | 14746.8 | 61.07 | 9.54 | 5336.22 | 1120.73 |
| 2035 | <5 years | 69456.11 | 7894.1 | 121675.9 | 16291.62 | 58.98 | 10.09 | 5080.96 | 1166.04 |
| 2021 | 5-9 years | 79159.91 | 3.39 | 201406.1 | 5.41 | 11.4 | 0.04 | 3263.86 | 0.69 |
| 2022 | 5-9 years | 79074.09 | 928.53 | 204219.3 | 3341.35 | 11.5 | 0.27 | 2956.42 | 103.81 |
| 2023 | 5-9 years | 79120.21 | 979.32 | 204836.3 | 3514.71 | 11.11 | 0.29 | 2814.25 | 107.82 |
| 2024 | 5-9 years | 79209.82 | 1028.31 | 205651.2 | 3696.34 | 10.72 | 0.3 | 2673.19 | 111.06 |
| 2025 | 5-9 years | 79324.04 | 1090.35 | 206627 | 3933.61 | 10.33 | 0.31 | 2536.05 | 114.48 |
| 2026 | 5-9 years | 79441.89 | 1197.4 | 207681.2 | 4313.23 | 9.95 | 0.33 | 2405.35 | 119.21 |
| 2027 | 5-9 years | 79561.49 | 1388.18 | 208746.8 | 4929.46 | 9.59 | 0.35 | 2281.6 | 126.14 |
| 2028 | 5-9 years | 79683.54 | 1668.47 | 209826.5 | 5801.22 | 9.24 | 0.39 | 2164.47 | 135.11 |
| 2029 | 5-9 years | 79808.76 | 2030 | 210922.8 | 6914.67 | 8.91 | 0.43 | 2053.66 | 145.72 |
| 2030 | 5-9 years | 79937.87 | 2461.16 | 212038.2 | 8244.71 | 8.59 | 0.48 | 1948.87 | 157.49 |
| 2031 | 5-9 years | 80071.59 | 2951.99 | 213175.4 | 9767.41 | 8.28 | 0.53 | 1849.81 | 169.97 |
| 2032 | 5-9 years | 80210.65 | 3495.12 | 214337.1 | 11463.98 | 7.98 | 0.59 | 1756.2 | 182.79 |
| 2033 | 5-9 years | 80355.78 | 4085.27 | 215526.2 | 13320.76 | 7.69 | 0.64 | 1667.77 | 195.64 |
| 2034 | 5-9 years | 80507.74 | 4718.73 | 216745.4 | 15328.22 | 7.42 | 0.7 | 1584.26 | 208.32 |
| 2035 | 5-9 years | 80667.29 | 5392.84 | 217997.8 | 17479.97 | 7.16 | 0.76 | 1505.43 | 220.68 |
| 2021 | 10-14 years | 84121.87 | 3.55 | 145967.8 | 4.68 | 12.26 | 0.04 | 4895.18 | 0.86 |
| 2022 | 10-14 years | 83938.04 | 974.64 | 149344.4 | 2422.48 | 11.57 | 0.27 | 4604.75 | 161.75 |
| 2023 | 10-14 years | 83817.72 | 1025.25 | 149389.8 | 2539.02 | 11.18 | 0.29 | 4474.19 | 171.7 |
| 2024 | 10-14 years | 83704.69 | 1072.45 | 149426.2 | 2650.35 | 10.8 | 0.3 | 4332.58 | 179.72 |
| 2025 | 10-14 years | 83612.47 | 1119.1 | 149478.5 | 2761.45 | 10.43 | 0.31 | 4178.06 | 185.81 |
| 2026 | 10-14 years | 83553.55 | 1169.05 | 149615.7 | 2879.62 | 10.08 | 0.31 | 4009.63 | 190.05 |
| 2027 | 10-14 years | 83545.57 | 1223.63 | 149914.4 | 3007.13 | 9.74 | 0.32 | 3829.48 | 192.6 |
| 2028 | 10-14 years | 83594.43 | 1275.81 | 150367.8 | 3134 | 9.41 | 0.33 | 3645.43 | 193.75 |
| 2029 | 10-14 years | 83689.22 | 1326.49 | 150966.4 | 3266.3 | 9.08 | 0.33 | 3462.79 | 194.09 |
| 2030 | 10-14 years | 83810.01 | 1388.57 | 151683 | 3433.4 | 8.75 | 0.34 | 3285.23 | 194.66 |
| 2031 | 10-14 years | 83934.64 | 1490.99 | 152457.3 | 3690.8 | 8.43 | 0.35 | 3116.02 | 196.74 |
| 2032 | 10-14 years | 84061.11 | 1671.95 | 153239.9 | 4103.77 | 8.12 | 0.36 | 2955.78 | 201.43 |
| 2033 | 10-14 years | 84190.17 | 1942.59 | 154032.9 | 4694.11 | 7.83 | 0.38 | 2804.13 | 208.75 |
| 2034 | 10-14 years | 84322.59 | 2299.64 | 154838 | 5460.81 | 7.55 | 0.41 | 2660.64 | 218.42 |
| 2035 | 10-14 years | 84459.12 | 2733.55 | 155657.2 | 6391.07 | 7.27 | 0.45 | 2524.95 | 229.95 |
| 2021 | 15-19 years | 90588.94 | 3.81 | 140538.8 | 4.75 | 22.22 | 0.06 | 7141.54 | 1.07 |
| 2022 | 15-19 years | 90305.93 | 1045.42 | 136588.1 | 2211.08 | 21.29 | 0.5 | 7216.26 | 253.58 |
| 2023 | 15-19 years | 90140.99 | 1098.38 | 136195.4 | 2308.31 | 20.75 | 0.53 | 7104.98 | 272.47 |
| 2024 | 15-19 years | 89989.66 | 1148.09 | 135871.8 | 2402.2 | 20.17 | 0.55 | 6979.63 | 289.09 |
| 2025 | 15-19 years | 89858.54 | 1196.99 | 135643.5 | 2496.4 | 19.57 | 0.57 | 6841.2 | 303.8 |
| 2026 | 15-19 years | 89735.33 | 1248.35 | 135533.5 | 2596.23 | 18.96 | 0.59 | 6687.48 | 316.84 |
| 2027 | 15-19 years | 89609.19 | 1303.59 | 135550.1 | 2704.29 | 18.34 | 0.6 | 6517.2 | 328.23 |
| 2028 | 15-19 years | 89480.89 | 1356.71 | 135591.7 | 2810.82 | 17.72 | 0.61 | 6332.61 | 337.48 |
| 2029 | 15-19 years | 89360.38 | 1407 | 135625.2 | 2913.48 | 17.11 | 0.62 | 6132.38 | 344.32 |
| 2030 | 15-19 years | 89262.07 | 1457.02 | 135673.1 | 3016.26 | 16.52 | 0.63 | 5913.85 | 348.58 |
| 2031 | 15-19 years | 89199.34 | 1510.33 | 135798 | 3125.23 | 15.97 | 0.63 | 5675.61 | 350.21 |
| 2032 | 15-19 years | 89190.98 | 1568.27 | 136069.6 | 3242.66 | 15.43 | 0.64 | 5420.78 | 349.42 |
| 2033 | 15-19 years | 89243.3 | 1624.18 | 136481.5 | 3360.31 | 14.91 | 0.64 | 5160.41 | 346.7 |
| 2034 | 15-19 years | 89344.65 | 1678.61 | 137025.2 | 3482.56 | 14.38 | 0.65 | 4902.03 | 342.91 |
| 2035 | 15-19 years | 89473.75 | 1743.15 | 137676.1 | 3632.2 | 13.87 | 0.65 | 4650.81 | 339.34 |

ASPR：Age-standardized Prevalence Rate；ASIR：Age-standardized Incidence Rate；ASMR：Age-standardized Mortality Rate；ASDR：Age-standardized DALY Rate.

**
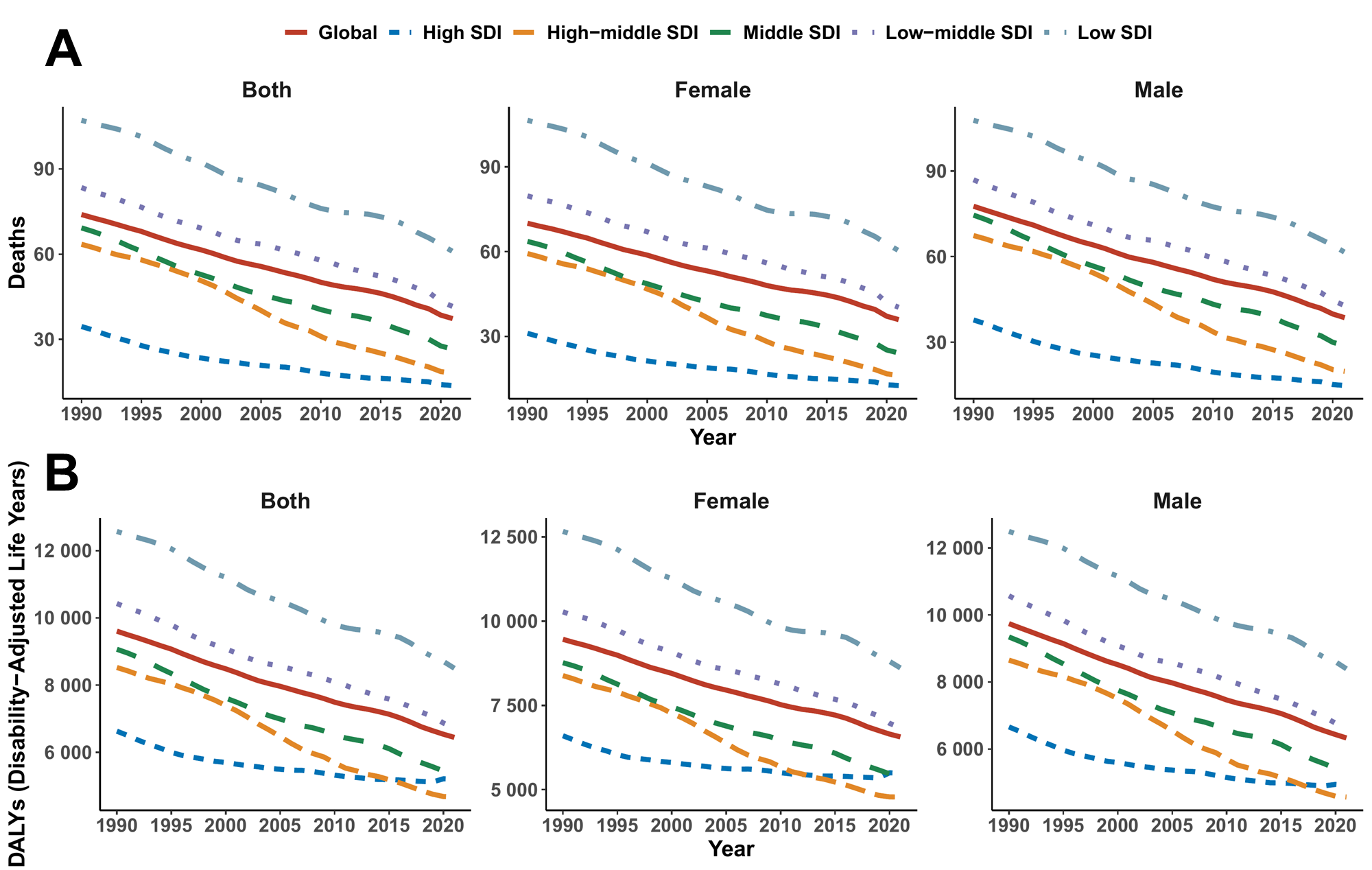
**

Figure S1: Trends in age-standardized rates of non-communicable diseases (NCDs) among children and adolescents globally from 1990 to 2021. Panel A: Age-standardized mortality rate (ASMR). Panel B: Age-standardized disability rate (ASDR).

**
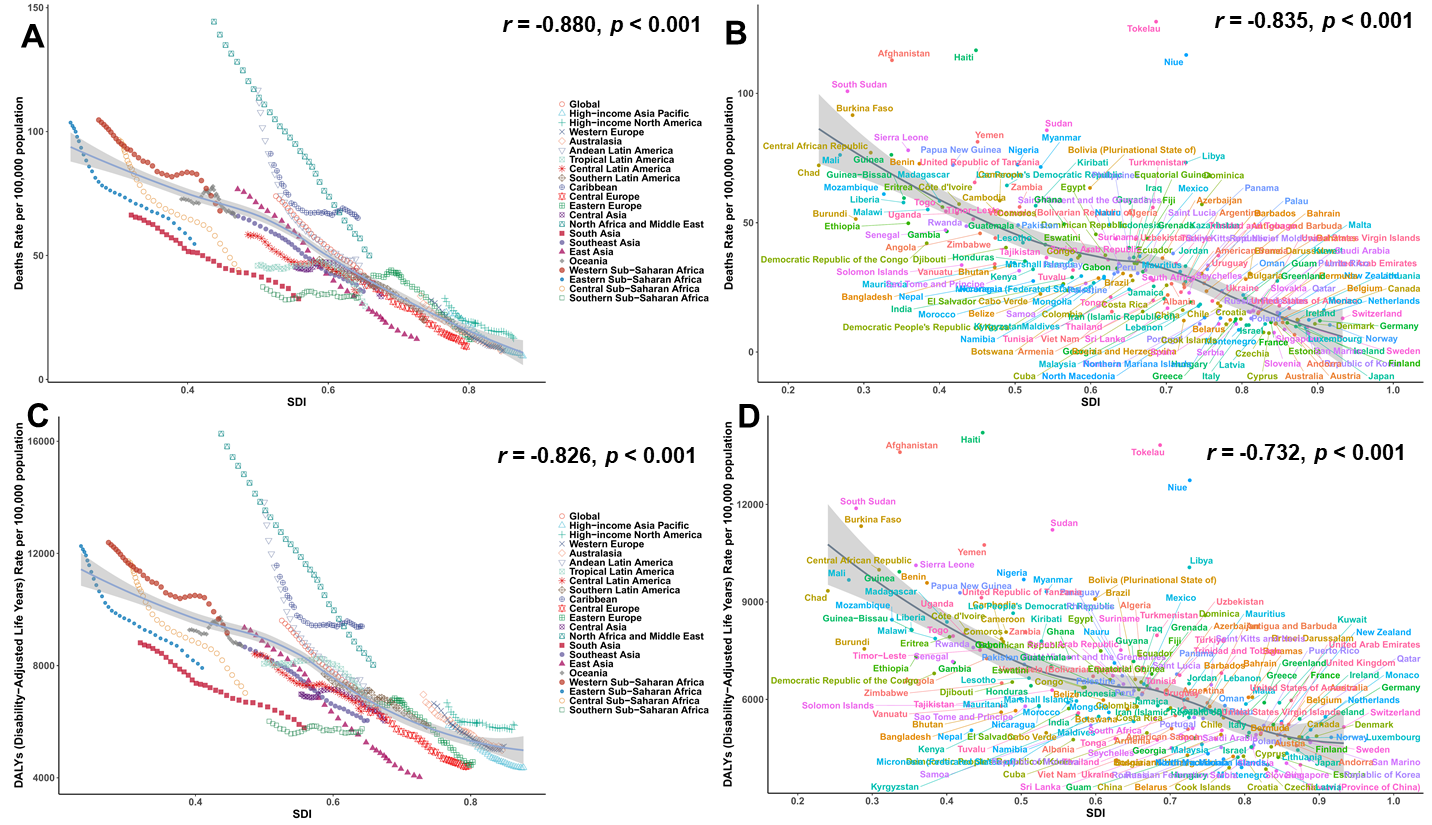
**

Figure S2: Spearman correlation analysis of age-standardized rates of NCDs among children and adolescents with the Socio-demographic Index (SDI) for 21 global regions and 204 countries from 1990 to 2021. Panel A: ASMR and SDI for 21 global regions. Panel B: ASDR and SDI for 21 global regions. Panel C: ASMR and SDI for 204 countries. Panel D: ASDR and SDI for 204 countries.

**
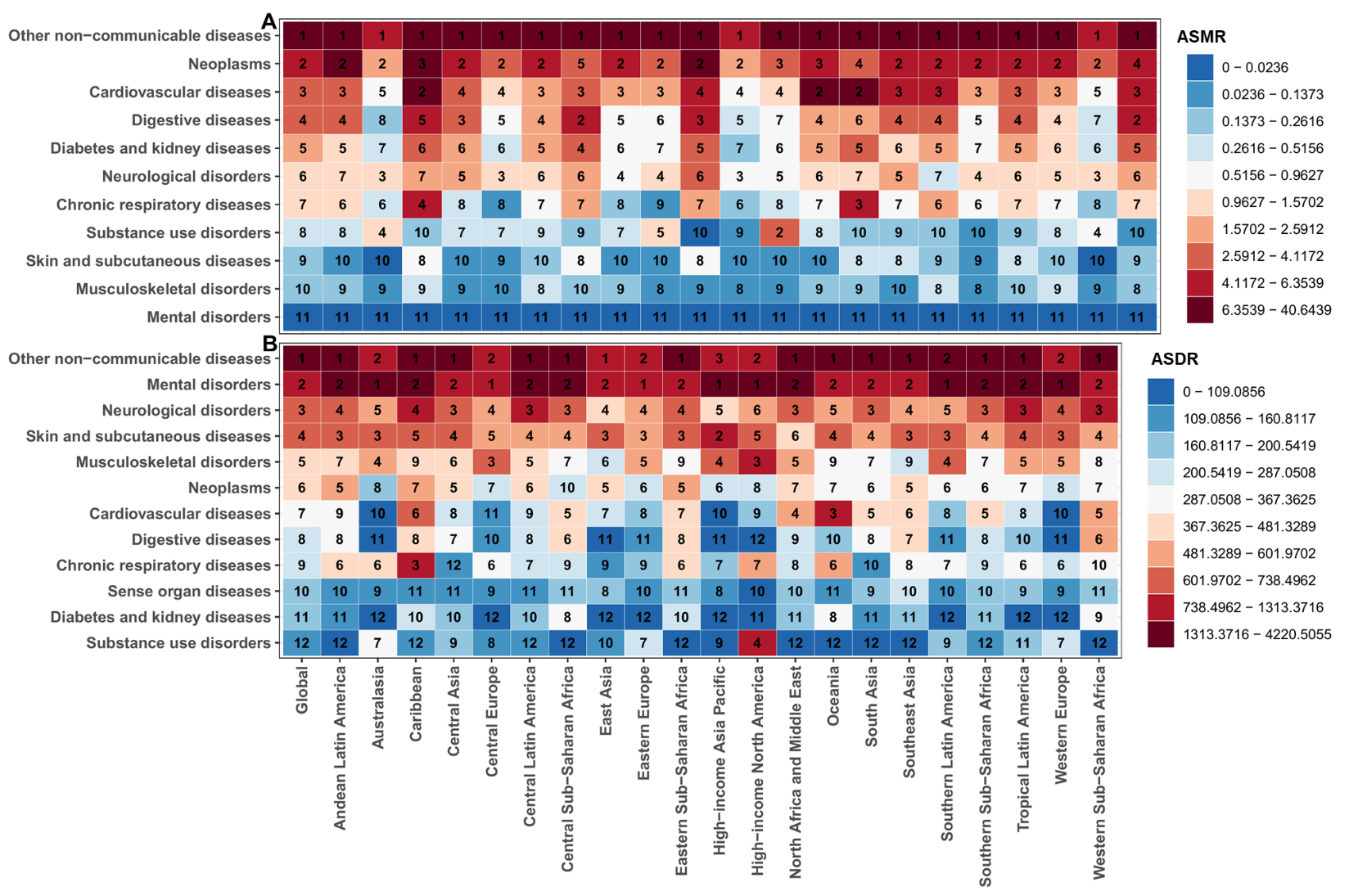
**

Figure S3: Heatmaps of age-standardized rates of second-level NCDs among children and adolescents globally and in 21 regions in 2021. Panel A: ASMR. Panel B: ASDR.


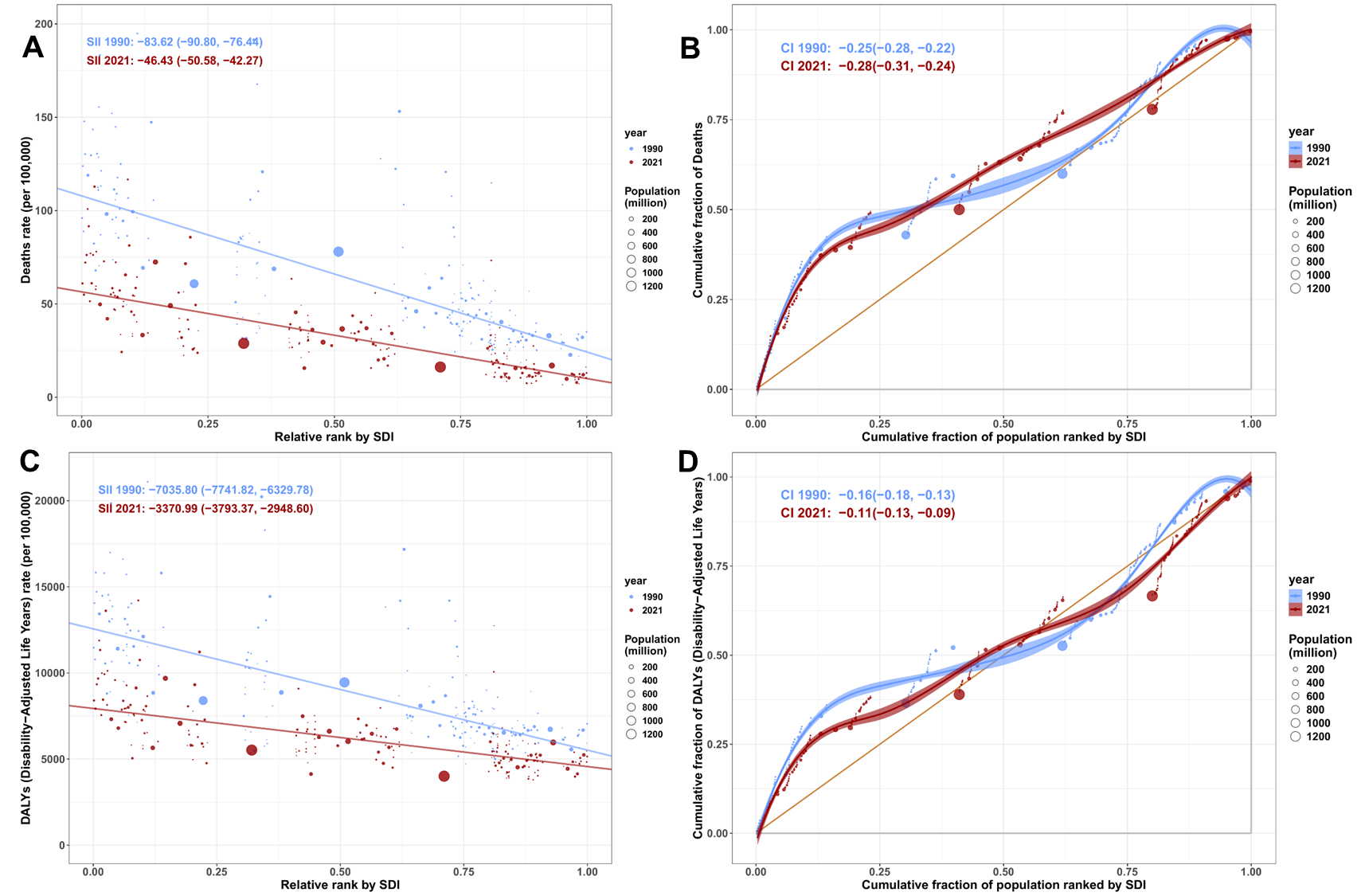


Figure S4: Health inequality regression and concentration curves of age-standardized prevalence and incidence rates of NCDs among children and adolescents globally in 1990 and 2021. Panels A and C show the slope index of inequality, depicting the relationship between the Socio-demographic Index (SDI) and age-standardized rates, with points representing countries and regions weighted by population size. Panels B and D show the concentration index, quantifying relative inequality by integrating the area under the Lorenz curve, aligning the distribution of ASMR and ASDR with the population distribution stratified by SDI. Blue represents data from 1990, and red represents data from 2021.


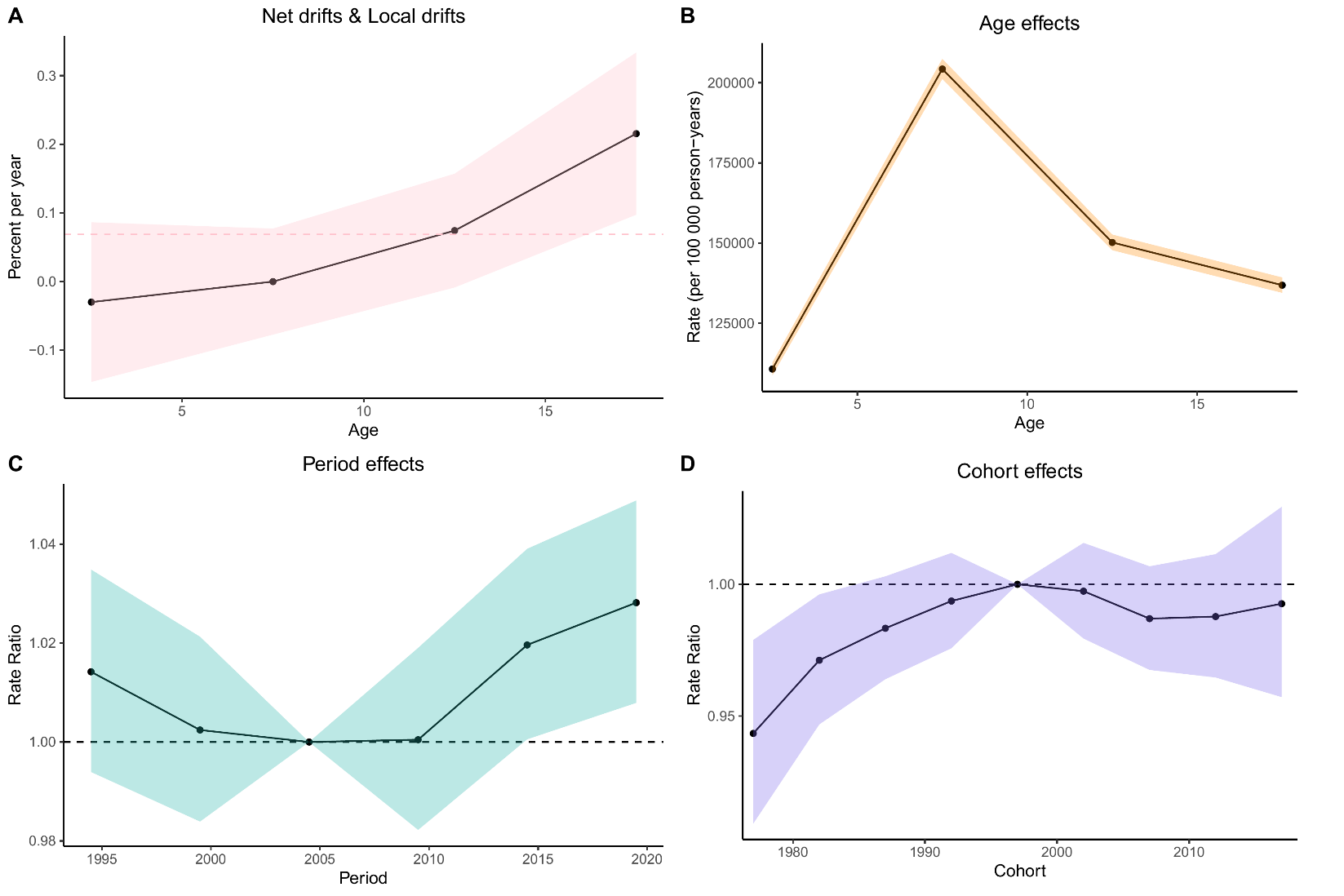


Figure S5: Age-period-cohort effect analysis of age-standardized incidence rates of NCDs among children and adolescents globally.


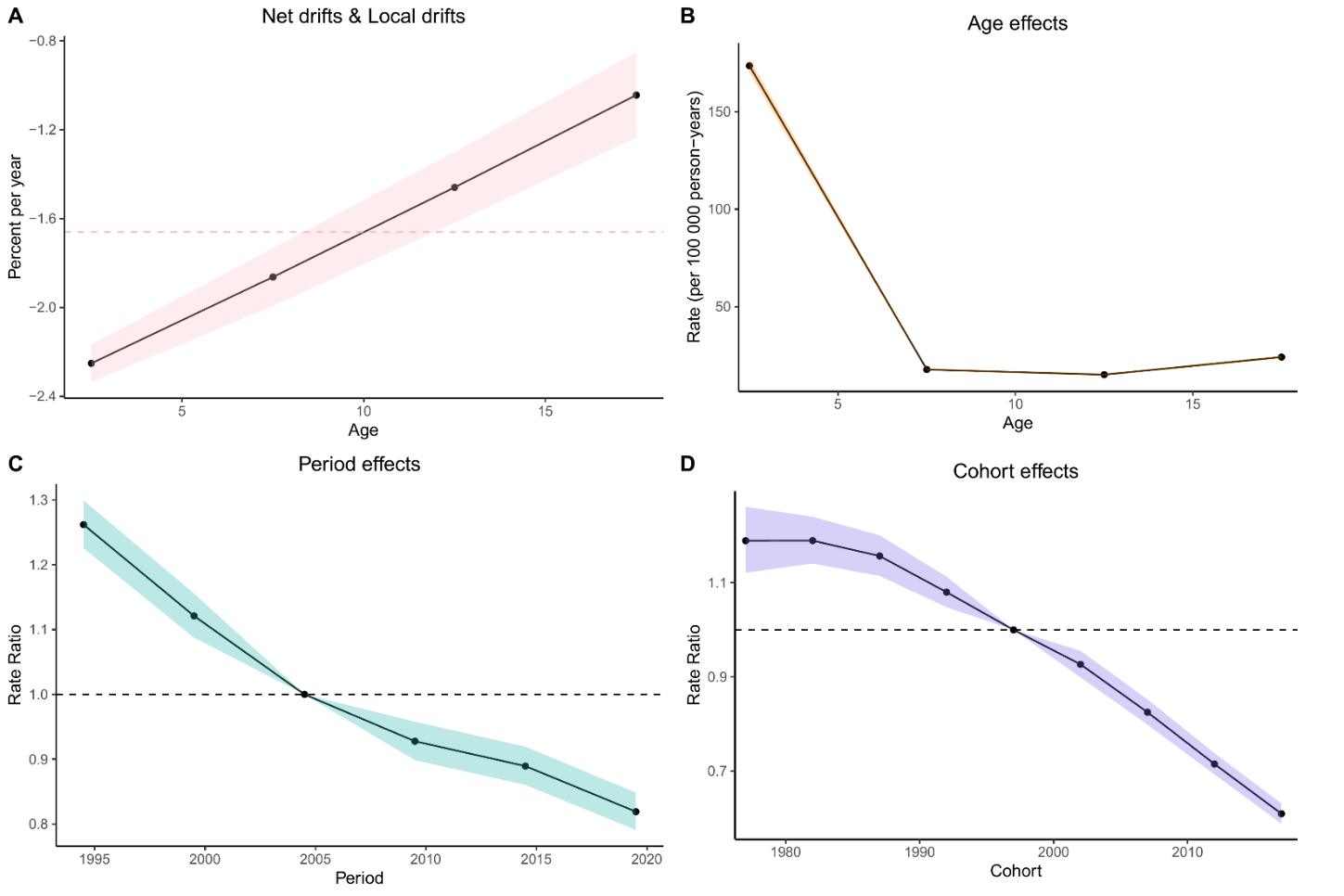


Figure S6: Age-period-cohort effect analysis of age-standardized mortality rates of NCDs among children and adolescents globally.

**
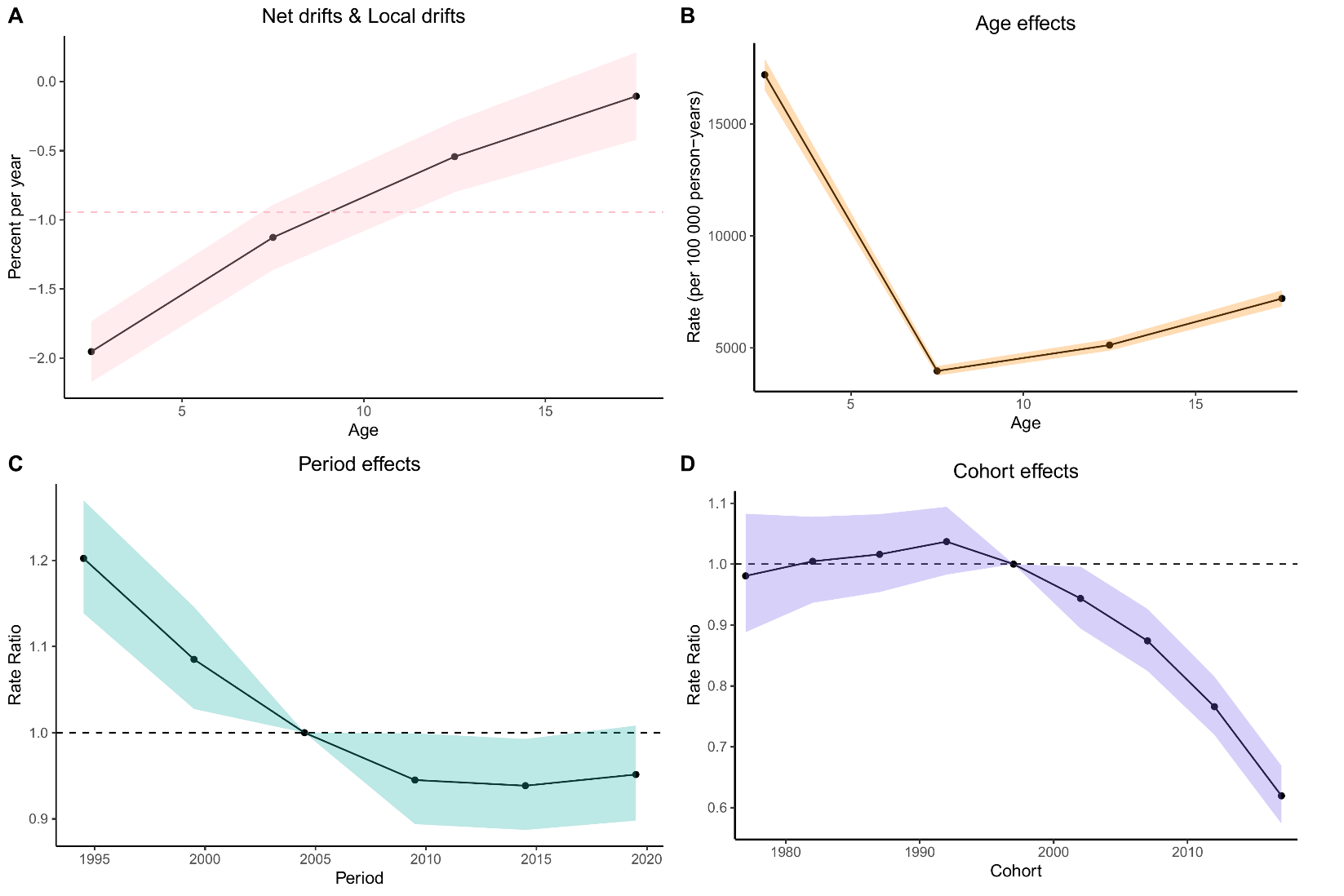
**

Figure S7: Age-period-cohort effect analysis of age-standardized disability-adjusted life-years (DALY) rates of NCDs among children and adolescents globally.
